# Thermocatalytic Transformation of Nitriles Utilizing Pristine and Calcined ZnCr Layered Double Hydroxides for the Synthesis of Various Tetrazole- and Kynurenic Acid-Based Drug Candidates

**DOI:** 10.1021/acsomega.5c12277

**Published:** 2026-03-04

**Authors:** Hiba Alsoliman, Márton Szabados, Péter Bélteky, Zoltán Kónya, István Szatmári, Rebeka Mészáros

**Affiliations:** † Institute of Pharmaceutical Chemistry, 37442 University of Szeged, Eötvös utca 6, Szeged H-6720, Hungary; ‡ Department of Molecular and Analytical Chemistry, University of Szeged, Dóm tér 7-8, Szeged H-6720, Hungary; § Material and Solution Structure Research Group, Institute of Chemistry, University of Szeged, Aradi vértanúk tere 1, Szeged H-6720, Hungary; ∥ Department of Applied and Environmental Chemistry, University of Szeged, Rerrich B. tér 1, Szeged H-6720, Hungary; ⊥ HUN-REN-SZTE Reaction Kinetics and Surface Chemistry Research Group, Rerrich B. tér 1, Szeged H-6720, Hungary; # HUN-REN-SZTE Stereochemistry Research Group, Eötvös utca 6, Szeged H-6720, Hungary

## Abstract

For the first time, Zn/Cr-containing layered double hydroxides (LDH) and mixed metal oxides (MMO) were applied in the synthesis of 5-substituted 1*H*-tetrazole heterocycles from different aromatic nitriles and TMSN_3_ (trimethylsilyl azide) as a less explosive/toxic and easily recoverable azide source. Effects of the nitrile concentration, reaction time, temperature, catalyst loading, and amount of N_3_^–^ source were carefully investigated to achieve high yields and selective tetrazole formation under the most sustainable conditions. Both Zn_*x*_Cr-LDH and -MMO (prepared based on thermogravimetric analysis) catalyst forms were efficient in the reaction (achieving between 60 and 95% conversion), and Zn_3_Cr-LDH tolerated nitriles containing different electron-withdrawing and -donating substituents well. The catalyst was recycled five times and characterized by X-ray diffractometry, transmission, and scanning electron microscopy with energy-dispersive X-ray analysis, infrared, and Raman spectroscopic techniques. Based on the tests, excellent catalyst reusability results (over 80% conversion and 100% selectivity even at the fifth use) were due to the robustness of the catalytic surface. The performance of zinc-rich Zn_3_Cr-LDH catalyst rivaled that of many extreme and noticeably expensive metal-containing materials (Cu, Pd, La), and it was also highly effective for use in DMSO solvent approved for medical research. For the extension of tetrazole synthesis, four new nitriles of kynurenic acid (glutamate receptor antagonists, potential neuroprotective agents with modified blood–brain barrier permeability) were synthesized and fully characterized by nuclear magnetic resonance and mass spectroscopic analyses.

## Graphical abstract

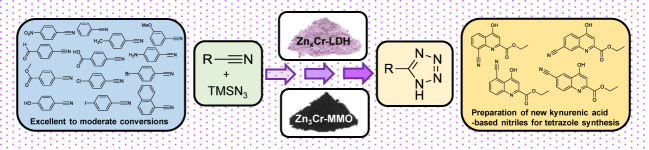

## 1 Introduction

Tetrazole derivatives represent a significant class of heterocycles, playing a crucial role in medicinal chemistry and drug design. Their importance stems not only from their bioisosterism to carboxylic acid and amide moieties but also from their notable metabolic stability.^1–5^ As of now, DrugBank records 70 drugs incorporating tetrazole substituents, with 26 of them having received FDA approval.^6^ These compounds exhibit diverse biological activities, including antihypertensive,^7^ cytostatic,^8^ antiallergic,^9^ anti-Alzheimer’s disease,^10^ antidiabetic,^11^ antiangiogenic,^12^ antibacterial,^13^ anticancer,^14^ antifungal,^15^ antimalarial,^16^ antitubercular,^17^ antiviral activities,^18^ and various other effects.^19^ Kynurenic acid (KYNA) is formed during the metabolism of tryptophan together with other endogenous mediators.^20,21^ KYNA level abnormalities can contrbute to neurodegenerative diseases,^22,23^ and one of the most prominent functions of KYNA is its antagonistic effect on glutamate receptors.^24,25^ For this reason, any type of modification (e.g., tetrazole ring formation) is a valuable and important pharmaceutical chemistry endeavor to improve the penetration of KYNA molecules across the blood–brain barrier.

Since Bladin’s pioneering preparation of tetrazole in 1885, the sustained scholarly attention to the synthesis of tetrazoles underscores the enduring significance of this ring system.^3,26,27^ Initial approaches to the preparation of tetrazoles included the diazotization of nitrogen-rich compounds, particularly imidohydrazides.^28^ Current methods for synthesizing tetrazoles often involve cycloadditions in which nitriles react with azides, typically simple inorganic (NaN_3_, HN_3_) or less explosive organic azides (trimethylsilyl, trialkyl tin, or organoaluminum azides). Use of organic variants is not only safer and less toxic, but their recovery is also easier, contributing to a more eco-friendly design of tetrazole syntheses.^29–31^ This approach has been successfully refined over the past decade through the use of highly active catalysts and substrate modifications.^32–35^

Among heterogeneous catalysts, the layered double hydroxide (LDH) clays are emerging as more favorable alternatives to address the challenges of environmental remediation.^36,37^ LDHs are a class of ionic lamellar compounds consisting of positively charged brucite-like layers with charge-compensating anions and solvated molecules in the interlayer region. The most extensively studied LDHs, with the general formula [M^2+^_1*–x*_M^3+^_*x*_(OH)_2_]­[Anion^*n*–^]_*x/n*_·*z*H_2_O, contain various divalent (M^2+^= Mg, Ca, Co, Ni, Zn) and trivalent (M^3+^ = Al, Cr, Fe) metal cations.^38^ LDHs have several advantages, including straightforward and cost-effective synthesis methods, commendable thermal stability, relatively large surface area, biocompatible nature, and low toxicity.^39,40^

These attributes have led to the widespread utilization of LDHs in various applications, such as adsorption processes,^41^ functioning as photocatalysts,^42^ participating in drug delivery systems,^43^ serving as solid base catalysts in isomerization,^44^ aldol condensations,^45,46^ alkylation processes,^47^ and in numerous other catalytic systems.^48^ Moreover, LDHs can undergo calcination at various temperatures, leading to their transformation into mixed metal oxides (MMO) through structural breakdown. This process involves the removal of interlayer anions and water molecules, resulting in partially or fully altered chemical and structural properties compared to those of the original LDHs (the accessibility of catalytically active sites can increase while their uniform arrangement at the atomic level remains largely intact).^49,50^

ZnCr-LDHs are known for their excellent catalytic activity in various reactions such as oxidation,^51^ hydrogenation,^52,53^ and even processes related to environmental remediation,^54^ making them valuable tools in the fields of synthetic chemistry and sustainable technologies.^55,56^ Although these LDHs have been widely studied as photocatalysts, interestingly, relatively little research has been done on their traditional thermocatalytic use. Over the past ∼20 years, fewer than 10 studies have been published on the function of ZnCr-LDH catalysts/catalyst supports in isomerization,^57^ coupling,^58^ hydrogenation,^59^ but mainly in oxidation reactions.^51,60–64^

Efficient separation of tetrazole products from the reaction mixture and their controllable, selective, and effective preparation methods are primary considerations from green chemistry perspectives. One of the most important research fields in this area is the synthesis and design of new adequate heterogeneous and recyclable catalysts for the production of tetrazoles.^65^ Zinc salts and other zinc-containing compounds can catalyze the preparation of tetrazoles.^66–68^ However, chromium-catalyzed methods are rarely found in the literature,^69^ and there are no reports on the catalytic production of tetrazoles by heterogeneous ZnCr-based LDHs or their calcined forms. Thus, our goal was to investigate the possibility of developing a general method using ZnCr-LDH-based catalysts that can be effectively applied to the synthesis of 5-substituted-1*H*-tetrazoles under sustainable conditions.

## 2 Experimental Section

### 2.1 Materials

Diethyl acetylenedicarboxylate, polyethylene glycol 400, 1,2-dichlorobenzene, trimethylsilyl azide, benzonitriles, and aminobenzonitriles were purchased from Sigma-Aldrich. Anhydrous Na_2_SO_4_, NaCl, NaOH, ZnCl_2_, CrCl_3_ × 6H_2_O, and dimethyl sulfoxide were received from VWR. Dichloromethane, *N,N*-dimethylformamide, ethanol, methanol, *n*-hexane, ethyl acetate, and 2-propanol were bought from Molar Chemicals. All chemicals (except trimethylsilyl azide with 94% purity) were of 98%+ purity, and no further purification was required.

### 2.2 Preparation of the Various LDH-Based Catalysts

Synthesis of Zn_*x*_Cr-LDHs was based on the coprecipitation preparation method frequently used in our laboratory,^70,71^ where an aqueous mixture of ZnCl_2_ and CrCl_3_ × 6 H_2_O starting reagents was added to a base solution to gain LDHs as precipitates. Zinc and chromium chlorides were dissolved in various molar ratios (2-6:1 Zn:Cr) in distilled water (the initial ratio was 2:1–20 cm^3^ solution of 0.3 M Zn­(II) and 0.15 M Cr­(III) ions). The obtained mixture was added dropwise to 7.1 cm^3^ of 1.5 M NaOH aqueous solution and intensively stirred for 4 days at 50 °C (to minimize the amorphous content of the forming LDH phase) under N_2_ atmosphere (to avoid the intercalation of carbonate anions stemming from atmospheric CO_2_). As-prepared LDHs were washed numerous times with distilled water, collected on 0.45 mm filters, dried at 90 °C, and stored under N_2_ (to prevent surface carbonate formation). Elemental analyses confirmed the successful synthesis of the targeted composition for all of the Zn_*x*_Cr-LDHs. Calcination (between 300 and 900 °C, preparation of MMO) of the LDHs was executed in a muffle furnace under air for 1 h applying a heating rate of 25 °C/min.

### 2.3 General Procedure for the Synthesis of 5-Substituted-1*H*-Tetrazoles

Dimethyl sulfoxide (DMSO, 4 cm^3^), the corresponding nitrile (0.4 mmol, 1 equiv), trimethylsilyl azide (TMSN_3_, 0.8 mmol, 2 equiv), and Zn_3_Cr-LDH (20 mg, corresponding to ∼10 mol % catalyst loading) were mixed in an oven-dried (at 100 °C, overnight) Schlenk tube equipped with a magnetic stir bar. Reaction mixture was stirred for 24 h at 160 °C, then cooled to room temperature, and the catalyst was filtered off. Next, 10 cm^3^ of brine solution was added (to improve the separation of the organic phase and prevent the formation of emulsions), and the resulting mixture was extracted with dichloromethane (CH_2_Cl_2_, 3 × 10 cm^3^). The combined organic layers were dried over sodium sulfate and concentrated under reduced pressure. The crude product was then analyzed by NMR spectroscopy (nuclear magnetic resonance, confirming complete DMSO removal) to determine conversion and selectivity, and then purified by column chromatography to isolate the desired products. Characterization data (^1^H NMR and ^13^C NMR) are also detailed in the Electronic Supporting Information (ESI, Graphs S1–S26) document.

### 2.4 Production of Kynurenic Acid Derivatives

To a well-stirred solution of aminobenzonitrile (1 equiv) in ethanol (EtOH) under reflux temperature (80 °C), diethyl acetylene dicarboxylate (DEAD, 1 equiv) diluted with EtOH was added dropwise. The reaction mixture was stirred for 3 h under reflux. Purification was made by column chromatography applying a mixture of *n*-hexane:EtOAc (ethyl acetate, 4:1 ratio by volume), and the compound was then checked by NMR spectroscopy. Afterward, the as-prepared enamines (**1a**–**1c**) were dissolved in 1,2-dichlorobenzene (DCB), and the mixture was stirred under reflux (∼190 °C) for 24 h. Liquids were evaporated, and the residue was purified by column chromatography using the described *n*-hexane:EtOAc mixture. Characterization data of the synthesized KYNA nitriles can be found in the ESI (Graphs S27–S34).

### 2.5 Investigation of the Catalyst Reusability

Reaction of 4-nitrobenzonitrile with TMSN_3_ was carried out several times utilizing a single portion of the catalyst. DMSO (20 cm^3^), 4-nitrobenzonitrile (2 mmol, 1 equiv), TMSN_3_ (4 mmol, 2 equiv), and Zn_3_Cr-LDH (100 mg, corresponding to ∼10 mol % catalyst loading) were placed in an oven-dried Schlenk tube equipped with a magnetic stir bar. Reaction mixture was stirred at 160 °C for 24 h. After cooling to room temperature, solid was separated by centrifugation, and liquid phase was extracted, dried, and evaporated as described above. Regained catalyst was washed with 2-propanol (4 times, solvent selection based on our previous experience,^71^ ensuring the exclusion of structural and compositional changes in catalyst particles due to washing) and dried under N_2_.

### 2.6 Apparatus for Analytical and Structural Characterization

Each catalytic test was performed at least three times before the result was reported. Kynurenic nitrile derivatives and tetrazole products were characterized by NMR spectroscopy. ^1^H NMR and ^13^C NMR spectra were recorded on a Bruker Avance NEO 500 spectrometer, in DMSO-*d*_6_ or CDCl_3_ as solvent, with tetramethylsilane as an internal standard at 500.1 and 125 MHz, respectively. Electrospray ionization mass spectroscopic (ESI-MS) analyses were performed using an LCQ Fleet Ion Trap LC/MS (Thermo Scientific) instrument with direct injection of samples diluted with acetonitrile. Crude products were purified by chromatographic methods to isolate the desired products. Catalysts were mainly characterized by scanning electron microscopy coupled with energy-dispersive X-ray spectroscopy (SEM-EDX, Hitachi S-4700 and Röntec QX2 spectrometer with Be window, 20 kV acceleration voltage), transmission electron microscopy (TEM, FEI Tecnai G220 X-Twin, 200 kV acceleration voltage), and Fourier-transform infrared (IR) spectroscopy (JASCO FT/IR-4700 using DTGS detector and ZnSe attenuated total reflectance accessory with 2 cm^–1^ optical resolution). Powder X-ray diffractometry (XRD, Rigaku Miniflex II, CoKα radiation with 2°/min scan speed and step width of 0.02° 2θ, scintillation detector operating at 30 kV and 15 mA without monochromator; for the sake of more common representation, the 2θ­(Co) values were converted to 2θ­(Cu) using the Bragg equation), N_2_ adsorption–desorption (Quantachrome Autosorb iQ instrument, degassing at 150 °C for 3 h under vacuum, Brunauer–Emmett–Teller equation to determine the specific surface areas), Raman spectroscopy (Bruker Senterra II Raman microscope, 25 mW laser power level and 532 nm excitation wavelength, 12 s exposure time, 50× magnification of the objective), and thermogravimetric-mass spectroscopy (TG-MS, Discovery TGA and Hiden Analytical HPR-20 EGA MS, 15 °C/min heating rate, 60 cm^3^/min flow of Ar containing 5% O_2_, identification of ions in the 1–300 *m*/*z* range using full scan mode, Faraday cup detector, and electron impact ionization with 70 eV energy) analyses were also applied.

## 3 Results and Discussion

### 3.1 Optimization of Tetrazole Synthesis Over Zn_*x*_Cr-Based Catalysts

Previous studies have extensively utilized trimethylsilyl azide as a valuable reagent for tetrazole synthesis due to its unique properties and versatility. Compared with other azide resources, such as sodium azide or benzyl azide, TMSN_3_ is a stable, easy-to-handle compound that can be stored and transported safely. Additionally, TMSN_3_ exhibits high reactivity, facilitating the efficient synthesis of tetrazoles with excellent yields.^72^ Therefore, this nitrogen source was chosen for the transformation of *para*-nitrobenzonitrile into tetrazole. Based on literature data,^73^ dimethyl sulfoxide was the initial choice as the solvent. The reaction mixture, containing 1 equiv of nitrile (0.1 M) and 2 equiv of TMSN_3_, was stirred at 160 °C for 24 h, and 90% conversion was found in the presence of 10 mol % Zn_3_Cr-LDH. ^1^H NMR analysis of the crude product indicated full selectivity toward the formation of 5-(4-nitrophenyl)-1*H*-tetrazole. In this reaction, the catalytic performance of Zn_3_Cr-LDH was directly compared with commercially available Zn and Cr salts, which could be considered as the starting materials of LDH synthesis (Table 1).

**Table 1:**
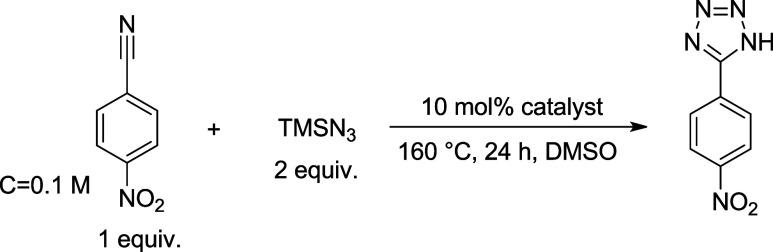
Investigation of Various Catalysts in the Synthesis of 5-(4-Nitrophenyl)-1*H*-Tetrazole from 4-Nitrobenzonitrile and TMSN_3_ as the Starting Materials^a^

#	Catalyst	Conv. (%)^b^
1	Without catalyst	-
2	ZnCl_2_	24 ± 1.63
3	CrCl_3_ × 6H_2_O	17 ± 5.35
4	Zn(OH)_2_	61 ± 5.00
5	Cr(OH)_3_	21 ± 0.81
6	Zn_3_Cr-LDH	90 ± 1.63
7	3 Zn(OH)_2_ + Cr(OH)_3_	54 ± 2.18
8	3 ZnCl_2_ + CrCl_3_ × 6H_2_O	2 ± 0.46

a Reaction conditions: 1 equiv. (0.1 M) nitrile, 2 equiv. TMSN_3_, 10 mol % catalyst, solvent: DMSO, 160 °C, 24 h reaction time.

b Determined by ^1^H NMR analysis of the crude products (full selectivity for all cases).

The selectivity was 100% in all cases, and product formation was not observed in a test reaction without a catalyst. Both salts were highly soluble in DMSO but proved less effective than LDH. Namely, in the presence of anhydrous ZnCl_2_, the conversion was 24%, while a conversion of a mere 17% was achieved with CrCl_3_ × 6H_2_O. The individual solid hydroxide forms of Zn and Cr metals had higher activity than the dissolved chloride salts (61 and 21%, respectively). However, their performance still lagged significantly behind that of Zn_3_Cr-LDH (90%), which may indicate a synergistic impact between the two metals in the mechanism of LDH catalysis. This effect could only occur to a significantly lesser extent when the physical mixture of the two metal hydroxides was used, which may explain the lower conversion rate of 54% compared to that observed for LDH, but better than the performance obtained with the metal hydroxides used separately. This suggests that the atomic-level proximity provided by the LDH phase and the uniform distribution of Zn and Cr metal ions in the catalyst structure may have been crucial parameters. In the case of Zn_3_Cr-LDH sample, the presence and homogeneity of the Zn, Cr, and Cl elements were visualized by EDX spectra and spatially resolved elemental maps (Figure S1, S signs to the data in the ESI). Interestingly, when the two metal salts were present simultaneously in the DMSO solution, tetrazole synthesis practically did not occur. In this case, it is conceivable that the formation of a common complex between the Zn­(II) and Cr­(III) ions and the *para*-nitrobenzonitrile reagent may have prevented the generation of the nitrogen ring.

After these promising preliminary results, the effects of general reaction conditions were examined (Figure 1, Table S1–S3). Typically, the reaction is conducted at elevated temperatures, necessitating the use of solvents with higher boiling points and the ability to withstand such conditions. *N,N*-dimethylformamide (DMF) and DMSO emerge as the prevalent choices for tetrazole synthesis, owing to their high boiling points and compatibility with the reaction requirements. There are significantly fewer examples of other solvents being used in the literature.^73^ In most tetrazole syntheses involving TMSN_3_, we found that DMF, toluene, and ethers were used most frequently. Of these solvents, DMF can be considered the most environmentally friendly due to the harmful physiological effects of toluene and the dangerous tendency of ethers to form unstable peroxides. Interestingly, in this reaction, DMF had produced only a trace amount of the desired compound, whereas DMSO promoted a very high yield. The layered structure of LDHs can be disrupted when dissolved in an organic solvent, forming a colloidal suspension of unique layers or irregular subunits of a few lamellae. This is the so-called delamination process, which is often used to increase the catalytically active surface area of the LDHs. In DMF, there are numerous examples of delamination in different types of LDHs (MgAl-, CoAl-, NiAl-, ZnAl-LDH, etc.),^33,68^ and partial delamination was also observed in DMSO.^74,75^ Although we did not find any literature data on the degree of delamination in different media for ZnCr-LDH, it is easy to imagine that the more pronounced delamination generally observed in DMF solvent (complete disintegration of the layered structure and separation of metal hydroxides) was a disadvantage during tetrazole synthesis, thus explaining the better conversion results obtained in DMSO. In addition, the preference for DMSO as the reaction medium was further supported by Pfizer’s solvent selection guide for medicinal chemistry, which designates it as a usable solvent while categorizing most amide solvents as undesirable.^76^

**Figure 1:**
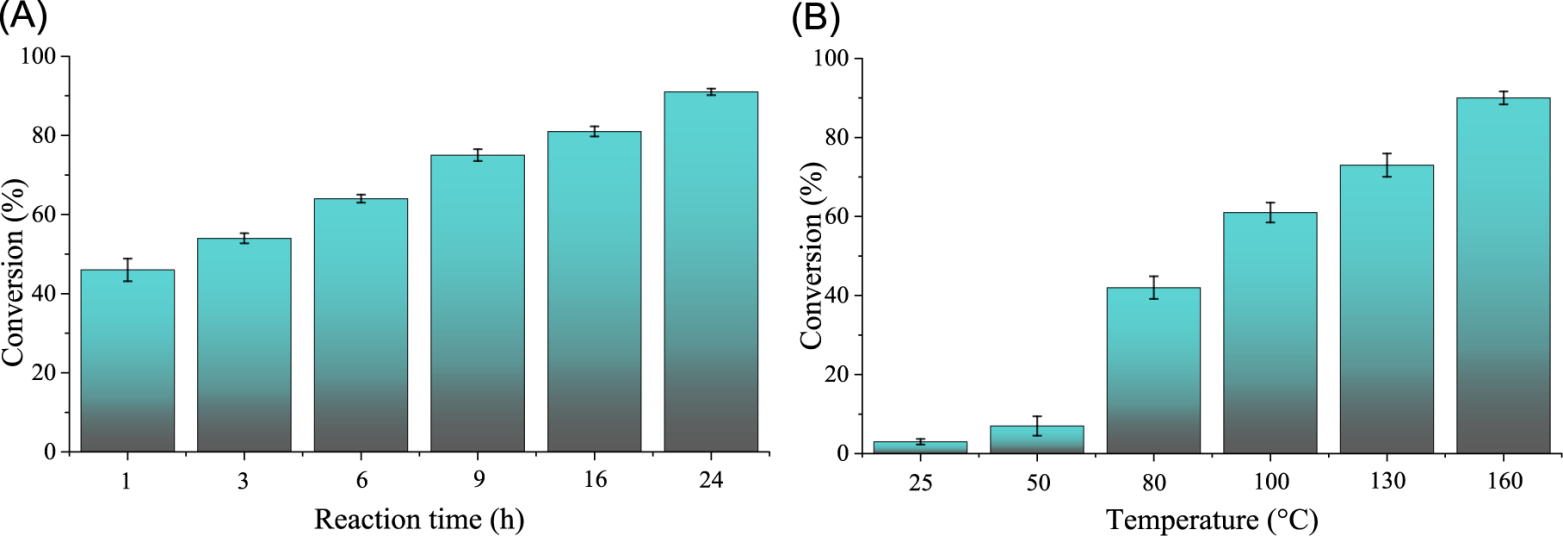
Investigation of reaction time (A, reaction conditions: 1 equiv. nitrile (*c* = 0.1 M), 2 equiv. TMSN_3_, 10 mol % catalyst, DMSO solvent, 160 °C) and temperature (B, reaction conditions: 1 equiv. nitrile (*c* = 0.1 M), 2 equiv. TMSN_3_, 10 mol % LDH, DMSO solvent, 24 h) in the synthesis of 5-(4-nitrophenyl)-1*H*-tetrazole from 4-nitrobenzonitrile and TMSN_3_ (full selectivity for all cases).

It is important to note that the use of DMSO requires the use of dichloromethane as an extractant, which is less desirable for environmental reasons. However, there are only a few solvents that cannot be mixed with DMSO; an alternative to CH_2_Cl_2_ is heptane, which has a low boiling point and is considered a suitable solvent according to Pfizer’s solvent selection guide. The utilization of PEG-400 (polyethylene 400), a high-boiling point solvent and environmentally friendly alternative to DMSO, was also investigated at 160 and 120 °C, as it is widely used even for low-temperature tetrazole syntheses.^77^ However, the results were similar to those observed with DMF, with only a small amount of the desired product being produced. Based on the literature, the disintegration of Zn_3_Cr-LDH particles and modification of their surface (PEGylation),^78,79^ similar to DMF, is also conceivable here, which confirmed the possible negative effects of LDH delamination/exfoliation in tetrazole synthesis.

Effects of substrate concentration were explored within the ranges of 0.1 and 0.25 M (Table S1); increasing concentration only slowly reduced the degree of conversion (90% was achieved at 0.1 M, while 68% was achieved at 0.25 M). This suggests that the catalyst loading used (10 mol %) represented a remarkable amount of catalytically active centers. Thus, even with a significant increase in substrate quantity, the catalytic conversions per unit time decreased only slightly. Therefore, this catalyst may also have great potential in scaling up tests; however, this is beyond the scope of this work. With regard to reaction time, conversion increased gradually, and 24 h was required to achieve 90% transformation with a selectivity of 100% (Figure 1A). A significant amount of 5-(4-nitrophenyl)-1*H*-tetrazole was already detectable in the early stages, reaching 42% after 1 h and around 75% after 9 h, which shows that it is not necessary to plan for 24 h reactions in order to achieve economical production. As the next step, the effect of temperature was investigated (Figure 1B); as expected, elevating the temperature significantly increased the reaction rate, achieving 90% conversion of 5-(4-nitrophenyl)-1*H*-tetrazole at 160 °C after 24 h, while the selectivity remained 100% at all stages. We attempted to reduce the excess of TMSN_3_ applied, but 2 equiv were required to complete the reaction. Lower amounts resulted in a slight reduction in conversion (78%), whereas higher amounts did not give any significant increase in conversion (Table S2, entries 1–3). Optimal catalyst loading was found to be ∼10 mol %, as lower catalyst amounts resulted in a decrease in conversion, but to a lesser extent than expected. Similar to what was observed when increasing the substrate concentration, this may again indicate the excellent potential of catalysts in scale-up tests. Higher catalyst amounts, in turn, barely increased the conversion (Table S2, entries 4–8).

Finally, we systematically investigated the effect of different Zn:Cr molar ratios on the catalytic performance of LDHs. It is important to note that LDH phases with a relative zinc content of less than 2:1 Zn:Cr molar ratio cannot be produced purely; consequently, catalysts with such compositions were not investigated. XRD curves (Figure 2A; raw files can be found at the end of Electronic Supporting Information document) of the solids showed typical reflections of LDH phases for all Zn:Cr molar ratios between 2:1 and 4:1,^71,80^ with no signs of byproduct formation and relatively consistent average crystallite sizes (5–7 nm, calculated from the full width at half-maximum of the first reflections using Gaussian distribution and Scherrer equation with a shape factor of 0.9). The use of higher initial Zn:Cr molar ratios (5:1 and 6:1) resulted in minimal zinc chloride hydroxide monohydrate formation (identified by International Diffraction Database card number 77-2311). As expected from the performance of the separate Zn­(OH)_2_ and Cr­(OH)_3_ solids (Table 1), increasing the Zn content of the catalyst improved the conversion from 81% (2:1) to 90% (for 3:1 Zn:Cr molar ratio) (Table S3, entries 1–5). However, further increases only helped tetrazole synthesis to a small extent, with conversion values enhancing to 95% at a molar ratio of 6:1. This shows that although only a minute amount of basic zinc salt byproduct was produced, its presence did not interfere with the catalyst; in fact, it is conceivable that this phase also played an active role.

**Figure 2:**
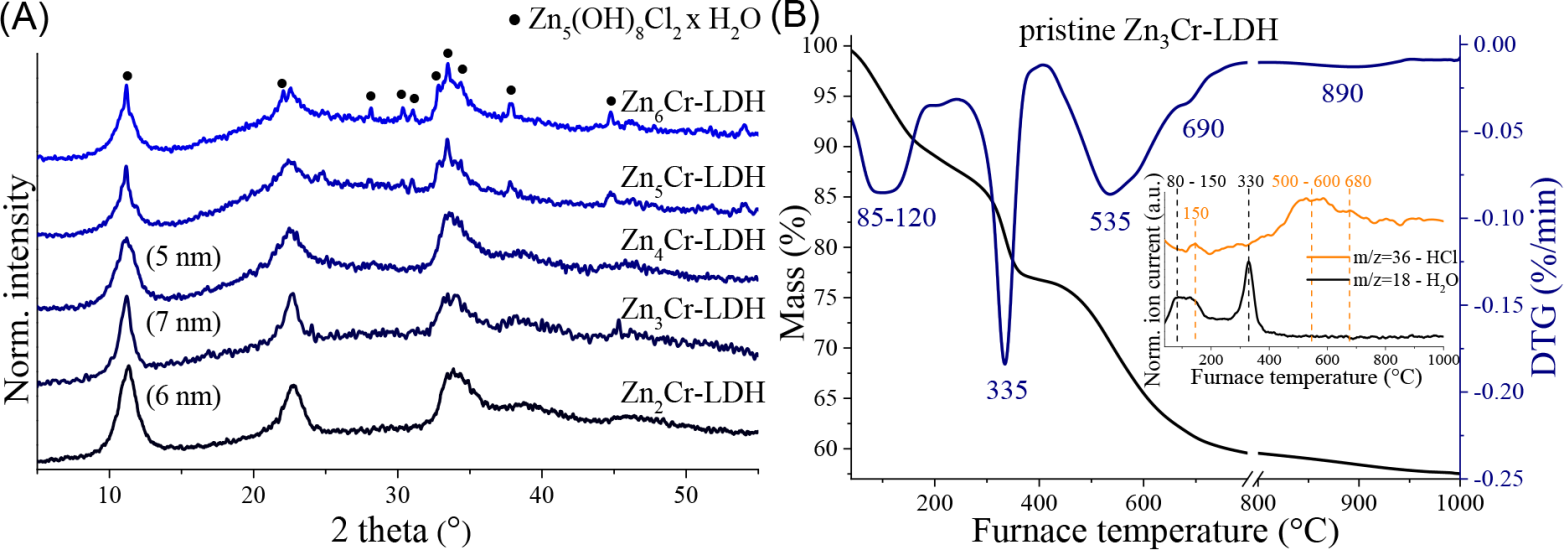
XRD curves (A) of the Zn*_x_* Cr-LDHs prepared with various Zn:Cr molar ratios and thermogravimetric (B), derivative thermogravimetric and evolved gas analyses of the starting Zn_3_Cr-LDH catalysts.

We considered it worthwhile to examine the effect of calcination on LDHs as well, since it is well-known in the literature on LDHs that calcination can have a favorable effect in many catalytic respects. While the layered structure is partially or completely lost, and the structural/interlayer water and anion molecules are removed, active metal oxide composite products with relatively high porosity and specific surface area, as well as small crystallites with high thermal stability can be formed.^71,77^ However, the heat treatments of Zn_3_Cr-LDHs between 300 and 900 °C did not aid conversion (Table S3, entries 6–11), and calcination at 600 °C gave the best results (76% conversion and 100% selectivity). This clearly showed that, in addition to Lewis basic units (O^2–^ ions, produced by heat treatment), Brönsted base properties (OH^–^ moieties, abundant in the starting LDH form) also play a significant role in the reaction mechanism.

This was confirmed by thermogravimetric analysis of the Zn_3_Cr catalyst, which showed the typical thermal behavior of the LDH framework, with only endothermic processes (Figure 2B). Until 300 °C, a wide and significant mass loss (∼15%) was observed on the surface due to the loss of physically adsorbed water and, most likely, surface OH groups, which are crucial for catalysis. This may explain the conversion rate dropping from 90% to 66% as a result of the 300 °C heat treatment (Table S3), despite the expected increase in specific surface area (5 m^2^/g for the pristine LDH and 33 m^2^/g for MMO calcined at 300 °C). It is worth noting that at around 150 °C, there is a weak signal indicating the departure of HCl molecules with a value of 36 *m*/*z*, which can be linked to the decomposition of surface chloride and OH^–^ ions. In the next step, the evaporation of water molecules (only 18 *m*/*z* MS signals) from the interlayer space was recorded with a maximum mass loss at 335 °C, and this had no significant effect on the change in activity. The maximum tetrazole synthesis observed during calcination at 600 °C is presumably due to the fact that, despite the increase in specific surface area (compared to the area of pristine LDH, 21 m^2^/g for MMO calcined at 600 °C), the structural OH groups in the layers and the interlayer chloride ions (with mass losses around 535, 690, and 890 °C) could only be partially removed. By further increasing the heat treatment temperature to 700 °C, these processes progressed significantly, and at 900 °C, they were complete, resulting in a further significant reduction in conversion values to ∼60% (Table S3), while the specific surface area (9 m^2^/g for MMO calcined at 900 °C) did not decrease below the value of the pristine LDH. All this suggests that during catalysis, in addition to the Lewis acid centers of metal ions, the layered Lewis base O^2–^ ions and Brönsted base OH^–^ units, the Lewis basic chloride anions found in the structure may also have participated to a relatively greater extent. However, determining their direct catalytic contribution requires further investigation in the future.

### 3.2 Extension of the Tetrazole Synthesis and Study of Reaction Mechanism

After determining the optimal conditions for the synthesis of 5-(4-nitrophenyl)-1*H*-tetrazole model compound (10 mol % Zn_3_Cr-LDH catalyst loading, DMSO solvent, 0.1 M substrate concentration, 160 °C, and 24-h reaction time), the scope and applicability of the reaction were investigated (Table 2, entries 1–13, for all cases, full selectivity). Aromatic nitriles containing electron-withdrawing groups (p-NO_2_, p-COOH, and p-CHO) gave tetrazole products in excellent yields, except for methyl 4-cyanobenzoate, which gave moderate conversion (Table 2, entries 1–3, 8). On the other hand, aromatic nitriles bearing electron-donating groups (*p*-NH_2_, *p*-OH) also demonstrated outstanding tetrazole production, whereas moderate conversions were obtained with 4-methylbenzonitrile and unsubstituted benzonitrile (Table 2, entries 4–7). For halogenated nitriles (*p*-Cl, *p*-Br, and *p*-I derivatives), *m*-methoxybenzonitrile, and naphthalene-1-carbonitrile, low conversions were measured (Table 2, entries 9–13).

**Table 2:**
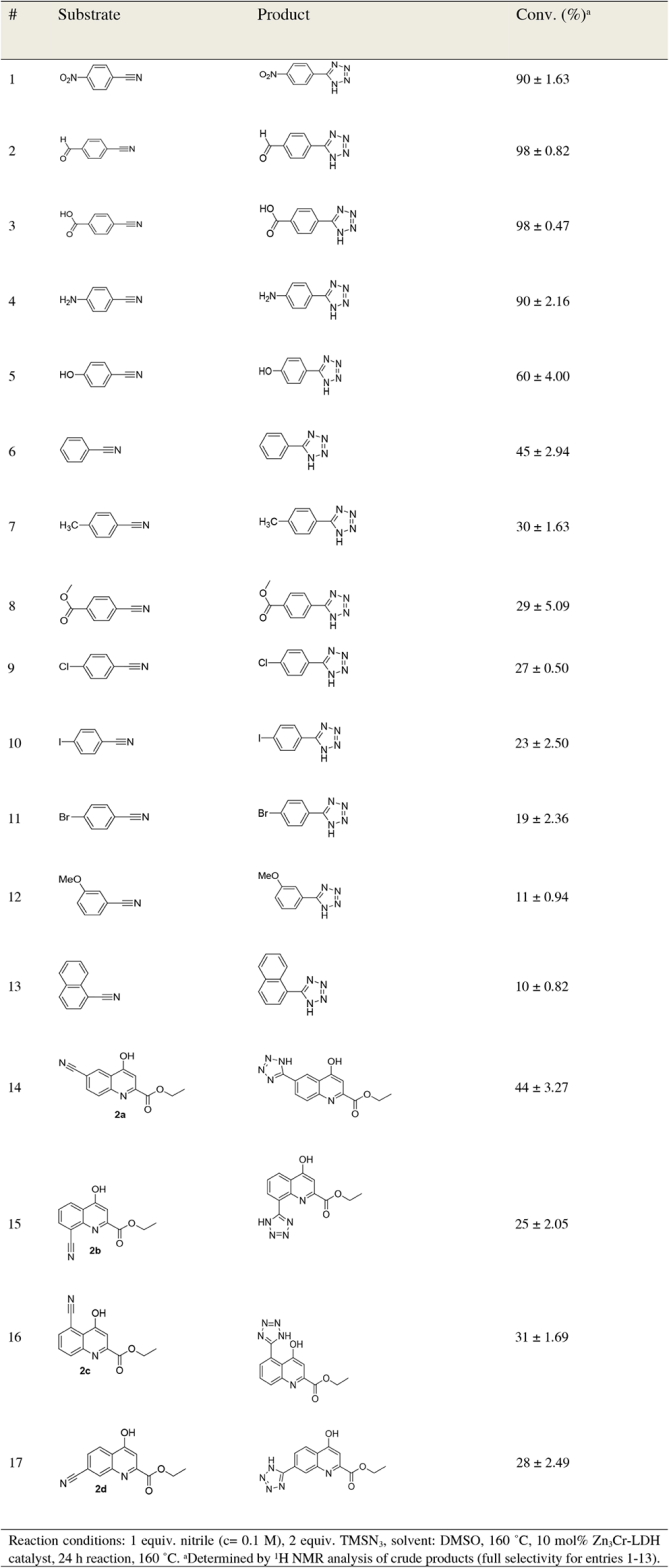
Exploring the Zn_3_Cr-LDH-Catalyzed Synthesis of 1*H*-Tetrazoles from Different Nitriles and TMSN_3_

Compared to the unsubstituted benzonitrile, tetrazole synthesis was less favorable in many cases (entries 5–12), highlighting the conversion of only about 10% measured for *m*-methoxybenzonitrile. This focuses on the *para*-directing relevance of substituents in the reaction but does not explain why the syntheses proceeded equally well with strongly electron-withdrawing and electron-donating groups. However, the effect of various components of the catalyst may give some indication of this. On the one hand, the formation of the strongly nucleophilic azide anion derived from the TMSN_3_ reagent could have occurred not only through thermal decomposition but also from hydrolysis, reacting with the water content of LDH (and to some extent DMSO). Furthermore, it is also conceivable that the azides could have been released during complex formation with the metal content of the catalyst.^81,82^ Their nucleophilicity could have been further increased by the catalyst’s Lewis basic sites (O^2–^ and Cl^–^ ions). Similarly, the Lewis acidic centers could further enhance the electron deficiency of the carbon atom of the nitrile group, thereby increasing the reactivity toward the nucleophilic azide reagent. Its influence could be so great that it completely counteracts the effect of the electron-donating groups of the benzene ring. Strong coordination between the nitrile substrate and Zn­(II) or Ag­(I) ions has also been demonstrated previously.^83,84^ Finally, except for methyl 4-cyanobenzoate, tetrazole preparation proceeded excellently or well in all cases (entries 1–5), where the substituents of benzonitriles were capable of forming hydrogen bond interactions with the OH units of LDH. Due to the flat structure of the benzene ring, it can be easily assumed that the interaction between the nitrile/substituent groups and the LDH acid–base components could have developed simultaneously on the catalytic surface. A schematic representation of these is shown in Figure 3. However, this close benzonitrile–LDH coordination could occur only to a limited extent due to the steric hindrance of the methyl group in the case of methyl 4-cyanobenzoate.

**Figure 3:**
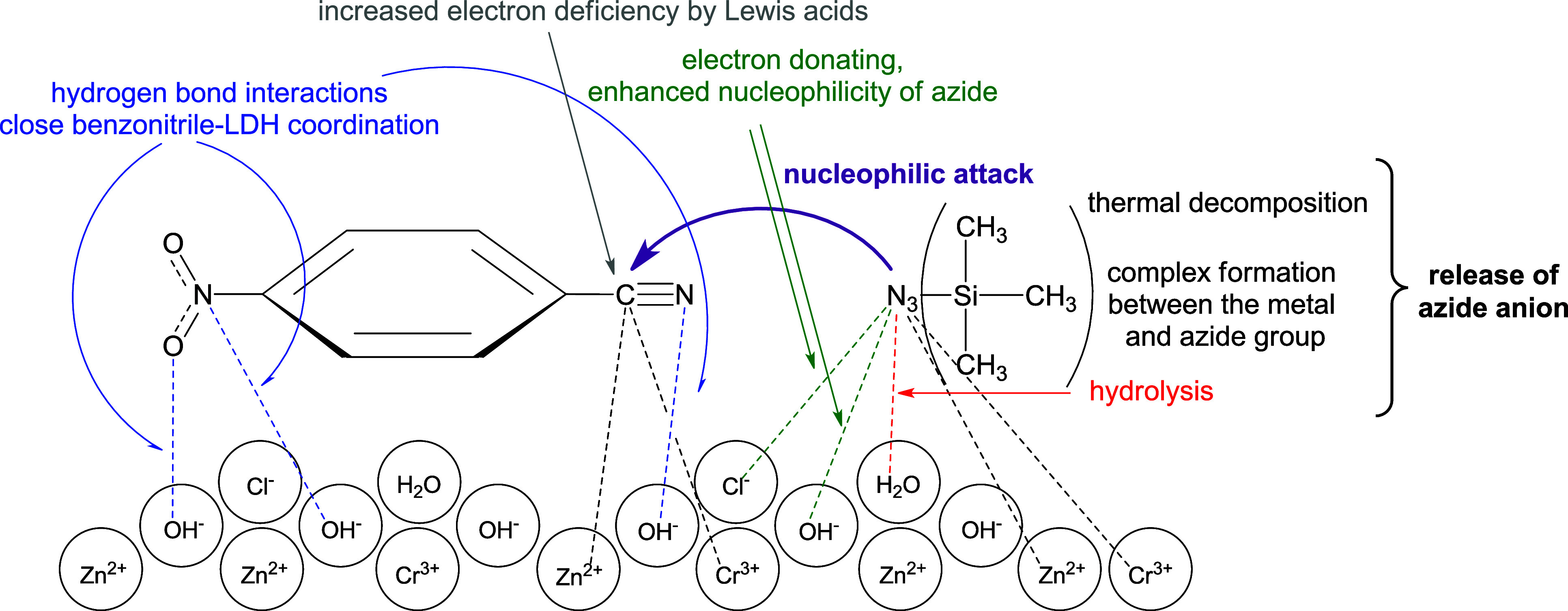
Infographic summary of possible interactions in the ZnCr-LDH/benzonitriles/TMSN_3_ system.

Hammett plot analysis displayed a negative slope (−0.59, Figure S2), but with a very weak coefficient of determination (0.55), even when excluding the results from a few electron-withdrawing groups (*p*-NO_2_, *p*-COOH, and *p*-CHO). A negative slope could indicate that a positively charged transition state is involved in the cycloaddition. This charge can be compensated and stabilized by surface Lewis base O^2–^ and Cl^–^ ions and Brønsted base OH^–^ units, which may reinforce the importance of these units in the outlined reaction mechanism. The low linear correlation indicates that caution should be exercised in interpreting the results, but it clearly highlights the decisive nature of the LDH–benzonitrile interactions. These ideas are also consistent with the explanation of the changing activity of catalysts under the influence of heat treatment, but their confirmation will definitely require further investigations (computational analyses, *in situ* reaction monitoring) in the future.

### 3.3 Preparation and Characterization of New KYNA Nitriles and Tetrazole Synthesis Attempts

For further testing of the scope and limitations of the nitrile–tetrazole transformation, new KYNA nitriles have been designed and synthesized as **(2a–2d)** serving as unique, representative nitriles. Steric hindrance to tetrazole synthesis was to be expected, as seen with naphthalene-1-carbonitrile (only 10% conversion). On the other hand, due to the nearly planar structure of the molecules and their ability to form numerous hydrogen bonds with the catalyst surface, tetrazole generation seemed feasible. However, by using the optimized conditions, the synthesis led to the formation of multicomponent reaction mixtures, and the desired KYNA tetrazoles could not be isolated (Table 2, entries 14–17). The unsuccessful tetrazole formation starting from KYNA nitrile precursors can be explained by different reasons. One possibility is that due to the strong complexation ability of the substrate, it is hypothesized that the precursors form strong complexes with the ionic content of the catalyst, thus inhibiting its catalytic activity. Moreover, KYNA molecules in solution can be present either in enolic- or oxo-form^85^ (Figure S3) and under the reaction conditions (relatively high temperature and long reaction time) their ester groups may even have hydrolyzed. These possibilities might also influence the complexation ability of substrates **2a**–**2d** with the catalyst.

Results of the synthesis extension clearly showed that due to the complexity of the LDH–benzonitriles–TMSN_3_ system (and because there are very few examples of this in the literature), further studies are needed to gain a more accurate understanding of the catalytic mechanism and the transformation of KYNA nitriles. Anyway, it is important to note that the **2a–2d** KYNA-based nitriles produced are considered new materials; to the best of our knowledge, there are no published data on them. Therefore, the preparation procedure (Figure 4) and the full NMR and MS characterization data (Table 3) of these molecules were included in this research, and NMR spectra can be found in the ESI document (Graphs S27–S34). Modifying the kynurenic acid skeleton in ring B by introducing a nitrile group may improve its neuroprotective effect and also have a beneficial effect on blood–brain barrier permeability. These biological studies will certainly be worth conducting in the future, but this goes beyond the scope of the present work.

**Figure 4:**
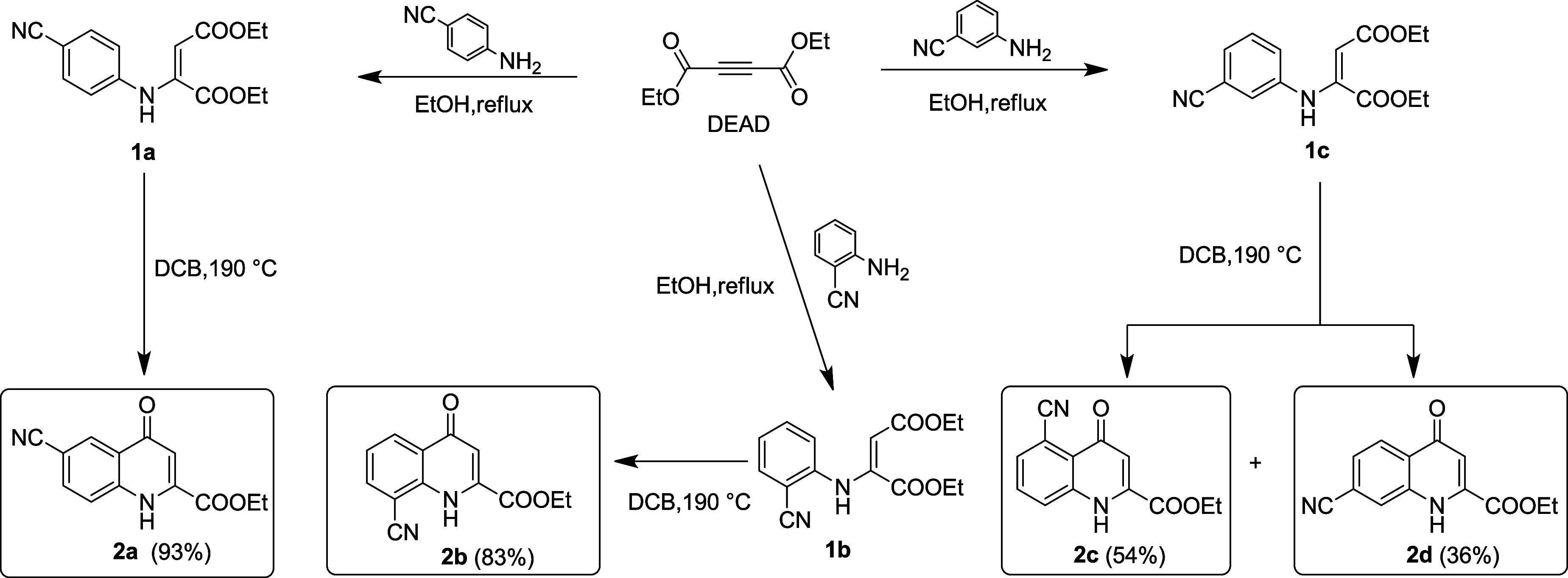
Procedure for the synthesis of ethyl 5-, 6-, 7-, 8-cyano-substituted kynurenic acid derivatives (2a–2d).

**Table 3:**
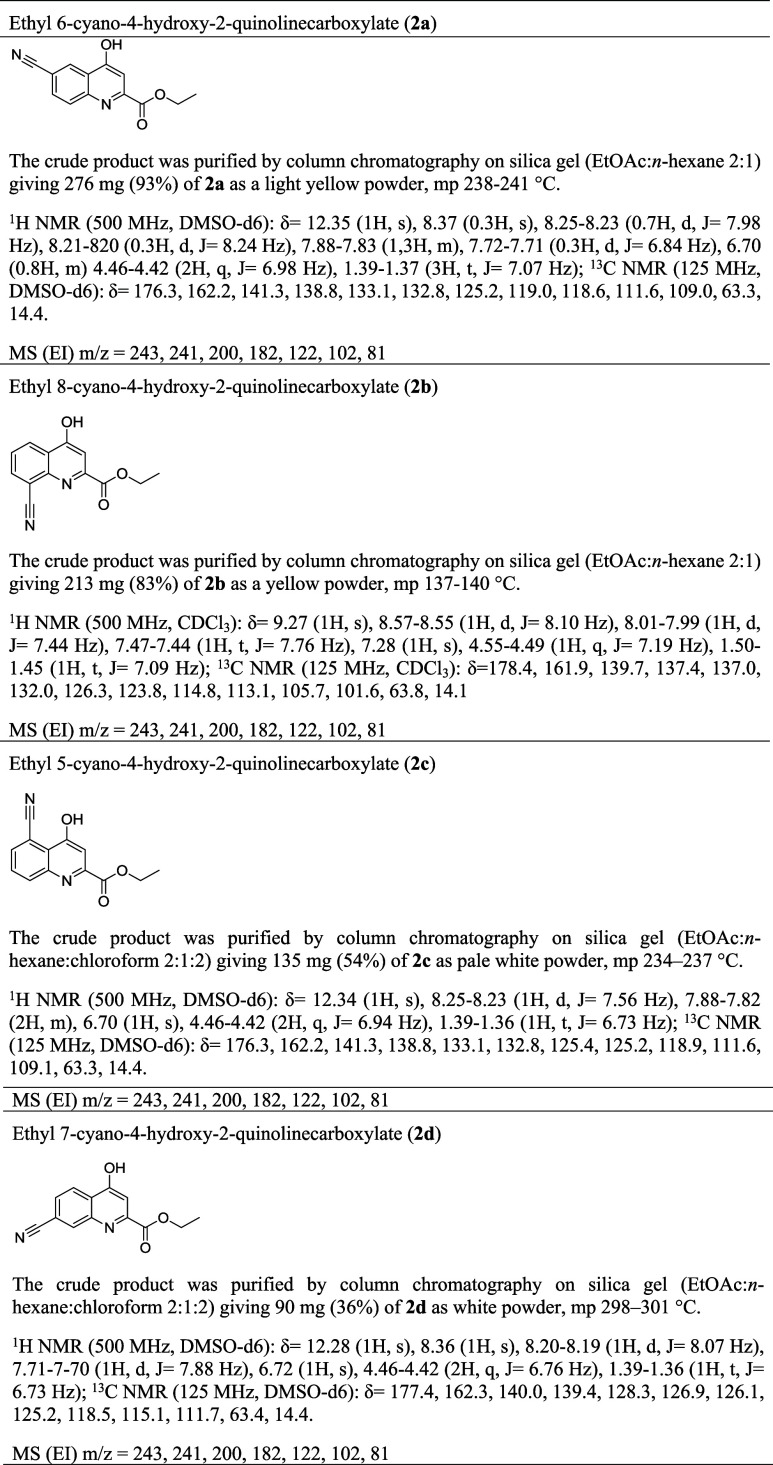
Purification and Characterization Data (^1^H NMR,^13^C NMR, MS) for New KYNA Nitriles

### 3.4 Reusability and Comparative Works of the Zn_3_Cr-LDH Catalyst

In order to evaluate the sustainable property of Zn_3_Cr-LDH, the synthesis of 5-(4-nitrophenyl)-1*H*-tetrazole was carried out several times under the optimized reaction conditions (Figure 5A). Activity of the catalyst decreased slightly during the reusability process, but the conversion remained between 90 and 80%, and the selectivity was 100% during the experiments. XRD measurements showed no significant structural changes (not shown) after the fifth use compared to the initial Zn_3_Cr-LDH form; no byproduct generation was observed; and only a slight baseline rise and broadening of the reflections were recorded, indicating minimal damage to the layered framework. IR spectroscopy analysis showed the typical vibration patterns of the LDHs for the used catalyst, but several new peaks were also detected (Figure 5B). Belonged to the LDH phase were peaks of OH units coupled by hydrogen bridges (around 3370 and 3280 1/cm), interlayer water bending vibrations (1650–1620 1/cm), and metal–oxygen lattice bands (560 1/cm).^71^ Signs of possible organic contaminants of the catalyst: asymmetric and symmetric N–O stretching modes (1560/1550 and 1410/1350 1/cm),^86^ C–H bending (1410 1/cm), S=O (1040 1/cm), and C–S–C (690 1/cm) stretching vibrations.^87^ The presence of Zn and Cr metals was detectable in the reaction solution, generally in minimal amounts, but this depended greatly on the reaction parameters. Since the Zn:Cr ratio was the same as that found in the starting catalyst, the release of metals into the solution was probably due to the delaminating effect of DMSO (the extent of which could be influenced by the actual reaction parameters). Elemental analysis of the spent catalyst did not show the leaching of ions from the layers or the interlayer galleries, but significant and uniform sulfur accumulation was observed (average Zn:Cr:Cl:S molar ratio was 3:1:1:0.2, Figure S4, with sulfur in negligible amounts in pure catalysts, Figure S1). This and IR measurements showed that a significant amount of DMSO solvent could remain in the samples. Based on the high intensity of IR vibrations associated with the organic materials (compared to the peaks of the LDH phase), a significantly faster deactivation would have been expected, so further studies focusing more on the surface were performed.

**Figure 5:**
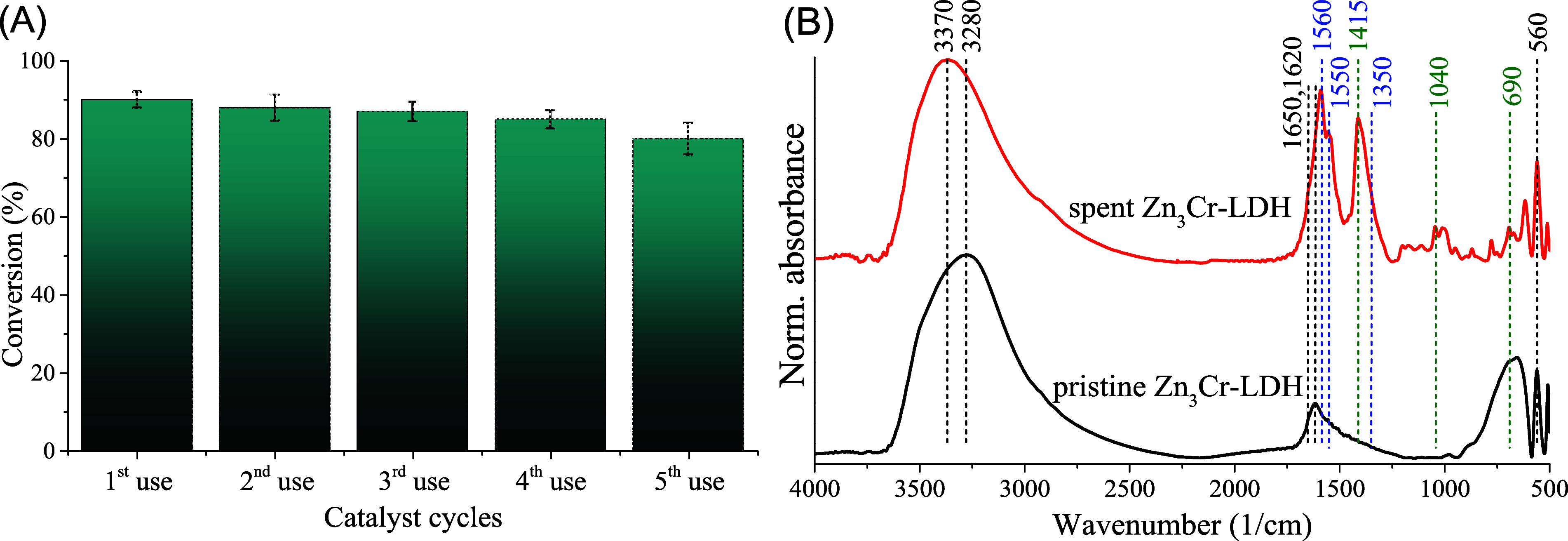
Testing the reusability (A) of the Zn_3_Cr-LDH catalyst in the synthesis of 5-(4-nitrophenyl)-1*H*-tetrazole using 4-nitrobenzonitrile and TMSN_3_ as starting materials. Selectivity was 100% in all of the reactions. Reaction conditions: 1 equiv. nitrile (*c* = 0.1 M), 2 equiv. TMSN_3_, 10 mol % catalyst, DMSO as solvent, 24 h, 160 °C, and infrared spectra (B) of the starting and spent/used Zn_3_Cr-LDH catalysts.

XRD-predicted mild structural and textural changes were clearly visible in scanning electron microscopy images of the used catalyst particles (Figure 6A, A1, B, B1, and S5). Pristine solid showed the lamellar arrangement typical of LDHs, with loose packing of particles averaging less than 1–2 μm. During use, the morphology of particles changed considerably. Specifically, the sharp contours of particles disappeared, their shape became more amorphous, and through a high degree of aggregation, the size of particles increased noticeably. Interestingly, the Raman spectroscopy did not reveal any organic contamination (Figure 6C); only the vibrations of the layer components (at 145, 435, and 625 1/cm)^88^ were observed for intact and spent catalysts. Minimal change was only observed for Cr–O–Cr linkages, which may indicate that a small portion of the surface Cr­(III) units may have oxidized to Cr­(VI), which is very common in chromium-containing LDHs.^89–91^ According to our previous study with Ca_2_Cr-LDHs,^92^ a change in the initial >90 at% surface Cr­(III) content (<10 at% for Cr­(VI)) by 2–4 at% showed more significant changes in the Raman peaks than in the case of Zn_3_Cr-LDH used in the present study. This presumably indicates the formation of extremely small amounts of Cr­(VI). Moreover, this change was measurable only in the upper few nanometers of the surface layer, while the oxidation tendency in the bulk phase may have been even lower. Based on this, it can be assumed that the appearance of highly toxic Cr­(VI) during the reaction poses a minimal risk from a safety/environmental perspective, but it is important to take this into account.

**Figure 6:**
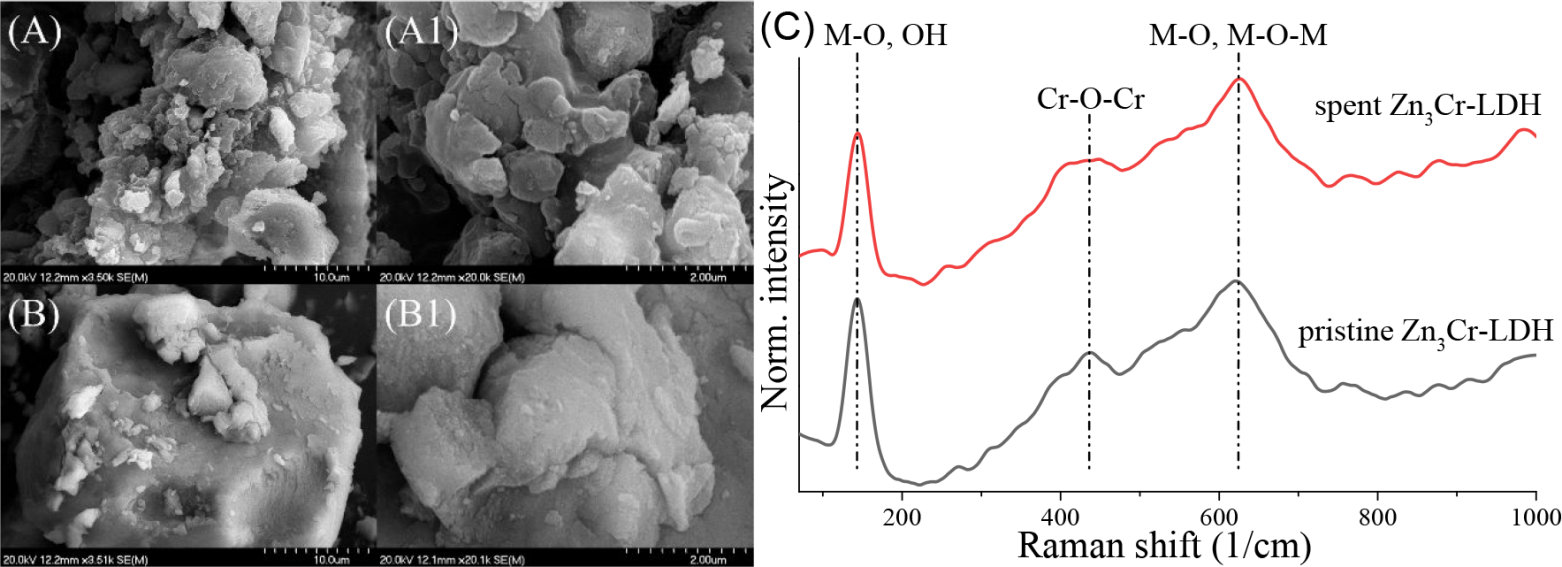
SEM photographs of the starting (A, A1) and used (B, B1) Zn_3_Cr-LDH catalyst with varied magnifications (scale bar of 10 μm for A, B and 2 μm for A1, B1 images), (C) Raman spectra of the starting and used Zn_3_Cr-LDH catalysts.

Based on these results, the slow deactivation of the catalyst is likely due to the observed aggregation processes and slight modifications in the structure and surface. While the limited removability of the DMSO medium and reaction products, not from the catalyst surface but from the voids between the LDH particles (taking into account that Raman microscopic analysis certainly provides less information about the bulk phase than IR measurement), did not significantly affect the catalytic performance. Furthermore, the complete absence of DMSO vibrations in the Raman spectra suggests that the accumulation of sulfur in the catalysts is probably not due to the strong surface adsorption of DMSO, but rather the result of incomplete washing (explaining its catalytically inactive effect). Finally, TEM studies also visualized a significant degree of aggregation. Transmission imaging made it clear that the size of the aggregates had increased significantly compared to the initial LDH sizes, reaching micron ranges (Figure S6 and S7). High-resolution photos confirmed that the specific lamellar arrangement of LDH particles changed only slightly during catalysis (Figure 7). However, images of the spent catalyst show significant damage to hexagonal particles (characteristic of LDH crystallization) in several places. It is conceivable that this modification is the result of partial dehydration and oxidation of Cr–OH units; however, the extent of this is presumably negligible, as IR measurements showed no substantial change between 2500 and 3700 1/cm.

**Figure 7:**
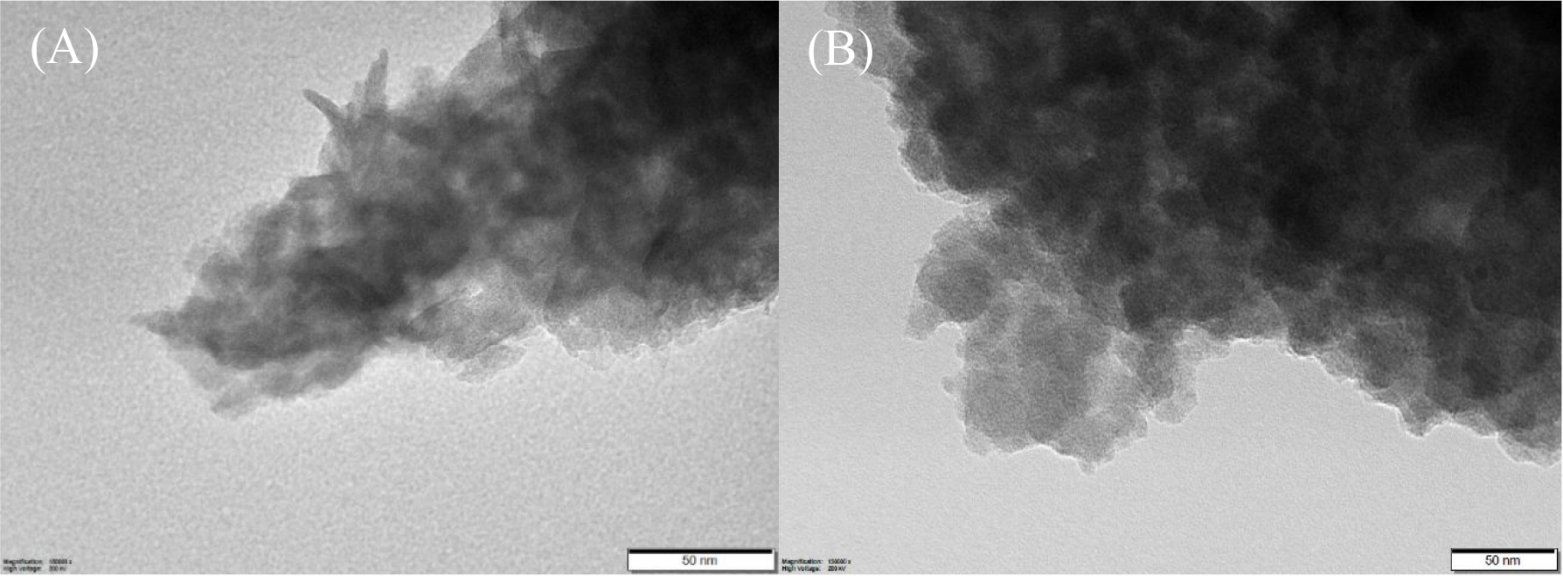
High-resolution TEM images of the pristine (A) and used (B) Zn_3_Cr-LDH catalysts (scale bar of 50 nm).

Comparative studies of catalysts are significantly complicated by the fact that differently substituted benzonitrile starting reagents are used in the literature. For a more accurate assessment, we have endeavored to present only those works in which research also included the production of variously substituted tetrazole products. Thus, the parameters presented in Table 4 often represent wide ranges based on the synthesis extension tests, which were generally performed under the preoptimized reaction conditions. Utilization of NaN_3_ is still extremely widespread (our comparison includes the most recent results from the last 10 years), despite the fact that its use often releases toxic and explosive HN_3_ gas and that for most metal cations, salt formation sensitive to friction, shock, and static electricity must be taken into account. Therefore, great caution must be exercised in laboratory and industrial use to avoid serious injury.^93^ This also applies to organic azides, which are generally flammable, react with water to form HN_3_ gas, and are more easily absorbed through the skin (especially when dissolved in DMSO) due to their hydrophobic nature, which increases the risk of poisoning. However, with appropriate care, the application of TMSN_3_ poses a much lower explosion hazard and, with its higher solubility in organic solvents, it can be used safely even under the more intense flow conditions.^94,95^ Nevertheless, there are still surprisingly few examples of its use in tetrazole syntheses; thus, we extended our research to the last 30 years.

**Table 4:** Comparative Summary of the Catalytic Syntheses of Differently Substituted Tetrazoles

Catalyst	Nitrogen source	Reaction time (h)	Temperature (°C)	Solvent	Yields (%)	Ref
Zn_3_Cr-LDH	TMSN_3_	24	160	DMSO	10–98	This work
Me_2_/Bu_2_-SnO	TMSN_3_	24–72	93–110	Toluene	60–98	^81^
Me_3_Al	TMSN_3_	72	80	Toluene	10–98	^96^
Tetrabutylammonium fluoride	TMSN_3_	1–48	85–120	Solventless	80–97	^97^
Cu_2_O	TMSN_3_	12–24	80–120	MeOH/DMF	36–96	^98^
Bu_3_SnOMe	TMSN_3_	4	140	Bu_2_O	33–99	^82^
La(OTf)_3_-SiO_2_	NaN_3_	7	100	MeOH/DMF	73–88	^99^
Tetrabutylammonium hydrogen sulfate	NaN_3_	3–20	85–100	Toluene or H_2_O	53–98	^100^
Epichlorohydrin-SiO_2_	NaN_3_	1–7	130	DMSO	75–96	^101^
Pd@MCM-41 silica	NaN_3_	2.5–3	110	PEG-400	90–96	^102^
Pd-l-lysine@Fe_3_O_4_	NaN_3_	0.5–2.5	100	H_2_O	70–99	^103^
Al_2_O_3_ nanoparticles	NaN_3_	1–3	140	DMSO	80–99	^104^
ZnFe_2_O_4_@SiO_2_ nanoparticles	NaN_3_	1–4	120	DMF	85–99	^105^

In the case of the TMSN_3_ azide source (Table 4), most of the catalysts found were organic compounds, and their operation was dominated by homogeneous characteristics, which significantly limited their reusability. The only exception to this was the Cu_2_O-based catalyst, in which case similar reaction times were reported as in our work. Reaction temperature was lower when using the less environmentally friendly MeOH/DMF solvent; meanwhile, the Cu_2_O catalysts showed only 30% yield in DMSO.^98^ It is important to note that the use of Zn_3_Cr-LDH required a slightly higher reaction temperature on average compared to other catalysts but resulted in a shorter reaction time. However, these two parameters proved to be mutually variable; at 130 °C for 48 h, we were able to achieve a similar yield of 5-(4-nitrophenyl)-1*H*-tetrazole as at 160 °C for 24 h. Using NaN_3_, shorter reaction times were generally reported at the reaction temperatures applied for TMSN_3_, and there are also several examples for DMSO and H_2_O media. Tetrazole syntheses achieved with various benzonitriles also generally gave higher yields, but here the reliability of comparing the data is limited by the common tendency to publish only reactions with good yields. However, in most cases, catalysts had relatively complex preparation procedures (multistep immobilization) and expensive components (Pd, La, nanoparticles).

It is difficult to compare the energy and material costs of the syntheses, but on the basis solely of metal composition, the following can be stated unequivocally: palladium is extremely expensive due to its widespread use and the fact that it is one of the rarest elements in the Earth’s crust. The prices of other metals (Cr, Cu, Zn, and La) are several orders of magnitude lower, as these metals are relatively abundant in the Earth’s crust. However, zinc is the cheapest according to 2025 world market prices, costing nearly half as much as the other metals, which are roughly similar in price. Furthermore, during the operation, metal components of catalysts could easily form reactive salts by coordinating with azide anions, which hinder their reuse potential from both safety and feasibility perspectives. These facts highlight not only the safety of zinc-rich Zn_*x*_Cr-LDH/TMSN_3_ systems, but also the additional advantages of Zn­(II) and Cr­(III) ions, which are relatively biocompatible and inexpensive/readily available, and the robust crystal structure of LDHs and corresponding MMO derivatives. This confirms the idea that relatively simple LDH structures may be just as relevant in tetrazole synthesis as the currently popular MOF, silicate, and magnetic nanoparticle composite systems.^68,77,106–109^

## 4 Conclusions

A novel synthetic method has been developed for the 1*H*-tetrazole synthesis with Zn- and Cr-containing LDH and MMO catalysts under sustainable conditions. The applicability of the reactions was demonstrated with a wide variety of aromatic nitriles and different electron-withdrawing and electron-donating substituents, affording outstanding to moderate conversions. Reusability of Zn_3_Cr-LDH was also demonstrated, and the used catalyst was examined by XRD, IR and Raman spectroscopy, SEM-EDX, and TEM. Based on the results, excellent reusability of the catalyst could be attributed to the robustness of the catalytic surface.

Thermogravimetric and reaction extension studies (using various catalyst calcination pretreatments and benzonitrile reagents) revealed that in addition to Lewis acid Zn­(II)/Cr­(III) and basic O^2–^ centers, the Brönsted base OH^–^ units and the Lewis base interlamellar chloride anions could also have a significant effect on the observed activities of the LDH forms. Although KYNA-based tetrazoles could not be produced catalytically, four new KYNA-based nitriles were synthesized and fully characterized by NMR/MS techniques.

Zn_*x*_Cr-LDHs are primarily known for their utilization as photocatalysts, but the results presented herein have shown that they could also be applied as efficient heterogeneous thermocatalysts in other base-catalyzed and/or cycloaddition reactions. Compared to other methods and catalytic systems, these solids have the advantage of being easily accessible and safe to handle. They work under relatively mild conditions (24 h at 160 °C/48 h at 130 °C, only 2 equiv of TMSN_3_, and 10 mol % catalyst loading), and both LDH and MMO catalyst forms can be effectively used in the less environmentally harmful DMSO solvent.

## Supplementary Material



## Notes

Includes additional tables and figures, the analytical data of the reaction products (^1^H, ^13^C NMR), and the collection of NMR spectra from KYNA nitriles and tetrazoles (PDF)

## References

[1] Malik M. A., Wani M. Y., Al-Thabaiti S. A., Shiekh R. A. (2014). Tetrazoles as carboxylic
acid isosteres: chemistry and biology. J. Incl.
Phenom. Macrocycl. Chem..

[2] Ostrovskii V., Popova E. A., Trifonov R. (2017). Developments in Tetrazole
Chemistry
(2009–16). Adv. Heterocycl. Chem..

[3] Neochoritis C. G., Zhao T., Dömling A. (2019). Tetrazoles
via Multicomponent Reactions. Chem. Rev..

[4] Nasrollahzadeh M., Nezafat Z., Bidgoli N. S. S., Shafiei N. (2021). Use of tetrazoles in
catalysis and energetic applications: Recent development. Mol. Catal..

[5] Kabi, A. K. ; Sravani, S. ; Gujjarappa, R. ; Garg, A. ; Vodnala, N. ; Tyagi, U. ; Kaldhi, D. ; Velayutham, R. ; Singh, V. ; Gupta, S. , Nanostructured Biomaterials (Eds.: Swain, B. P. ; Springer: Singapore, 2022; pp. 307–349.

[6] Frija L. M., Ismael A., Cristiano M. L. S. (2010). Photochemical Transformations of
Tetrazole Derivatives: Applications in Organic Synthesis. Molecules.

[7] Bayannavar P. K., Kamble R. R., Joshi S. D., Nesaragi A. R., Shaikh S. K. J., Sudha B. S., Dodamani S. S., Hoolageri S. R. (2022). Design
and Synthesis of Angiotensin Converting Enzyme (ACE) Inhibitors: Analysis
of the Role of Tetrazole Ring Appended to Biphenyl Moiety. ChemistrySelect.

[8] Mikolaichuk O. V., Popova E. A., Protas A. V., Shemchuk O. S., Vasina L. V., Pavlyukova Y. N., Potanin A. A., Molchanov O. E., Maistrenko D. N., Semenov K. N., Sharoyko V. V. (2022). Study of biocompatibility,
cytotoxic activity in vitro of a tetrazole-containing derivative of
2-amino-4,6-di­(aziridin-1-yl)-1,3,5-triazine. Biochem. Bioph. Res. Commun..

[9] Ikeda T., Kakegawa H., Miyataka H., Matsumoto H., Satoh T. (1992). Anti-allergic and anti-inflammatory actions of 2′-(tetrazole-5-yl)-4-hydroxy-2-methyl-2H-1,2-benzothiazine-3-carboxanilide
1,1-dioxide. Bioorg. Med. Chem. Lett..

[10] Kushwaha P., Fatima S., Upadhyay A., Gupta S., Bhagwati S., Baghel T., Siddiqi M. I., Nazir A., Sashidhara K. V. (2019). Synthesis,
biological evaluation and molecular dynamic simulations of novel Benzofuran-tetrazole
derivatives as potential agents against Alzheimer’s disease. Bioorg. Med. Chem. Lett..

[11] Trifonov R. E., Ostrovskii V. A. (2023). Tetrazoles
and Related Heterocycles as Promising Synthetic
Antidiabetic Agents. Int. J. Mol. Sci..

[12] Li Y., Pasunooti K. K., Li R.-J., Liu W., Head S. A., Shi W. Q., Liu J. O. (2018). Novel Tetrazole-Containing Analogues
of Itraconazole as Potent Antiangiogenic Agents with Reduced Cytochrome
P450 3A4 Inhibition. J. Med. Chem..

[13] Gao F., Xiao J., Huang G. (2019). Current scenario of tetrazole hybrids
for antibacterial activity *Eur*. J. Med. Chem..

[14] Romagnoli R., Baraldi P. G., Salvador M. K., Preti D., Tabrizi M. A., Brancale A., Fu X.-H., Li J., Zhang S.-Z., Hamel E. (2012). Synthesis and Evaluation of 1,5-Disubstituted Tetrazoles
as Rigid Analogues of Combretastatin A-4 with Potent Antiproliferative
and Antitumor Activity. J. Med. Chem..

[15] Chi X., Zhang H., Wu H., Li X., Li L., Jiang Y., Ni T. (2023). Discovery of Novel
Tetrazoles Featuring
a Pyrazole Moiety as Potent and Highly Selective Antifungal Agents. ACS Omega.

[16] Biot C., Bauer H., Schirmer R. H., Davioud-Charvet E. (2004). 5-Substituted
Tetrazoles as Bioisosteres of Carboxylic Acids. Bioisosterism and
Mechanistic Studies on Glutathione Reductase Inhibitors as Antimalarials. J. Med. Chem..

[17] Nĕmeček J., Sychra P., Macháček M., Benková M., Karabanovich G., Konečná K., Kavková V., Stolaříková J., Hrabálek A., Vávrová K., Soukup O., Roh J., Klimešová V. (2017). Structure-activity relationship studies
on 3,5-dinitrophenyl tetrazoles as antitubercular agents. Eur. J. Med. Chem..

[18] Zarubaev V. V., Golod E. L., Anfimov P. M., Shtro A. A., Saraev V. V., Gavrilov A. S., Logvinov A. V., Kiselev O. I. (2010). Synthesis
and anti-viral
activity of azolo-adamantanes against influenza A virus. Bioorgan. Med. Chem..

[19] Myznikov L. V., Vorona S. V., Zevatskii Y. E. (2021). Biologically
active compounds and
drugs in the tetrazole series. Chem. Heterocycl.
Compd..

[20] Rózsa É., Robotka H., Vécsei L., Toldi J. (2008). The Janus-Face Kynurenic
Acid. J. Neural. Transm..

[21] Stone T. W. (2001). Kynurenic
Acid Antagonists and Kynurenine Pathway Inhibitors. Expert Opin. Invest. Drugs.

[22] Wang J., Simonavicius N., Wu X., Swaminath G., Reagan J., Tian H., Ling L. (2006). Kynurenic
Acid as a
Ligand for Orphan G Protein-Coupled Receptor GPR35. J. Biol. Chem..

[23] Sas K., Robotka H., Toldi J., Vécsei L. (2007). Mitochondria
Metabolic Disturbances, Oxidative Stress and the Kynurenine System,
with Focus on Neurodegenerative Disorders. J.
Neurol. Sci..

[24] Beal M. F., Matson W. R. K. J., Swartz K. J., Gamache P. H. E. D., Bird E. D. (1990). Kynurenine Pathway Measurements in Huntington’s
Disease Striatum: Evidence for Reduced Formation of Kynurenic Acid. J. Neurochem..

[25] Schwarcz R., Rassoulpour A., Wu H.-Q., Medoff D. C. A., Tamminga C. A., Roberts R. C. (2001). Increased
Cortical Kynurenate Content in Schizophrenia. Biol. Psychiatry.

[26] Roh J., Vávrová K., Hrabálek A. (2012). Synthesis
and Functionalization of 5-Substituted Tetrazoles. Eur. J. Org. Chem..

[27] Maleki A., Sarvary A. (2015). Synthesis of tetrazoles via isocyanide-based reactions. RSC Adv..

[28] Stepanov A. I., Sannikov V. S., Dashko D. V., Roslyakov A. G., Astrat’ev A. A., Stepanova E. V. (2015). A new preparative
method and some
chemical properties of 4-R-furazan-3-carboxylic acid amidrazones. Chem. Heterocycl. Compd..

[29] Treitler D. S., Leung S., Lindrud M. (2017). Development
and Demonstration of
a Safer Protocol for the Synthesis of 5-Aryltetrazoles from Aryl Nitriles. Org. Process Res. Dev..

[30] Mittal R., Awasthi S. K. (2019). Recent Advances
in the Synthesis of 5-Substituted 1H-Tetrazoles:
A Complete Survey (2013–2018). Synthesis.

[31] Du H.-C., Matzuk M. M., Chen Y.-C. (2020). Synthesis of 5-substituted tetrazoles
via DNA-conjugated nitrile. Org. Biomol. Chem..

[32] Swami S., Sahu S. N., Shrivastava R. (2021). Nanomaterial
catalyzed green synthesis
of tetrazoles and its derivatives: a review on recent advancements. RSC Adv..

[33] Leyva-Ramos S., Cardoso-Ortiz J. (2021). Recent Developments
in the Synthesis of Tetrazoles
and their Pharmacological Relevance. Curr. Org.
Chem..

[34] Ghfar A. A., Albadran F. H. A., Liu X. (2024). Construction
and Characterization
of Magnetic Fe_3_O_4_ Nanoparticles Supported Palladium
Complex: Research on Synthesis of Aryl Nitriles and Tetrazoles. Catal. Lett..

[35] Jaiswal S., Dwivedi J., Kishore D., Sharma S. (2024). Green Methodologies
for Tetrazole Synthesis from Different Starting Materials: A Recent
Update. Curr. Org. Chem..

[36] Xu Z. P., Zhang J., Adebajo M. O., Zhang H., Zhou C. (2011). Catalytic
applications of layered double hydroxides and derivatives. Appl. Clay Sci..

[37] Xu M., We M. (2018). Layered Double Hydroxide-Based
Catalysts: Recent Advances in Preparation,
Structure, and Applications. Adv. Funct. Mater..

[38] Evans, D. G. ; Slade, R. C. T. in Layered Double Hydroxides, Vol. 119 Eds.: Duan, X. ; Evans, D. G. ; Springer-Verlag: Berlin, 2006; Vol. 119, pp. 1–87.

[39] Wang Q., O’Hare D. (2012). Recent Advances in the Synthesis
and Application of
Layered Double Hydroxide (LDH) Nanosheets. Chem.
Rev..

[40] Li, F. ; Duan, X. Layered Double Hydroxides, Eds.: Duan, X. ; Evans, D. G. ;Springer-Verlag: Berlin, 2006; Vol. 119, pp. 193–224.

[41] Yang Z., Wei J., Zeng G., Zhang H., Tan X., Ma C., Li X., Li Z., Zhang C. (2019). A review on strategies to LDH-based
materials to improve adsorption capacity and photoreduction efficiency
for CO_2_. Coordin Chem. Rev..

[42] Boumeriame H., Da Silva E. S., Cherevan A. S., Chafik T., Faria J. L., Eder D. (2022). Layered double hydroxide
(LDH)-based materials: A mini-review on
strategies to improve the performance for photocatalytic water splitting. J. Energy Chem..

[43] Bi X., Zhang H., Dou L. (2014). Layered Double Hydroxide-Based Nanocarriers
for Drug Delivery. Pharmaceutics.

[44] Karádi K., Nguyen T.-T., Ádám A.
A., Baán K., Sápi A., Kukovecz Á., Kónya Z., Sipos P., Pálinkó I., Varga G. (2023). Structure–activity
relationships of LDH catalysts for the glucose-to-fructose isomerisation
in ethanol. Green Chem..

[45] Bing W., Zheng L., He S., Rao D., Xu M., Zheng L., Wang B., Wang Y., Wei M. (2018). Insights on
Active Sites of CaAl-Hydrotalcite as a High-Performance Solid Base
Catalyst toward Aldol Condensation. ACS Catal..

[46] Jia H., Zhao Y., Niu P., Lu N., Fan B., Li R. (2018). Amine-functionalized
MgAl LDH nanosheets as efficient solid base
catalysts for Knoevenagel condensation. Mol.
Catal..

[47] Izquierdo-Aranda L., Adam R., Cabrero-Antonino J. R. (2023). Silver Supported Nanoparticles on
[Mg4Al-LDH] as an Efficient Catalyst for the α-Alkylation of
Nitriles, Oxindoles and Other Carboxylic Acid Derivatives with Alcohols. ChemSuschem.

[48] Fan G., Li F., Evans D. G., Duan X. (2014). Catalytic applications of layered
double hydroxides: recent advances and perspectives. Chem. Soc. Rev..

[49] Lee S.-B., Ko E.-H., Park J. Y., Oh J.-M. (2021). Mixed Metal Oxide
by Calcination of Layered Double Hydroxide: Parameters Affecting Specific
Surface Area. Nanomaterials.

[50] Bencherif S. D., Gallardo J. J., Carrillo-Berdugo I., Bahmani A., Navas J. (2021). Synthesis
Characterization and Photocatalytic Performance of Calcined ZnCr-Layered
Double Hydroxides. Nanomaterials.

[51] Wu L., Liu D., Chen F., Zhou H., Shi R., Liu Y., Zhang J., Zhu Y., Wang J. (2023). Efficient methane oxidation
to oxygenates over etched ZnCr layered double hydroxide nanosheets. J. Mater. Chem. A.

[52] Ma X., Xu Y., Tan L., Zhao Y., Song Y.-F. (2020). Visible-Light-Induced
Hydrogenation of C=C Bonds by Hydrazine over Ultrathin Layered
Double Hydroxide Nanosheets. Ind. Eng. Chem.
Res..

[53] Gil-Gavilán D. G., Cosano D., Amaro-Gahete J., Castillo-Rodríguez M., Esquivel D., Ruiz J. R., Romero-Salguero F. J. (2023). Zn-Cr Layered
Double Hydroxides for Photocatalytic Transformation of CO2 under Visible
Light Irradiation: The Effect of the Metal Ratio and Interlayer Anion. Catalysts.

[54] Rana S., Kumar A., Lai C. W., Sharma G., Dhiman P. (2024). Recent progress
in ZnCr and NiCr layered double hydroxides and based photocatalysts
for water treatment and clean energy production. Chemosphere.

[55] Parida K., Mohapatra L. (2012). Recent progress
in the development of carbonate-intercalated
Zn/Cr LDH as a novel photocatalyst for hydrogen evolution aimed at
the utilization of solar light. Dalton Trans..

[56] Yao L., Wei D., Yan D., Hu C. (2015). ZnCr Layered Double Hydroxide (LDH)
Nanosheets Assisted Formation of Hierarchical Flower-Like CdZnS@LDH
Microstructures with Improved Visible-Light-Driven H2 Production. Chem Asian J..

[57] Béres A., Pálinkó I., Kiricsi I., Mizukami F. (2001). Characterization
and catalytic activity of Ni–Al and Zn–Cr mixed oxides
obtained from layered double hydroxides. Solid
State Ion..

[58] Sahoo M., Parida K. (2021). Noble metal loaded
ZnCr-LDH based hybrid material for
Suzuki coupling reactions: A comparison study on heterogeneous catalysis
with photo catalysis. Mater. Today: Proc..

[59] Saikia P., Saikia J., Sarmah S., Allou N. G. B., Goswamee R. L. (2017). Mesoporous
oxidic holey nanosheets from Zn-Cr LDH synthesized by soft chemical
etching of Cr^3+^ and its application as CO_2_ hydrogenation
catalyst. J. CO2 Util..

[60] Bahranowski K., Bielańska E., Janik R., Machej T., Serwicka E. M. (1999). LDH-derived
catalysts for complete oxidation of volatile organic compounds. Clay Miner.

[61] Mitani Y., Takei T., Yanagida S., Kumada N. (2017). Hybridization of Ni–Cr,
Cu–Cr, and Zn–Cr layered double hydroxides with polyoxometalates
and their catalytic behavior. J. Ceram. Soc.
Jpn..

[62] Thao N. T., Nga H. T. P., Vo N. Q., Nguyen H. D. K. (2017). Advanced oxidation
of rhodamine B with hydrogen peroxide over Zn-Cr layered double hydroxide
catalysts. J. Sci.: adv. Mater. Devices.

[63] Zhao Y., Xie R., Lin Y., Fan G., Li F. (2018). Highly efficient solvent-free
aerobic oxidation of ethylbenzene over hybrid Zn–Cr layered
double hydroxide/carbon nanotubes nanocomposite. Catal. Commun..

[64] Meng Y., Chen Y., Zhou X., Pan G., Xia S. (2020). Experimental
and theoretical investigations into the activity and mechanism of
the water-gas shift reaction catalyzed by Au nanoparticles supported
on Zn-Al/Cr/Fe layered double hydroxides. Int.
J. Hydrogen Energy.

[65] Vishwakarma R., Gadipelly C., Mannepalli L. K. (2022). Advances in Tetrazole Synthesis –
An Overview. ChemistrySelect.

[66] Palde P. B., Jamison T. F. (2011). Safe and Efficient Tetrazole Synthesis
in a Continuous-Flow
Microreactor. Angew. Chem., Int. Ed..

[67] Naeimi H., Kiani F. (2015). Ultrasound-promoted one-pot three component synthesis of tetrazoles
catalyzed by zinc sulfide nanoparticles as a recyclable heterogeneous
catalyst. Ultrason. Sonochem..

[68] Koolivand M., Nikoorazm M., Ghorbani-Choghamarani A., Mohammadi M. (2022). A novel cubic
Zn-citric acid-based MOF as a highly efficient and reusable catalyst
for the synthesis of pyranopyrazoles and 5-substituted 1*H*-tetrazoles. Appl. Organomet. Chem..

[69] Joshi S. M., Mane R. B., Pulagam K. R., Gomez-Vallejo V., Llop J., Rode C. (2017). The microwave-assisted
synthesis
of 5-substituted 1H-tetrazoles via [3 + 2] cycloaddition over a heterogeneous
Cu-based catalyst: application to the preparation of 13N-labelled
tetrazoles. New J. Chem..

[70] Bugris V., Ádok-Sipiczki M., Anitics T., Kuzmann E., Homonnay Z., Kukovecz Á., Kónya Z., Sipos P., Pálinkó I. (2015). Thermal decomposition
and reconstruction of CaFe-layered double hydroxide studied by X-ray
diffractometry and 57Fe Mössbauer spectroscopy. J. Mol. Struct..

[71] Szabados M., Ádám A. A., Traj P., Muráth S., Baán K., Bélteky P., Kónya Z., Kukovecz Á., Sipos P., Pálinkó I. (2020). Mechanochemical
and wet chemical syntheses of CaIn-layered double hydroxide and its
performance in a transesterification reaction compared to those of
other Ca2M­(III) hydrocalumites (M: Al, Sc, V, Cr, Fe, Ga) and Mg­(II)-,
Ni­(II)-, Co­(II)- or Zn­(II)-based hydrotalcites. J. Catal..

[72] Xiong Q., Dong S., Chen Y., Liu X., Feng X. (2019). Asymmetric
synthesis of tetrazole and dihydroisoquinoline derivatives by isocyanide-based
multicomponent reactions. Nat. Commun..

[73] Dhiman N., Kaur K., Jaitak V. (2020). Tetrazoles as anticancer
agents:
A review on synthetic strategies, mechanism of action and SAR studies. Bioorgan. Med. Chem..

[74] Hibino T. (2004). Delamination
of Layered Double Hydroxides Containing Amino Acids. Chem. Mater..

[75] Zhao Y., Yang W., Xue Y., Wang X., Lin T. (2011). Partial exfoliation
of layered double hydroxides in DMSO: a route to transparent polymer
nanocomposites. J. Mater. Chem..

[76] Alfonsi K., Colberg J., Dunn P. J., Fevig T., Jennings S., Johnson T. A., Kleine H. P., Knight C., Nagy M. A., Perry D. A., Stefaniak M. (2008). Green chemistry
tools to influence
a medicinal chemistry and research chemistry based organization. Green Chem..

[77] Ashrafa M. A., Liu Z., Yang Y., Lia C., Zhang D. (2020). Magnetic nanomaterials
catalyzed synthesis of tetrazoles. Synth. Commun..

[78] Cao Z., Zhang L., Liang K., Cheong S., Boyer C., Gooding J. J., Chen Y., Gu Z. (2018). Biodegradable 2D Fe–Al
Hydroxide for Nanocatalytic Tumor-Dynamic Therapy with Tumor Specificity. Adv. Sci..

[79] Constantino V. R. L., Figueiredo M. P., Magri V. R., Eulálio D., Cunha V. R. R., Alcântara A. C.
S., Perotti G. F. (2023). Biomaterials
Based on Organic Polymers and Layered Double Hydroxides Nanocomposites:
Drug Delivery and Tissue Engineering. Pharmaceutics.

[80] Paušová S., Krýsa J., Jirkovskýd J., Forano C., Mailhot G., Prevot V. (2015). Insight into
the photocatalytic activity of ZnCr–CO_3_ LDH and
derivedmixed oxides. Appl.
Catal., B.

[81] Wittenberger S. J., Donner B. G. (1993). Dialkyltin oxide
mediated addition of trimethylsilyl
azide to nitriles. A novel preparation of 5-substituted tetrazoles. J. Org. Chem..

[82] Chretien J.-M., Kerric G., Zammattio F., Galland N., Paris M., Quintard J.-P., Le Grognec E. (2019). Tin-catalyzed
synthesis of 5-substituted
1H-tetrazoles from nitriles: Homogeneous and heterogeneous procedures. Adv. Synth. Catal..

[83] Himo F., Demko Z. P., Noodleman L., Sharpless K. B. (2003). Why is
tetrazole formation by addition of azide to organic nitriles catalyzed
by zinc­(II) salts?. J. Am. Chem. Soc..

[84] Mani P., Singh A. K., Awasthi S. K. (2014). AgNO_3_ catalyzed synthesis
of 5-substituted-1H-tetrazole via [3 + 2] cycloaddition of nitriles
and sodium azide. Tetrahedron Lett..

[85] Simon P., Lőrinczi B., Hetényi A., Szatmári I. (2023). Novel Eco-friendly
One-Pot Method for the Synthesis of Kynurenic Acid Ethyl Esters. ACS Omega.

[86] Binev Y. I., Petrova R. R., Tsenov J. A., Binev I. G. (2000). IR spectra
and structure
of (4-nitrophenyl)­acetonitrile and of its carbanion: experimental
and ab initio studies. J. Mol. Struct..

[87] Wallace V. M., Dhumal N. R., Zehentbauer F. M., Kim H. J., Kiefer J. (2015). Revisiting
the Aqueous Solutions of Dimethyl Sulfoxide by Spectroscopy in the
Mid- and Near-Infrared: Experiments and Car–Parrinello Simulations. J. Phys. Chem. B.

[88] Jemini J., Singh S., Pal B. (2022). Efficient
ZnCr LDH/monoclinic-WO_3_ composites for Degradation of Tetracycline
under Visible
Light. ChemistrySelect.

[89] Kang J., Levitskaia T. G., Park S., Kim J., Varga T., Um W. (2020). Nanostructured MgFe and CoCr layered
double hydroxides for removal
and sequestration of iodine anions. Chem. Eng.
J..

[90] Tao X., Yang C., Huang L., Shang S. (2020). Novel plasma assisted
preparation of ZnCuFeCr layered double hydroxides with improved photocatalytic
performance of methyl orange degradation. Appl.
Surf. Sci..

[91] Kim J., Kang J., Um W. (2022). Simultaneous
removal of cesium and
iodate using prussian blue functionalized CoCr layered double hydroxide
(PB-LDH*)*. J. Environ. Chem.
Eng..

[92] Szabó V., Mészáros R., Kutus B., Samu G. F., Kónya Z., Kukovecz Á., Sipos P., Szabados M. (2025). Sonochemically
improved regeneration of mechanically amorphized Ca_2_Cr-layered
double hydroxides – Synthesis, characterization and photocatalytic
lidocaine decomposition. Appl. Clay Sci..

[93] Treitler D. S., Leung S. (2022). How dangerous is too dangerous? A
perspective on azide chemistry. J. Org. Chem..

[94] Ötvös S. B., Mészáros R., Varga G., Kocsis M., Kónya Z., Kukovecz Á., Pusztai P., Sipos P., Pálinkó I., Fülöp F. (2018). A mineralogically-inspired
silver–bismuth hybrid material: an efficient heterogeneous
catalyst for the direct synthesis of nitriles from terminal alkynes. Green Chem..

[95] Guberman M., Pieber B., Seeberger P. H. (2019). Safe and
scalable continuous flow
azidophenylselenylation of galactal to prepare galactosamine building
blocks. Org. Process Rev. Dev..

[96] Huff B. E., Staszak M. A. (1993). A new method for the preparation of tetrazoles from
nitriles using trimethylsilylazide/trimethylaluminum. Tetrahedron Lett..

[97] Amantini D., Beleggia R., Fringuelli F., Pizzo F., Vaccaro L. (2004). TBAF-catalyzed
synthesis of 5-substituted 1H-tetrazoles under solventless conditions. J. Org. Chem..

[98] Jin T., Kitahara F., Kamijo S., Yamamoto Y. (2008). Synthesis of 5-substituted
1H-tetrazoles by the copper-catalyzed [3 + 2] cycloaddition of nitriles
and trimethylsilyl azide. Chem. —Asian
J..

[99] Meshram G. A., Deshpande S. S., Wagh P. A., Vala V. A. (2014). Silica supported
lanthanum triflate mediated synthesis of 5-substituted-1H tetrazoles. Tetrahedron Lett..

[100] Wang Z., Liu Z., Cheon S. H. (2015). Facile
synthesis
of 5-substituted 1H-tetrazoles catalyzed by tetrabutylammonium hydrogen
sulfate in water. Bull. Korean Chem. Soc..

[101] Razavi N., Akhlaghinia B. (2015). Cu­(II) immobilized on aminated epichlorohydrin
activated silica (CAES): as a new, green and efficient nanocatalyst
for preparation of 5-substituted-1H-tetrazoles. RSC Adv..

[102] Nikoorazm M., Ghorbani-Choghamarani A., Khanmoradi M. (2016). Application
of Pd-2A3HP-MCM-41 to the Suzuki, Heck and Stille coupling reactions
and synthesis of 5-substituted 1H-tetrazoles. Appl. Organometal. Chem..

[103] Ashraf M. A., Liu Z., Li C., Zhang D. (2021). Fe 3 O 4 @L-lysine-Pd(0)
organic–inorganic hybrid: As a novel heterogeneous magnetic
nanocatalyst for chemo and homoselective [2 + 3] cycloaddition synthesis
of 5-substituted 1H-tetrazoles. Appl. Organomet.
Chem..

[104] Karimian A., Emarloo N., Salari S. (2021). The mineral
alum: an
effective and low-cost heterogeneous catalyst for the successful synthesis
of 5-substituted-1H-tetrazoles. Inorg. Nano-Met.
Chem..

[105] Nozaria A., Hassania H., Karimian A. (2023). ZnFe_2_O_4_@SiO_2_–SO_3_H magnetic nanoparticles:
A new, efficient, and recyclable heterogeneous nanocatalyst for successful
synthesis of 5-substituted-1H-tetrazoles. Russ.
J. Org. Chem..

[106] Ghorbani-Choghamarani A., Shiri L., Azadi G. (2016). The first
report on the eco-friendly synthesis of 5-substituted 1H-tetrazoles
in PEG catalyzed by Cu­(II)­immobilized on Fe_3_O_4_@SiO_2_@L-arginine as a novel, recyclable and non-corrosive
catalyst. RSC Adv..

[107] Tamoradi T., Veisi H., Karmakar B., Gholami J. (2020). A competent
green methodology for the synthesis of aryl thioethers and 1H-tetrazole
over magnetically retrievable novel CoFe_2_O_4_@L-asparagine
anchored Cu, Ni nanocatalyst. Mater. Sci. Eng.,
C.

[108] Swami, S. ; Sharma, N. ; Shrivastava, R. Silica-Supported Heterogenous Catalysts: Application in the Synthesis of Tetrazoles Solid Base Catalysts: synthesis, Characterization, And Applications Wiley 2024 233–258 10.1002/9783527846719.ch8

[109] Priyanka, Yadav S., Rana P., Bandichhor R., Srivastava A., Sharma R. K. (2024). Unexplored catalytic potency of a magnetic CoFe_2_O_4_/Ni-BDC MOF composite for the one-pot sustainable
synthesis of 5-substituted 1-H tetrazoles. Chem.
Eng. J..

