# Supplementary material for: Thermocatalytic Transformation of Nitriles Utilizing Pristine and Calcined ZnCr Layered Double Hydroxides for the Synthesis of Various Tetrazole- and Kynurenic Acid-Based Drug Candidates
Source: ACS Omega. 2026 Mar 4;11(10):16392–405. doi: 10.1021/acsomega.5c12277 (PMC13000588; doi:10.1021/acsomega.5c12277)
Supplement: Supplementary file 1 [file ao5c12277_si_001.pdf]

Electronic Supporting Information

Thermocatalytic transformation of nitriles utilizing pristine and calcined ZnCr layered double hydroxides for the synthesis of various tetrazole- and kynurenic acid-based drug candidates

Hiba Alsoliman[a], Márton Szabados[b,c], Péter Bélteky[d], Zoltán Kónya[d,e], István Szatmári[a,f], Rebeka Mészáros[a*]

[a]*Institue of Pharmaceutical Chemistry, University of Szeged, Eötvös utca 6, Szeged, H-6720 Hungary*

[b]*Department of Molecular and Analytical Chemistry, University of Szeged, Dóm tér 8, Szeged, H-6720 Hungary*

[c]*Material and Solution Structure Research Group, Institute of Chemistry, University of Szeged, Aradi vértanúk tere 1, Szeged, H-6720 Hungary*

[d]*Department of Applied and Environmental Chemistry, University of Szeged, Rerrich B. tér 1, Szeged, H-6720 Hungary*

[e]*HUN-REN-SZTE Reaction Kinetics and Surface Chemistry Research Group, Rerrich B. tér 1, Szeged, H-6720 Hungary*

[f]*HUN-REN-SZTE Stereochemistry Research Group, Eötvös utca 6, Szeged, H-6720 Hungary*

Table of Contents

1. Additional Figures and Tables....................S2
2. Analytical Data of the Reaction Products S11
3. Collection of NMR Spectra S14
4. References S48

Correponding author: Rebeka Mészáros
E-mail address: meszaros.rebeka.ildiko@szte.hu (R. Mészáros)

1. Additional Figures and Tables

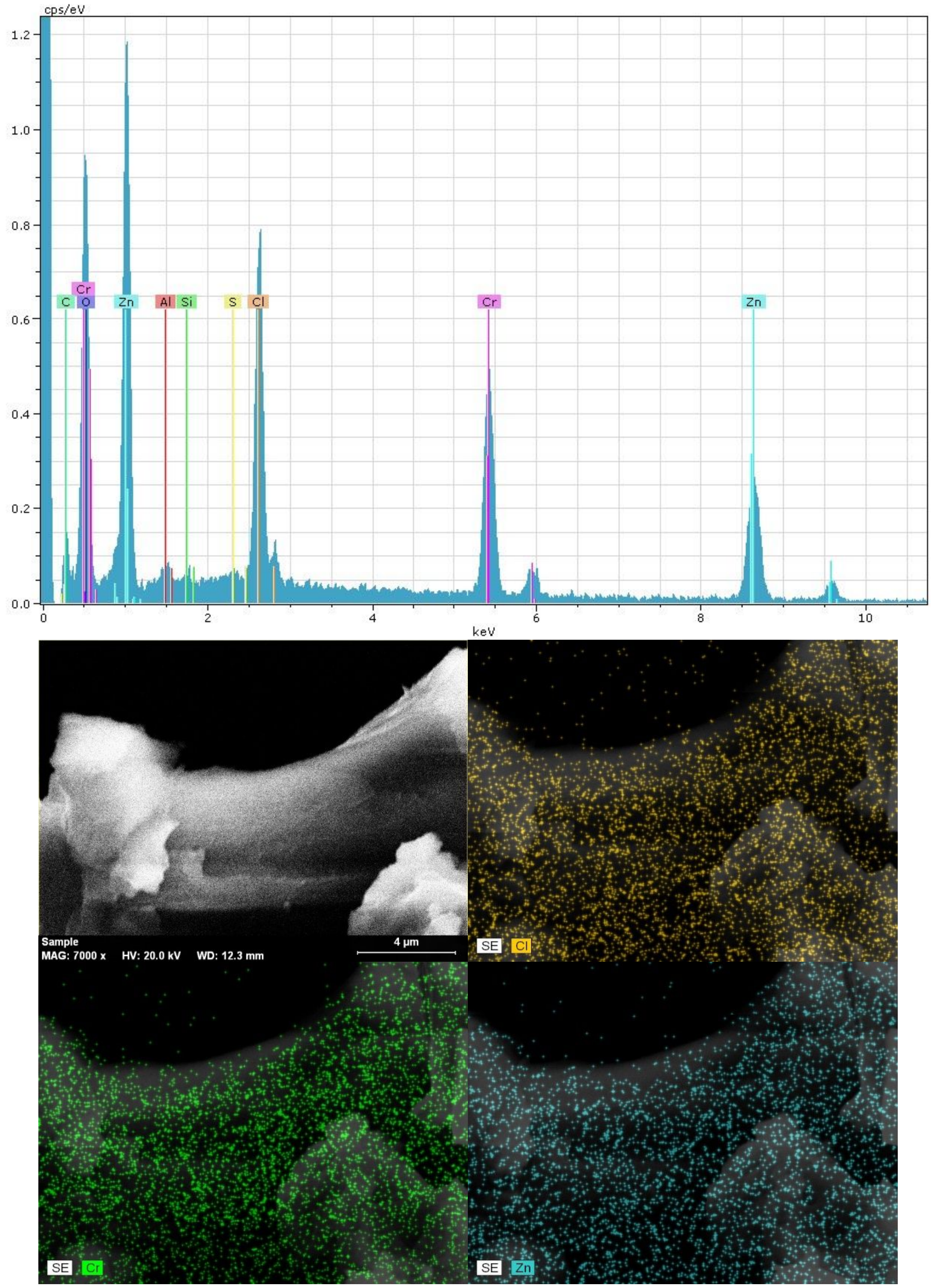

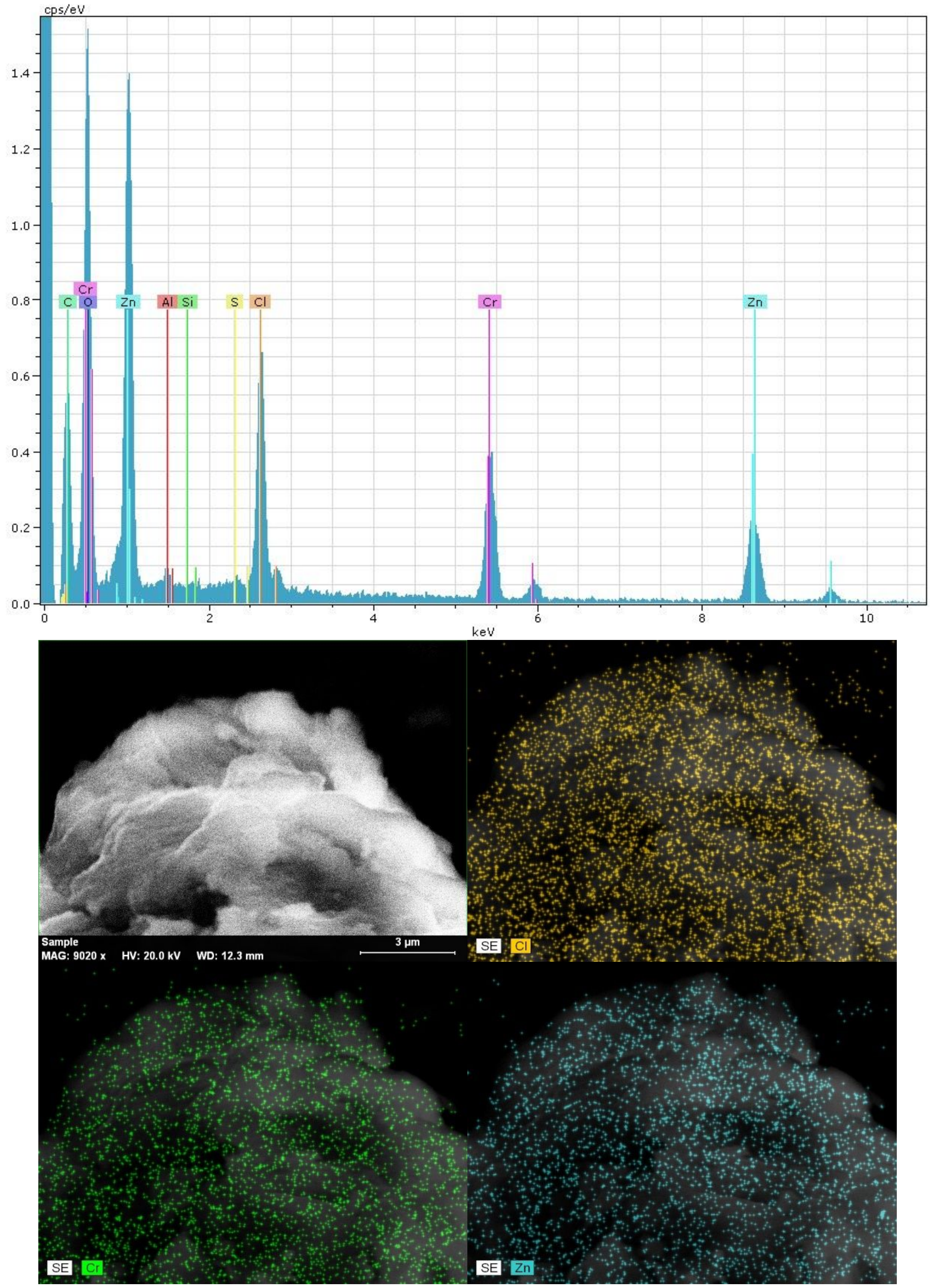

Figure S1 EDX spectra and the corresponding SEM photos and elemental maps of the pristine Zn_3Cr-LDH catalyst (the sign of the C, Al and Si atoms comes from the sample holder).

Table S1. Investigation of concentration in the synthesis of 5-(4-nitrophenyl)-1*H*-tetrazole from 4-nitrobenzonitrile and $TMSN_3$ as starting materials.

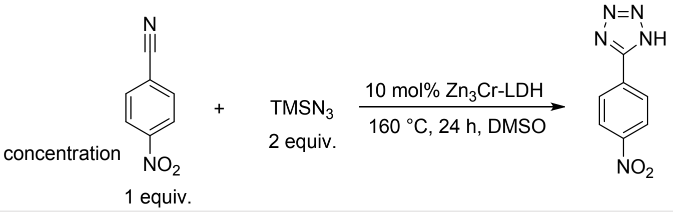

#	Conc. (M)	Conv. (%)[a]
1	0.1	90 ± 1.63
2	0.15	78 ± 5.79
3	0.2	72 ± 5.73
4	0.25	68 ± 4

Reaction conditions: 1 equiv. nitrile, 2 equiv. $TMSN_3$, 10 mol% catalyst, solvent: DMSO, 160 °C, 24 h reaction time. [a]Determined by ^{1}H NMR analysis of the crude products.

Table S2. Investigation of catalyst loading and the amount of the nitrogen source in the synthesis of 5-(4-nitrophenyl)-1*H*-tetrazole from 4-nitrobenzonitrile and $TMSN_3$ as starting materials.

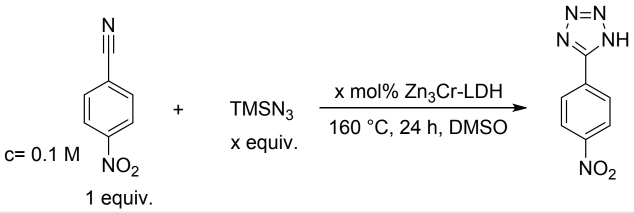

#	Nitrogen source amount (equiv.)	Catalyst loading (mol%)	Conv. (%)[a]
1	1	10	78 ± 2.49
2	2	10	90 ± 1.63
3	3	10	89 ± 0.47
4	2	1	22 ± 4.49
5	2	3	50 ± 4
6	2	5	70 ± 2
7	2	10	90 ± 1.63
8	2	15	91 ± 0.94

Reaction conditions: 1 equiv. nitrile (c= 0.1 M), solvent: DMSO, 160 °C, 24 h reaction time. [a]Determined by ^{1}H NMR analysis of the crude products.

Table S3. Investigation of Zn:Cr ratio of the catalyst and the calcination of the catalyst in the synthesis of 5-(4-nitrophenyl)-1*H*-tetrazole from 4-nitrobenzonitrile and $TMSN_3$ as starting materials.

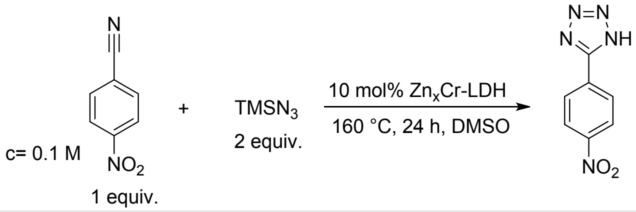

#	Zn : Cr ratio	Calcination temperature (°C)	Conv. (%)[a]
1	2:1	-	81 ± 4.08
2	3:1	-	90 ± 1.63
3	4:1	-	92 ± 2.16
4	5:1	-	93 ± 1.88
5	6:1	-	95 ± 1.24
6	3:1	300	66 ± 2.49
7	3:1	400	65 ± 1.41
8	3:1	500	68 ± 1.63
9	3:1	600	76 ± 1.24
10	3:1	700	62 ± 3.29
11	3:1	900	60 ± 4.78

Reaction conditions: 1 equiv. nitrile (c= 0.1 M), 2 equiv. $TMSN_3$, solvent: DMSO, 160 °C, 10 mol% catalyst, 24 h reaction time, 160 °C. [a]Determined by ^1H NMR analysis of the crude products.

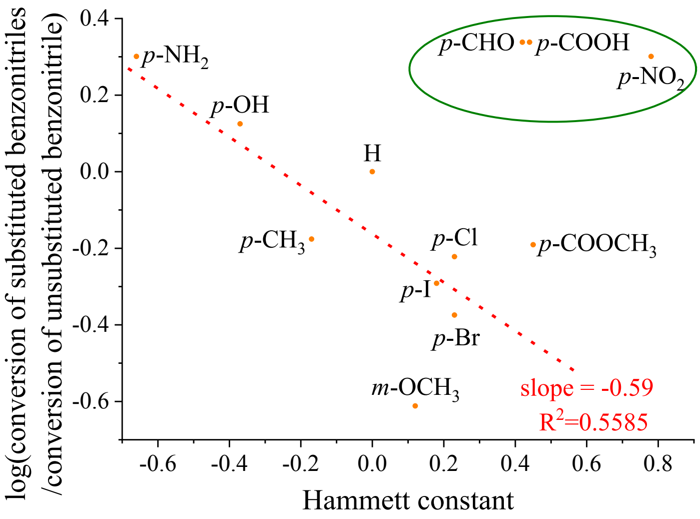

Figure S2 Hammett plot for the conversions of reactions in the study of tetrazole synthesis extension.

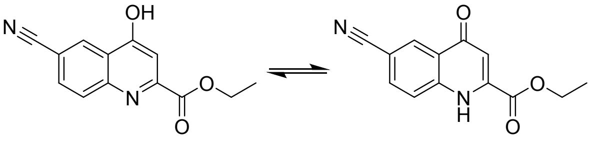

2a

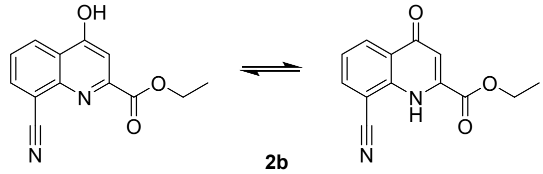

2b

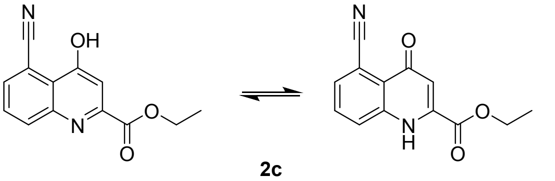

2c

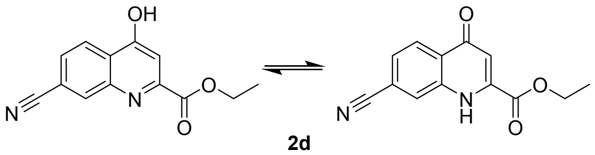

2d

Figure S3 Enolic- and oxo-form of KYNA nitriles 2a-2d.

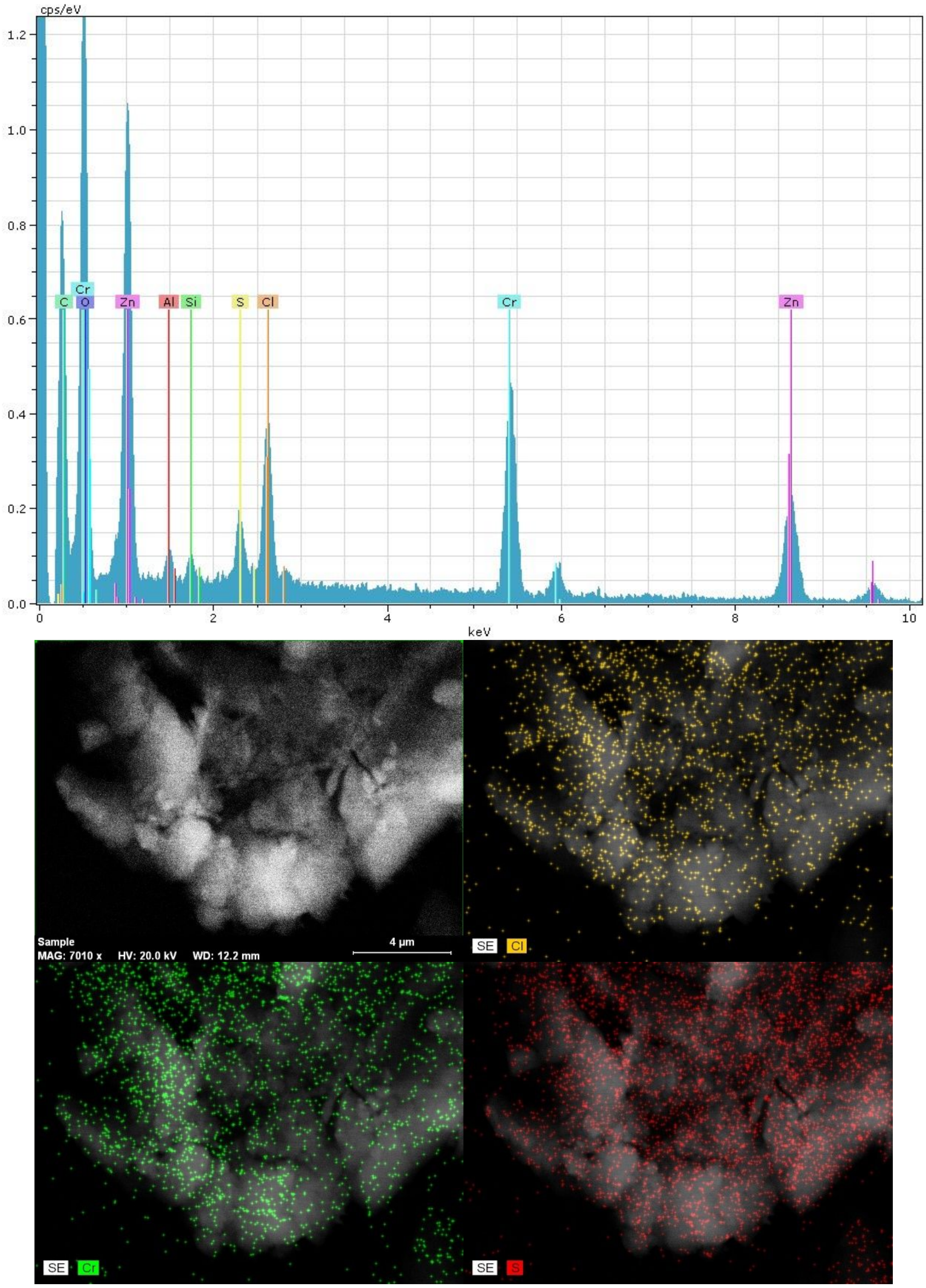

cps/eV
1.2
1.0
0.8
0.6
0.4
0.2
0.0
0
2
4
6
8
10
keV
C
Cr
O
Zn
Al
Si
S
Cl
Cr
Zn
Sample
MAG: 7010 x HV: 20.0 kV WD: 12.2 mm
4 µm
SE
Cl
SE
Cr
SE
S

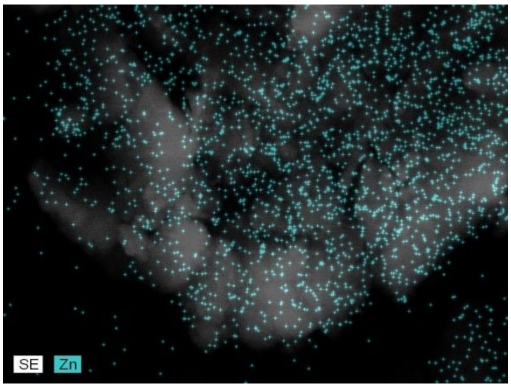
SE
Zn

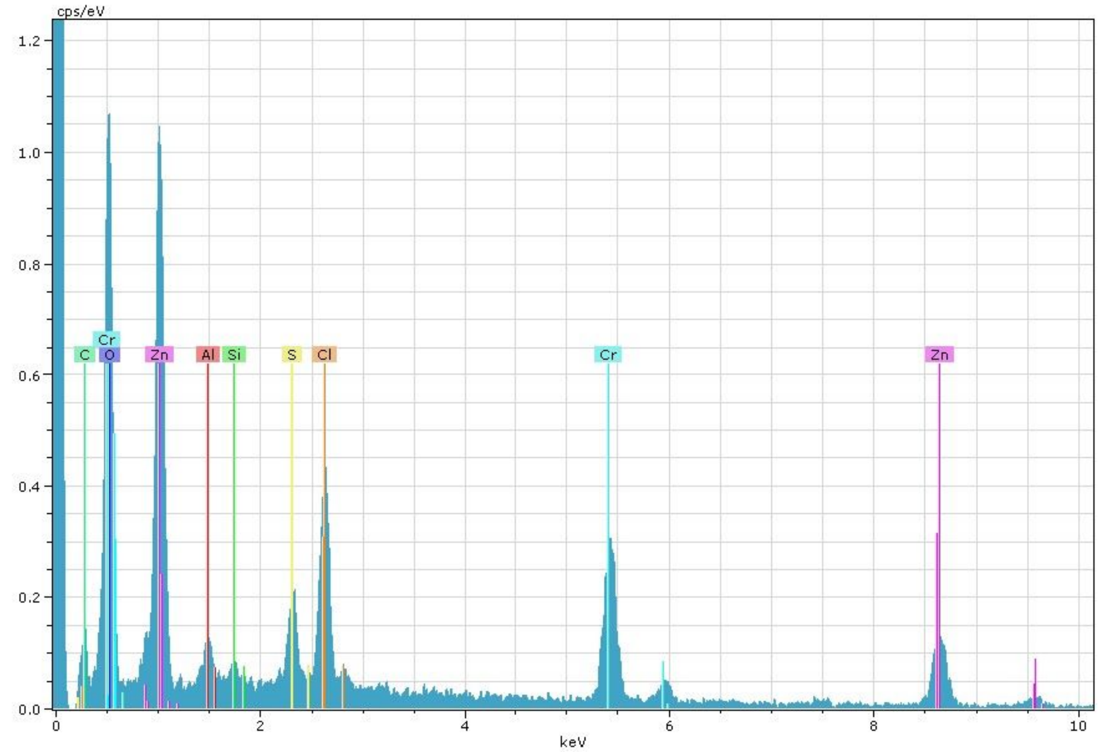
cps/eV
1.2
1.0
0.8
0.6
0.4
0.2
0.0
0
2
4
6
8
10
keV
C
Cr
O
Zn
Al
Si
S
Cl
Cr
Zn

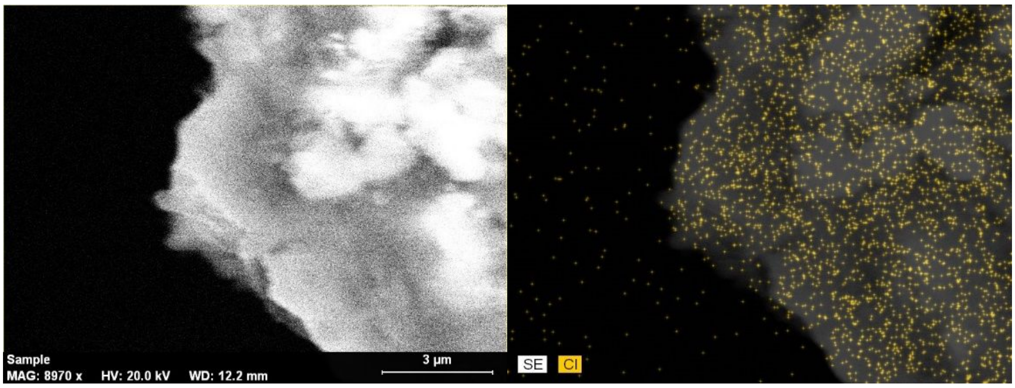
Sample
MAG: 8970 x HV: 20.0 kV WD: 12.2 mm
3 µm
SE
Cl

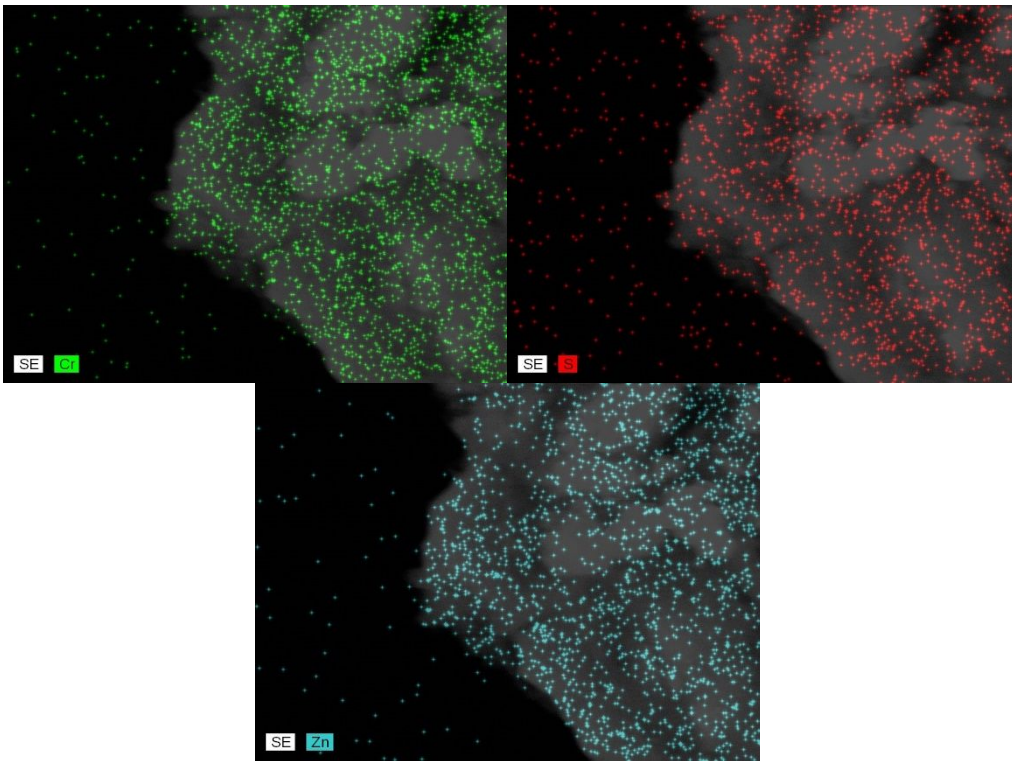

Figure S4 EDX spectra and the corresponding SEM photos and elemental maps of the spent Zn_3Cr-LDH catalyst (the sign of the C, Al and Si atoms comes from the sample holder).

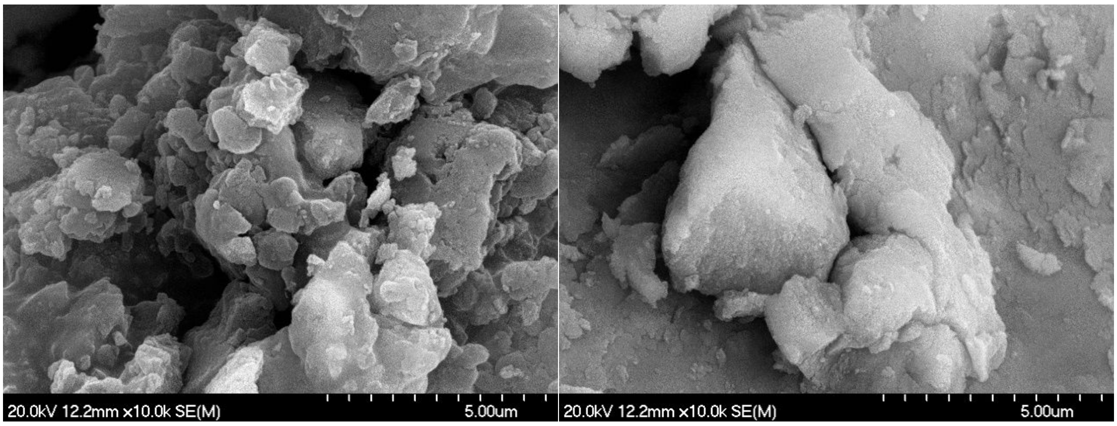

Figure S5 SEM photos of the pristine (left) and spent (right) Zn_3Cr-LDH catalysts.

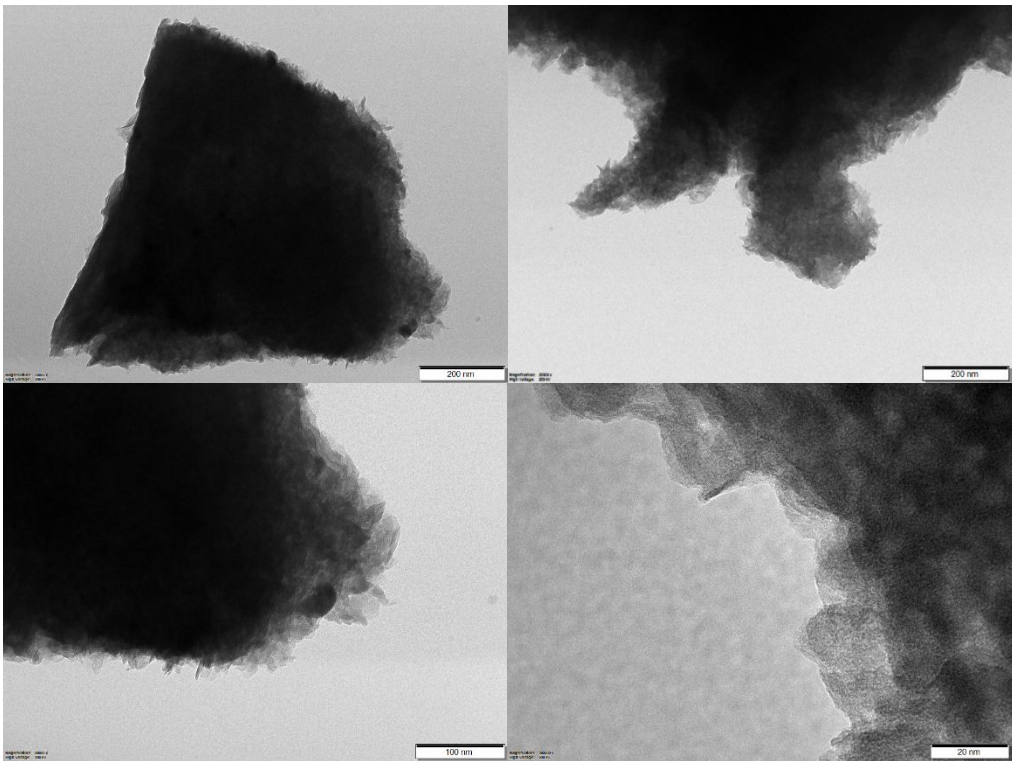

Figure S6 TEM photos of the pristine Zn_3Cr-LDH catalysts.

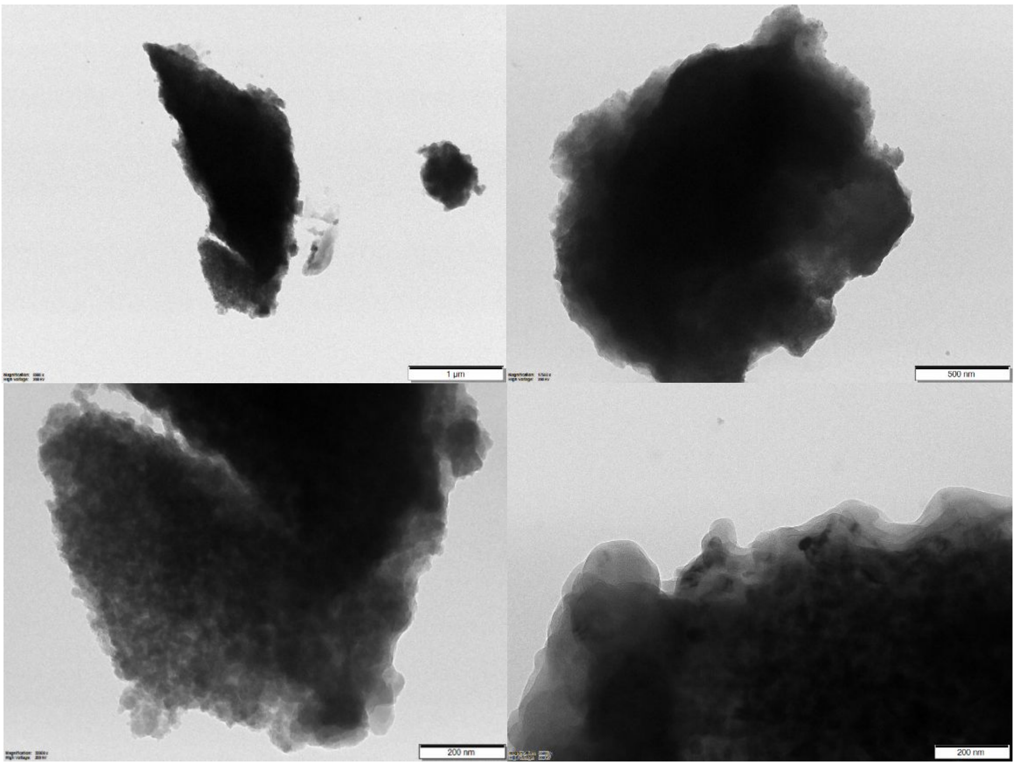

Figure S7 TEM photos of the spent Zn_3Cr-LDH catalysts.

2. Analytical Data of the Reaction Products

5-(4-Nitrophenyl)-1*H*-tetrazole

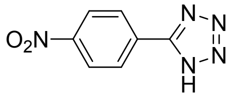

^{1}H NMR (500 MHz, DMSO-d6): δ=8.44-8.42 (d, J= 8.88 Hz, 2H), 8.36-8.34 (d, J= 8.88 Hz, 2H). ^{13}C NMR (125 MHz, DMSO-d6): 163.3, 149.1, 133.0, 128.2, 125.1. NMR data is in agreement with the published data.[1]

4-(1*H*-tetrazol-5-yl)benzaldehyde

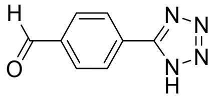

^{1}H NMR (500 MHz, DMSO-d6): δ= 10.10 (s, 1H) 8.31-8.30 (d, J= 7.91 Hz, 2H), 8.12-8.10 (d, J= 8.10 Hz, 2H). ^{13}C NMR (125 MHz, DMSO-d6): 193.2, 164.1, 137.9, 132.2, 130.9, 127.6. NMR data is in agreement with the published data.[2]

4-(1*H*-tetrazol-5-yl)-benzoic acid

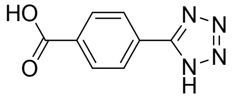

^{1}H NMR (500 MHz, DMSO-d6): δ= 13.27 (s, 1H) 8.20-8.19 (d, J= 8.17 Hz, 2H), 8.13-8.11 (d, J= 8.17 Hz, 2H). ^{13}C NMR (125 MHz, DMSO-d6): 167.2, 164.2, 133.2, 130.4, 128.7, 127.1. NMR data is in agreement with the published data.[3]

4-(1*H*-tetrazol-5-yl)aniline

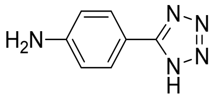

^{1}H NMR (500 MHz, DMSO-d6): δ= 7.50-7.49 (d, J= 8.70 Hz, 2H), 6.78-6.76 (d, J= 8.70 Hz, 2H). ^{13}C NMR (125 MHz, DMSO-d6): 163.1, 151.0, 133.6, 120.9, 113.9. NMR data is in agreement with the published data.[4]

4-(1*H*-tetrazol-5-yl)phenol

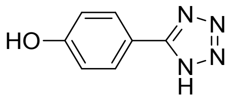

^{1}H NMR (500 MHz, DMSO-d6): δ= 7.79-7.77 (d, J= 8.78 Hz, 2H), 7.18-7.17 (d, J= 8.78 Hz, 2H). ^{13}C NMR (125 MHz, DMSO-d6): 160.8, 154.6, 134.5, 117.3, 114.8. NMR data is in agreement with the published data.[1]

5-Phenyl-1*H*-tetrazole

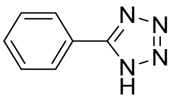

^{1}H NMR (500 MHz, DMSO-d6): δ= 8.09-8.07 (m, 2H), 7.58-7.57 (m, 3H). ^{13}C NMR (125 MHz, DMSO-d6): 164.9, 131.2, 129.8, 127.2, 126.9. NMR data is in agreement with the published data.[1]

5-(4-Methylphenyl)-1*H*-tetrazole

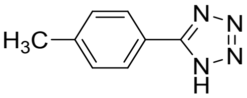

^{1}H NMR (500 MHz, DMSO-d6): δ= 7.77-7.76 (d, J= 8.17 Hz, 2H), 7.29-7.27 (d, J= 8.17 Hz, 2H), 2.36 (s, 3H). ^{13}C NMR (125 MHz, DMSO-d6): 166.5, 141.8, 129.3, 127.7, 21.4. NMR data is in agreement with the published data.[1]

Methyl 4-(1*H*-tetrazol-5-yl)benzoate

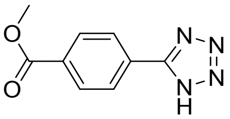

^{1}H NMR (500 MHz, DMSO-d6): δ= 8.22-8.20 (d, J= 8.43 Hz, 2H), 8.15-8.13 (d, J= 8.43 Hz, 2H), 3.90 (s, 3H). ^{13}C NMR (125 MHz, DMSO-d6): 166.2, 161.1, 147.7, 131.6, 130.6, 127.1, 52.9. NMR data is in agreement with the published data.[5]

5-(4-Chlorophenyl)-1*H*-tetrazole

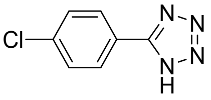

^{1}H NMR (500 MHz, DMSO-d6): δ= 8.09-8.08 (d, J= 8.76 Hz, 2H), 7.66-7.64 (d, J= 8.27 Hz, 2H). ^{13}C NMR (125 MHz, DMSO-d6): 164.1, 135.9, 130.0, 128.7, 126.0. NMR data is in agreement with the published data.[6]

5-(4-Iodophenyl)-1*H*-tetrazole

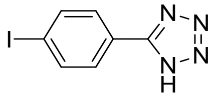

^{1}H NMR (500 MHz, DMSO-d6): δ= 7.97-7.96 (d, J= 8.20 Hz, 2H), 7.87-7.85 (d, J= 8.20 Hz, 2H). ^{13}C NMR (125 MHz, DMSO-d6): 157.0, 138.7, 128.7, 126.6, 98.5. NMR data is in agreement with the published data.[7]

5-(4-Bromophenyl)-1*H*-tetrazole

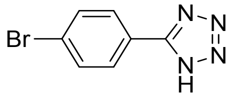

^{1}H NMR (500 MHz, DMSO-d6): δ= 8.03-8.01 (d, J= 8.43 Hz, 2H), 7.80-7.78 (d, J= 8.43 Hz, 2H). ^{13}C NMR (125 MHz, DMSO-d6): 164.1, 132.9, 128.9, 126.4, 124.6. NMR data is in agreement with the published data.[6]

5-(3-Methoxyphenyl)-1*H*-tetrazole

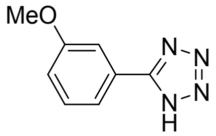

^{1}H NMR (500 MHz, DMSO-d6): δ= 7.67-7.65 (d, J= 7.72 Hz, 1H), 7.57 (m, 1H), 7.51-7.48 (t, J= 8.15 Hz, 1H), 7.14-7.12 (dd, J= 2.34 and 8.43 Hz, 1H), 3.85 (s, 3H). ^{13}C NMR (125 MHz, DMSO-d6): 160.3, 157.3, 131.1, 128.5, 119.2, 117.1, 111.8, 55.7. NMR data is in agreement with the published data.[8]

5-(naphthalen-2-yl)-1*H*-tetrazole

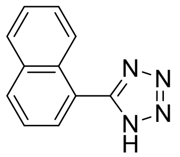

^{1}H NMR (500 MHz, DMSO-d6): δ= 8.49 (m, 1H), 8.18-8.16 (d, J= 8.17 Hz, 1H), 8.12-8.11 (d, J= 8.17 Hz, 1H), 8.07-8.06 (d, J= 8.17 Hz, 1H), 7.94-7.92 (d, J= 8.17 Hz, 1H), 7.71-7.65 (m, 2H). ^{13}C NMR (125 MHz, DMSO-d6): 154.2, 134.2, 132.8, 129.8, 129.5, 129.3, 128.7, 128.3, 127.7, 125.6, 121.4. NMR data is in agreement with the published data.[1]

2. Collection of NMR Spectra

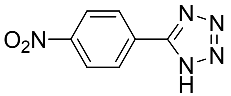

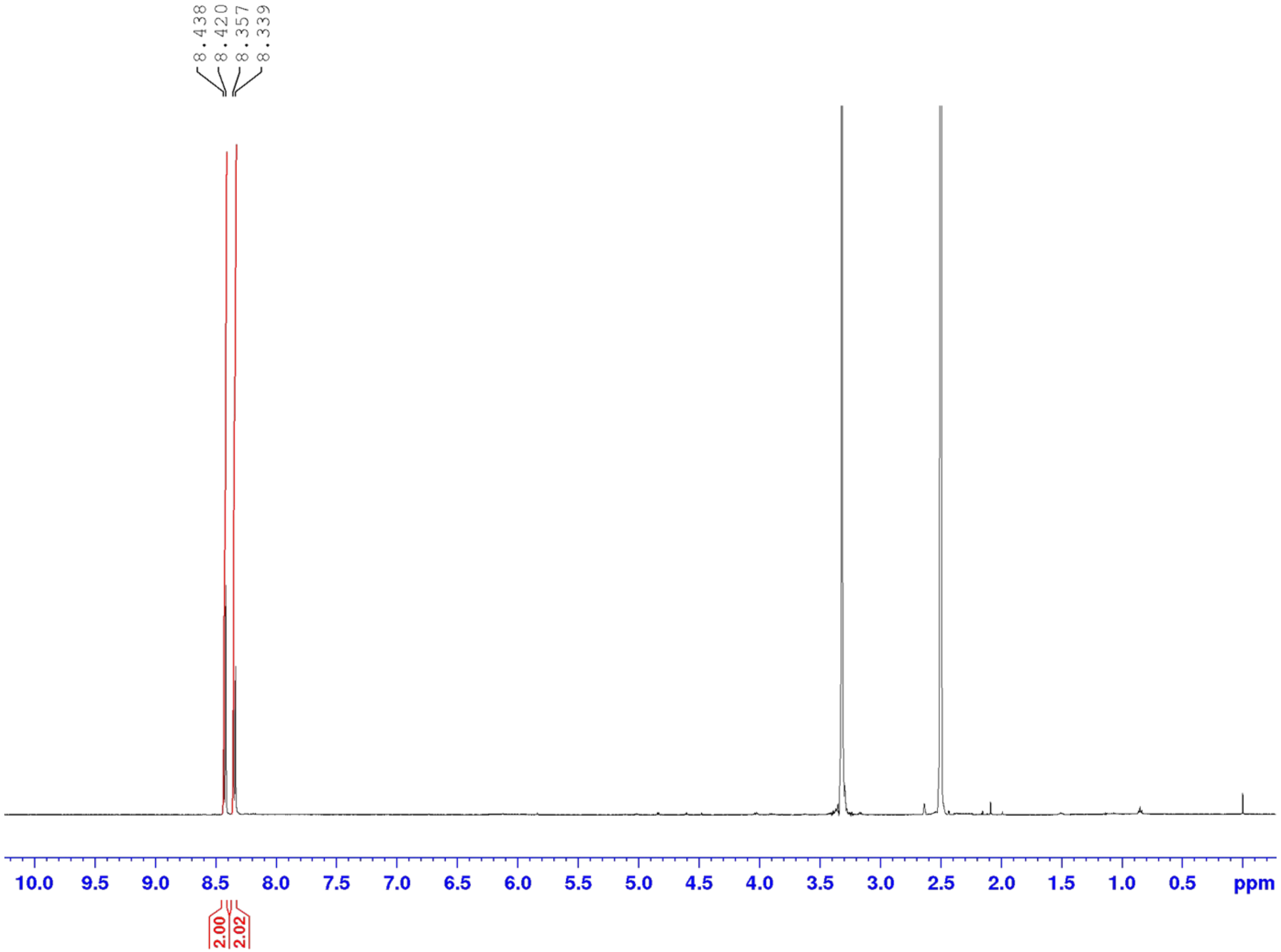

Graph S1 ^{1}H NMR spectrum of 5-(4-nitrophenyl)-1*H*-tetrazole.

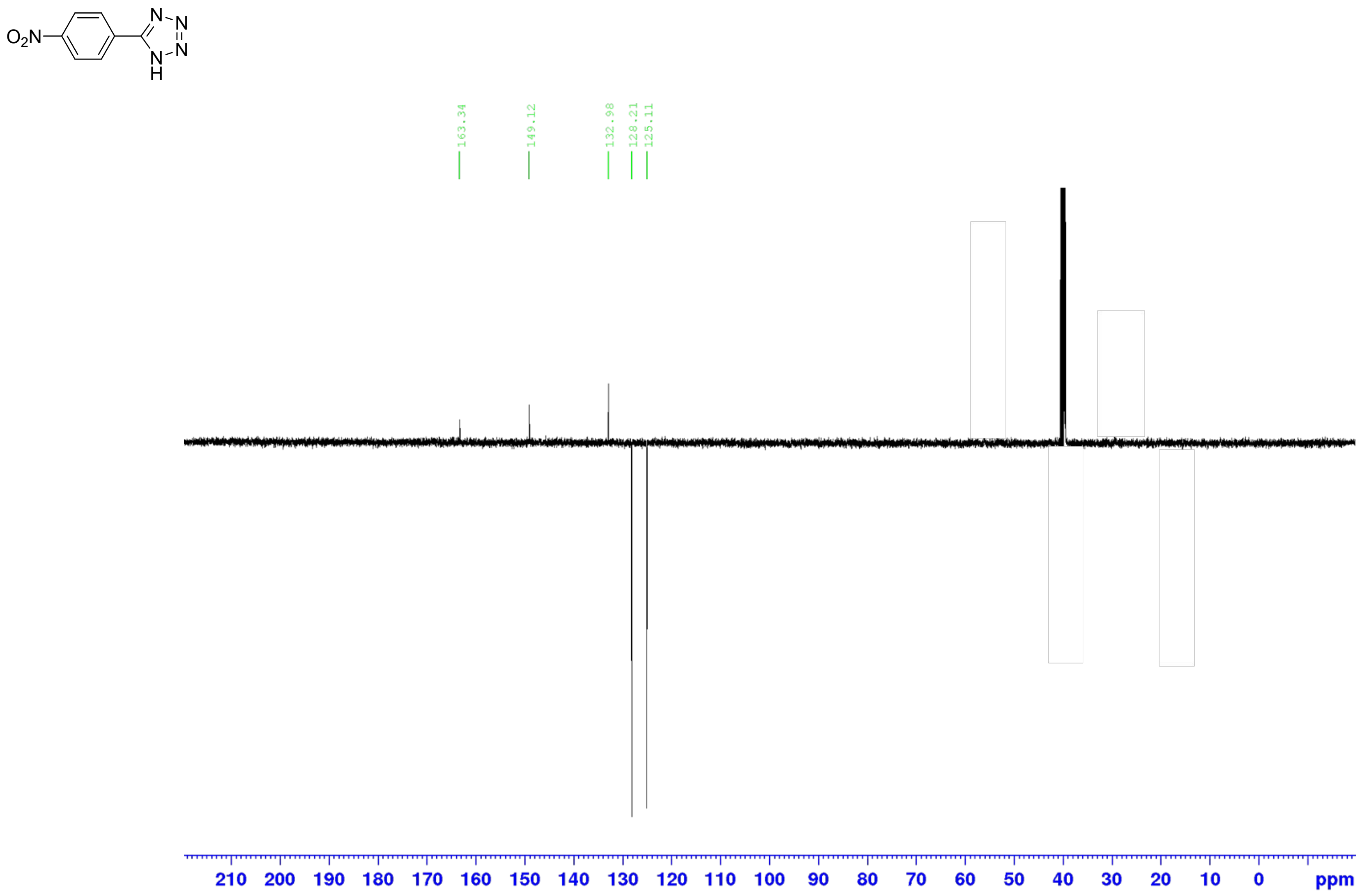

Graph S2 ^{13}C NMR spectrum of 5-(4-nitrophenyl)-1*H*-tetrazole.

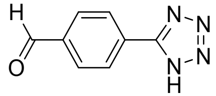

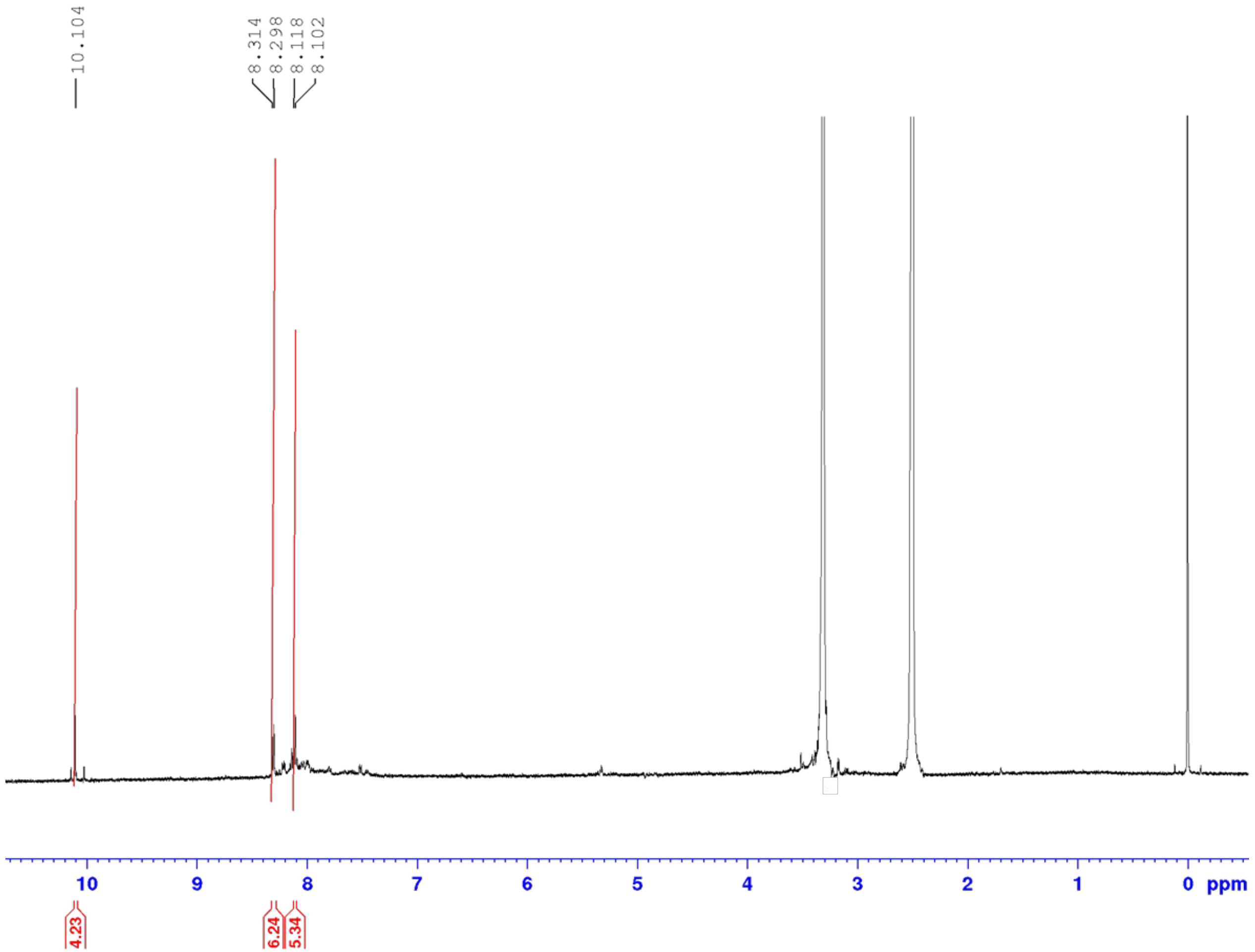

Graph S3 ^{1}H NMR spectrum of 4-(1*H*-tetrazol-5-yl)benzaldehyde.

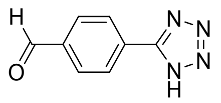

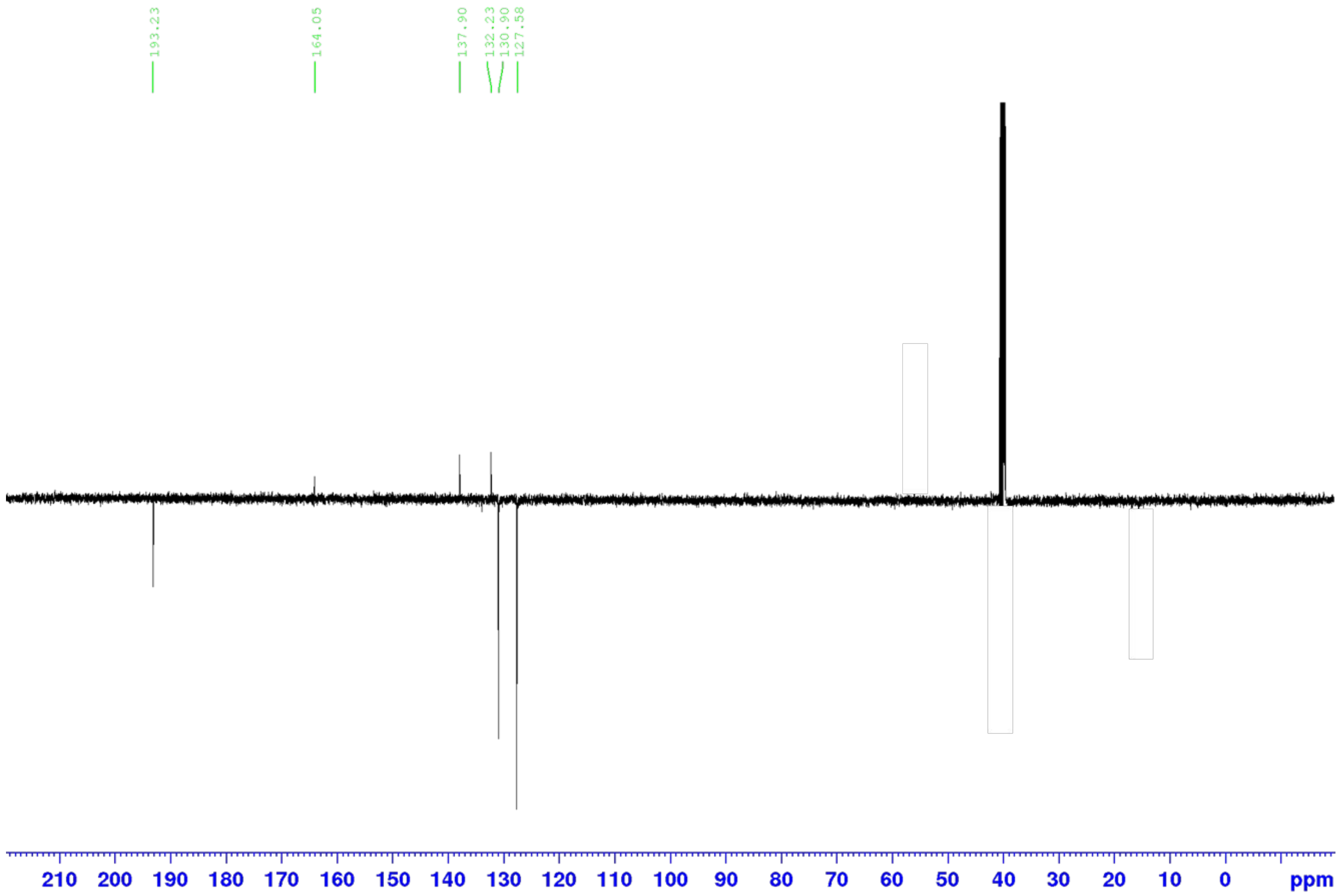

Graph S4 ^{13}C NMR spectrum of 4-(1*H*-tetrazol-5-yl)benzaldehyde.

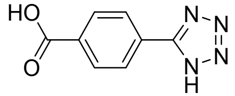

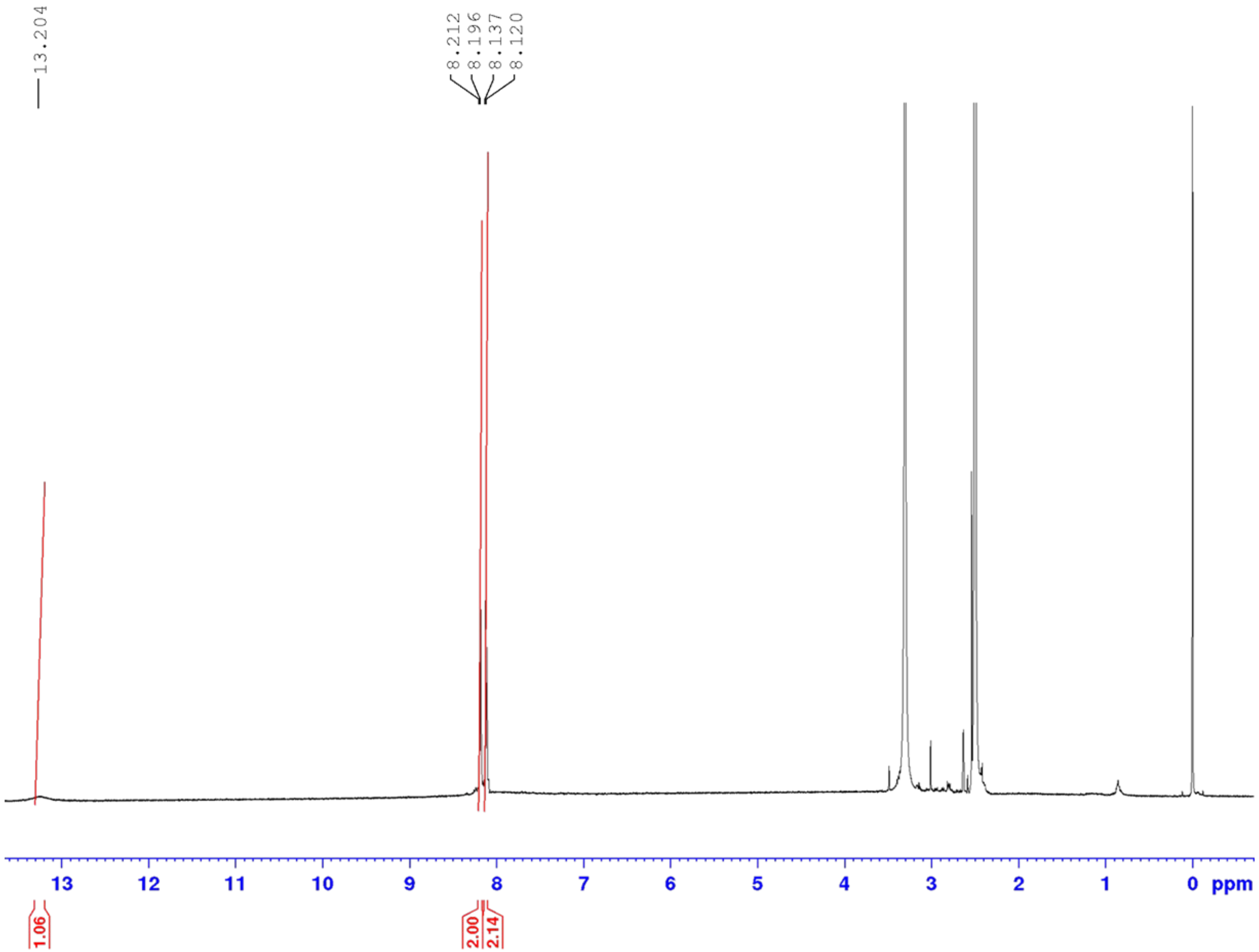

Graph S5 ^{1}H NMR spectrum of 4-(1*H*-tetrazol-5-yl)-benzoic acid.

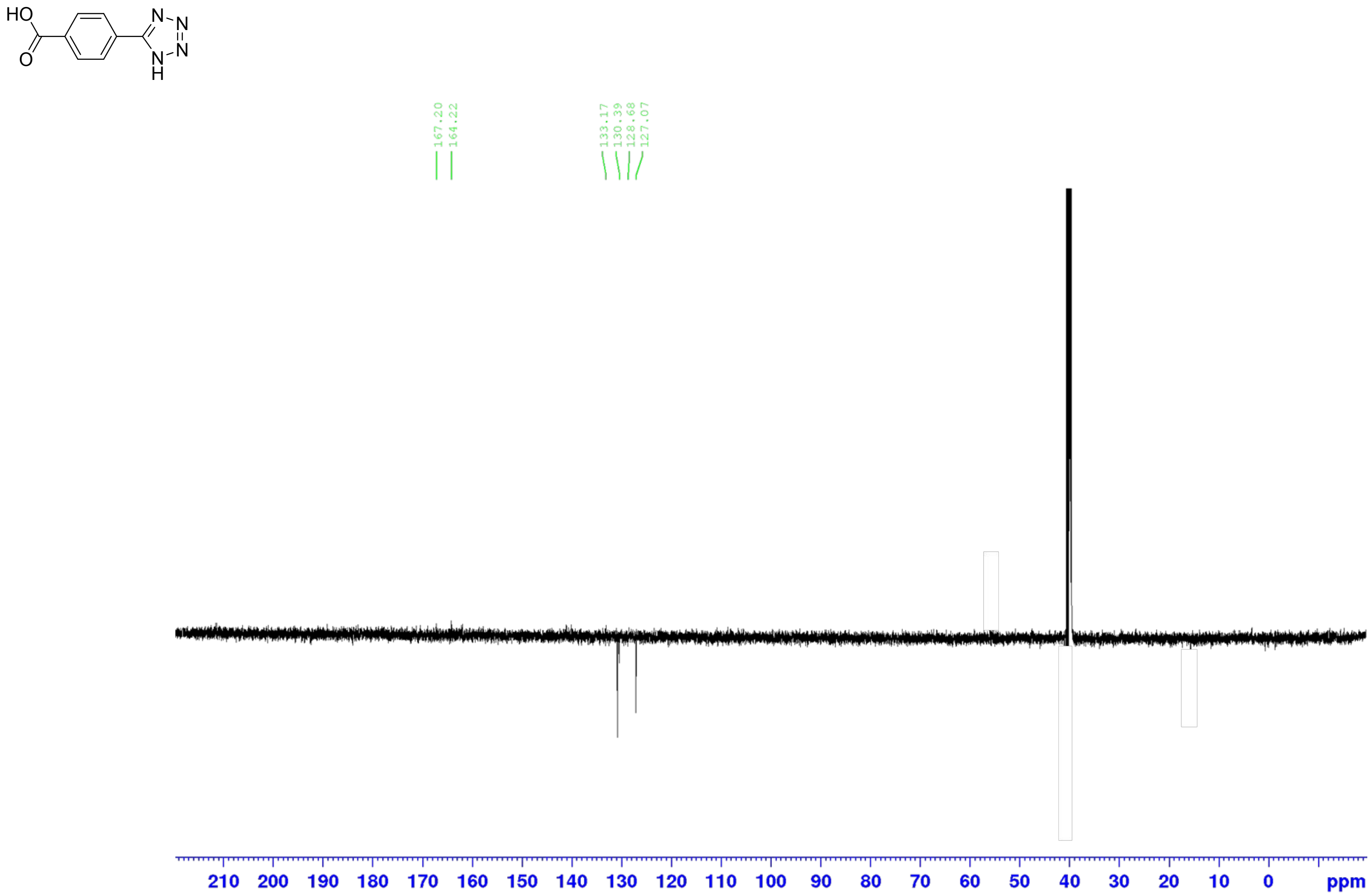

Graph S6 ^{13}C NMR spectrum of 4-(1*H*-tetrazol-5-yl)-benzoic acid.

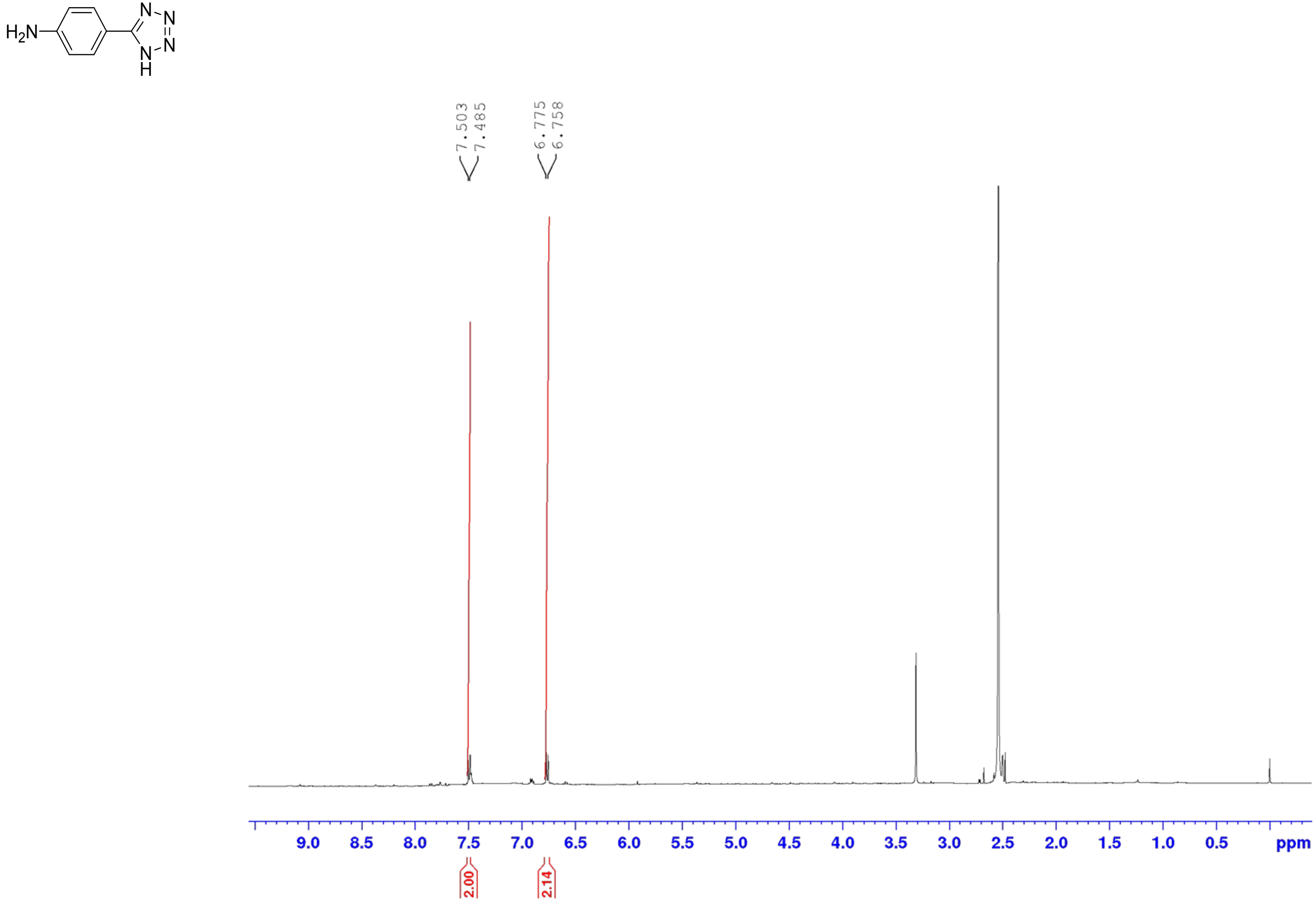

Graph S7 ^{1}H NMR spectrum of 4-(1*H*-tetrazol-5-yl)aniline.

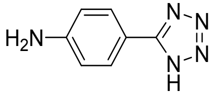

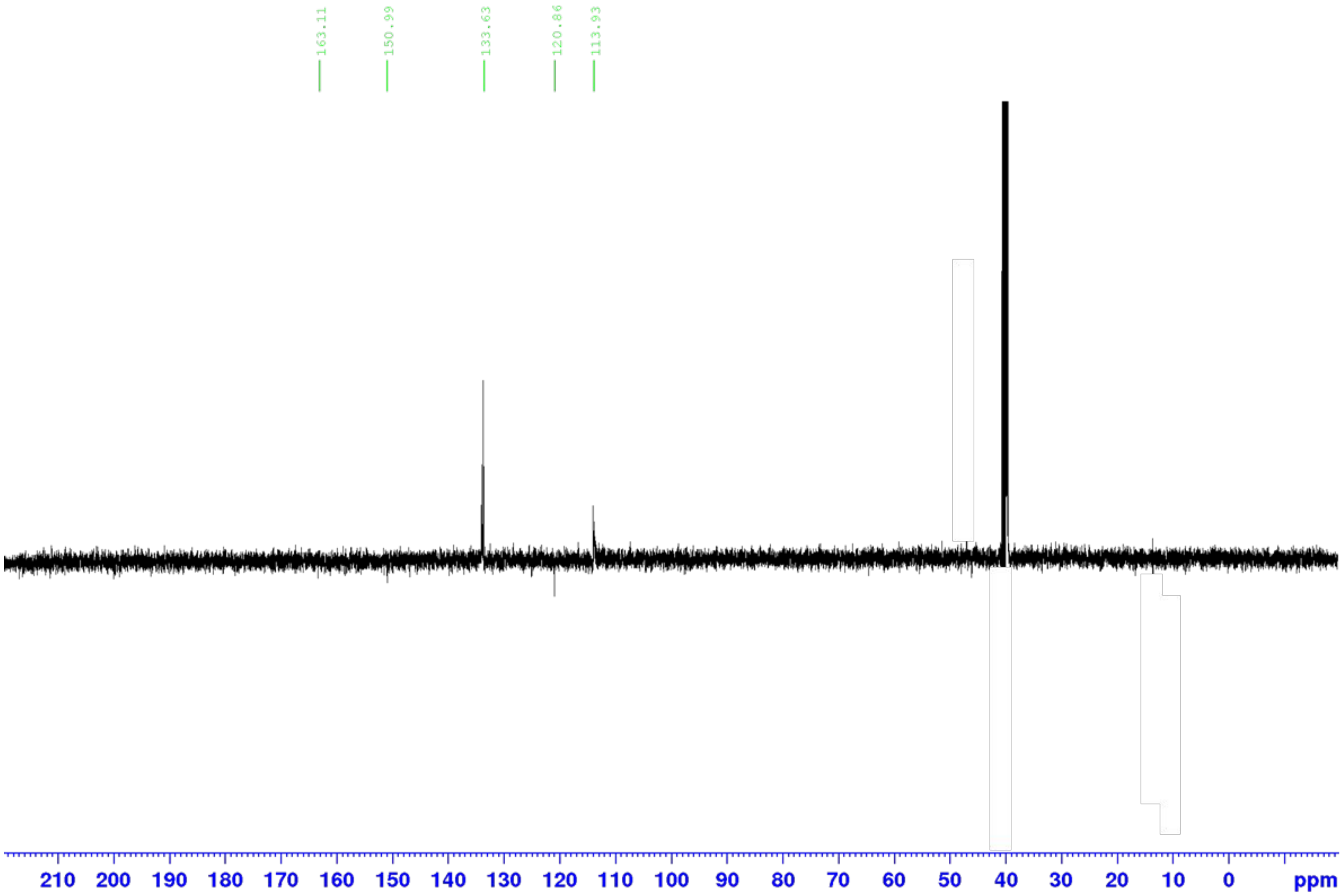

Graph S8 ^{13}C NMR spectrum of 4-(1*H*-tetrazol-5-yl)aniline.

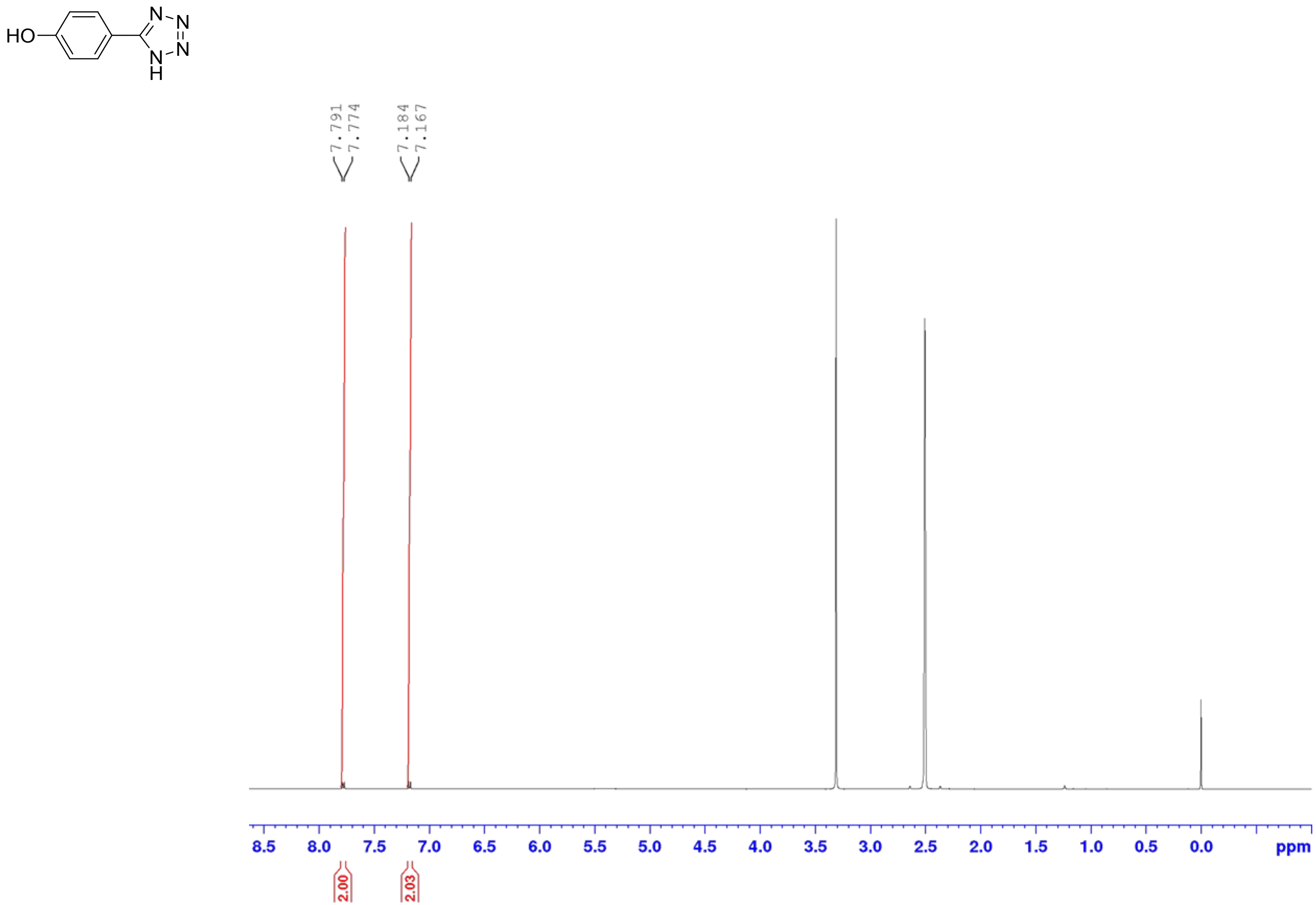

Graph S9 ^{1}H NMR spectrum of 4-(1*H*-tetrazol-5-yl)phenol.

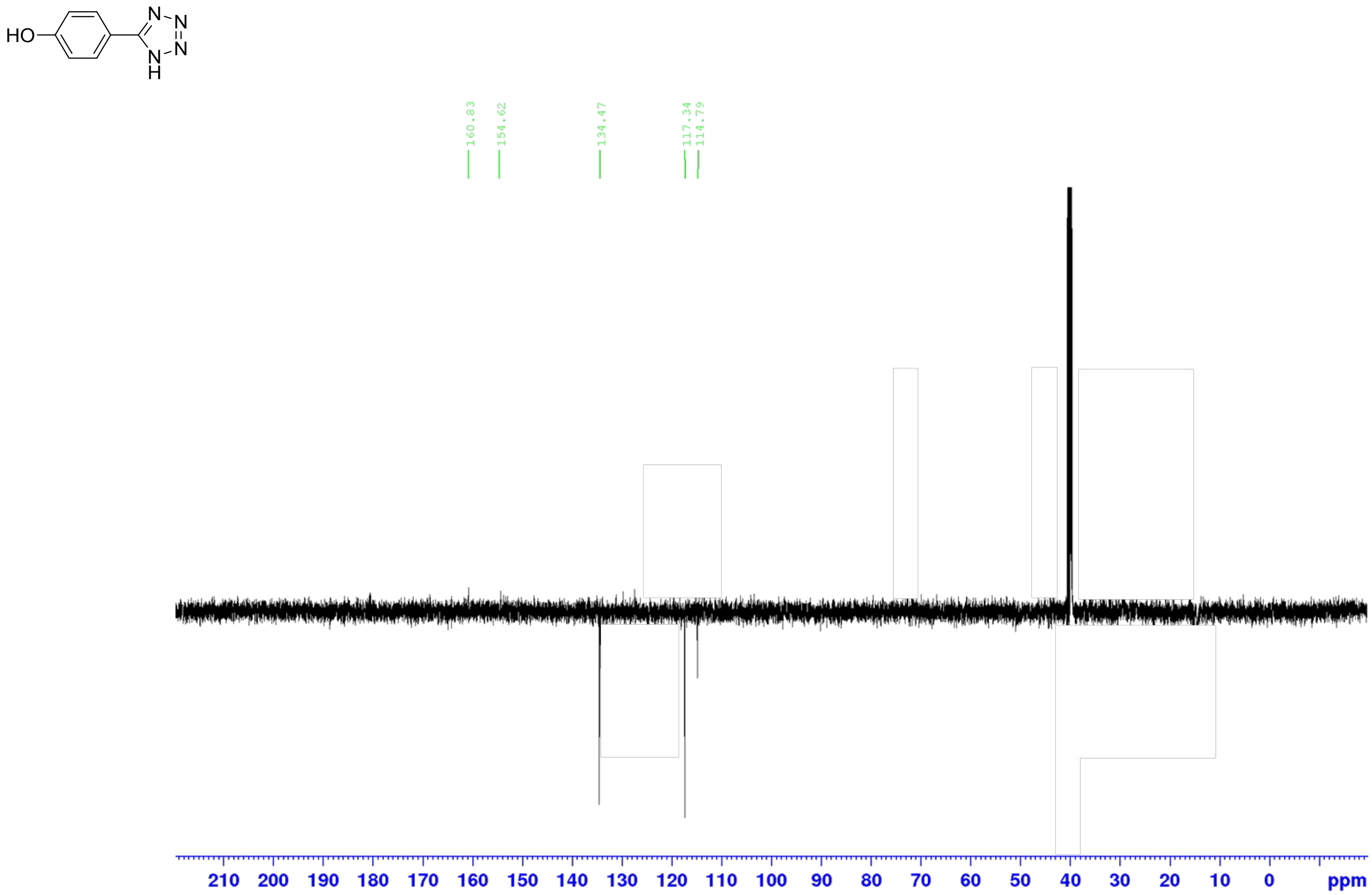

Graph S10 ^{13}C NMR spectrum of 4-(1*H*-tetrazol-5-yl)phenol.

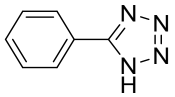

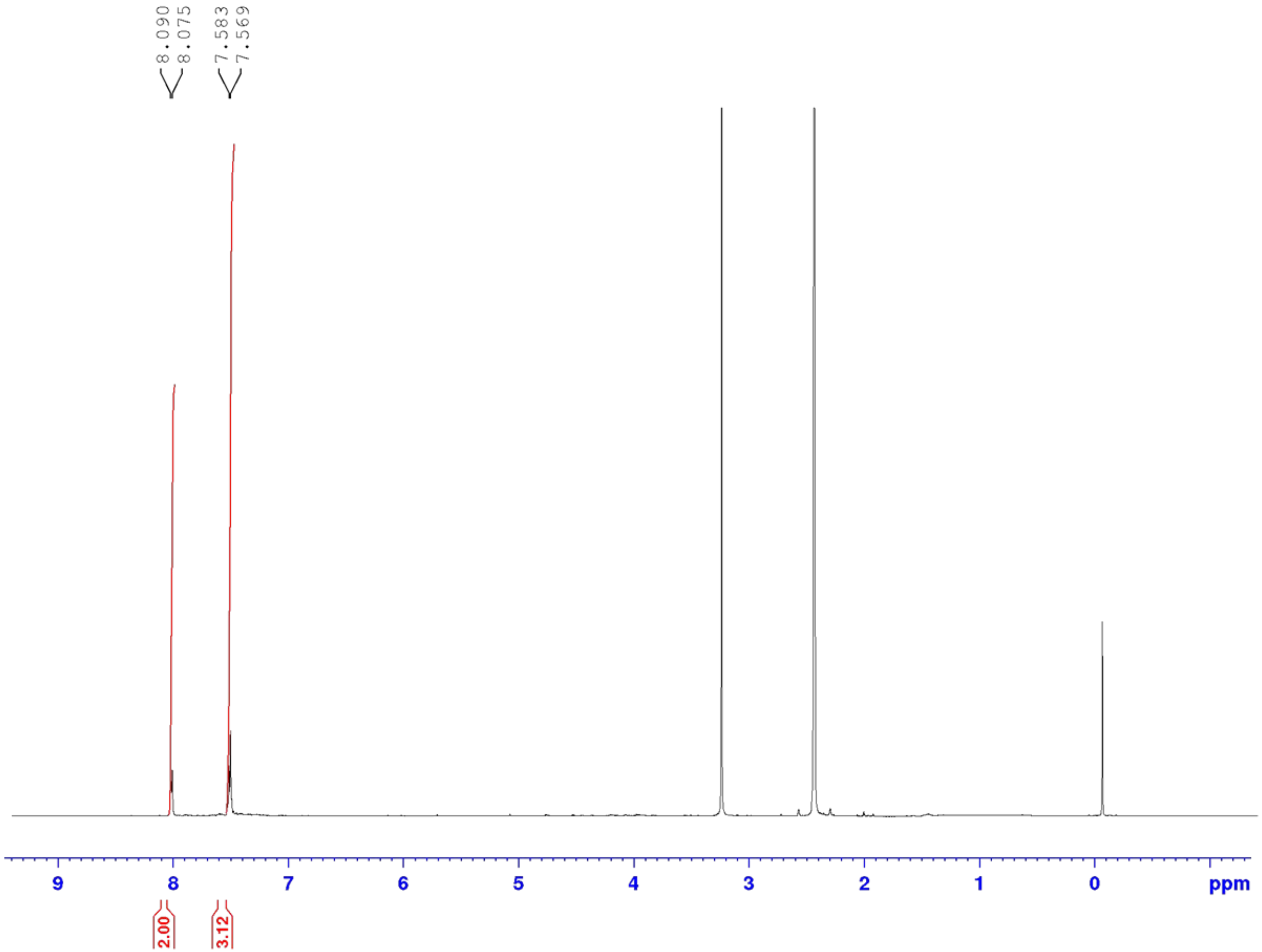

Graph S11 ^{1}H NMR spectrum of 5-phenyl-1*H*-tetrazole.

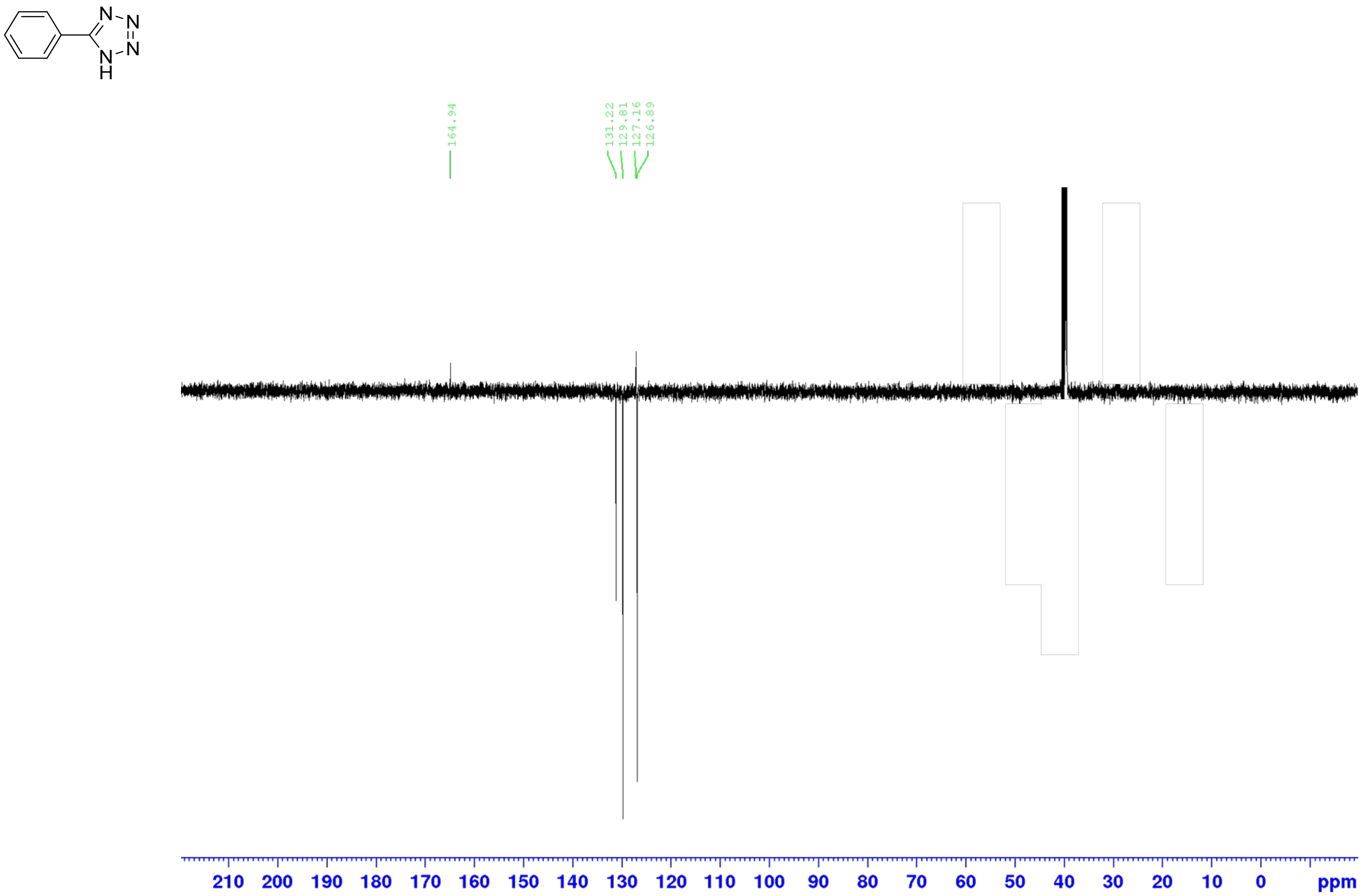

Graph S12 ^{13}C NMR spectrum of 5-phenyl-1*H*-tetrazole.

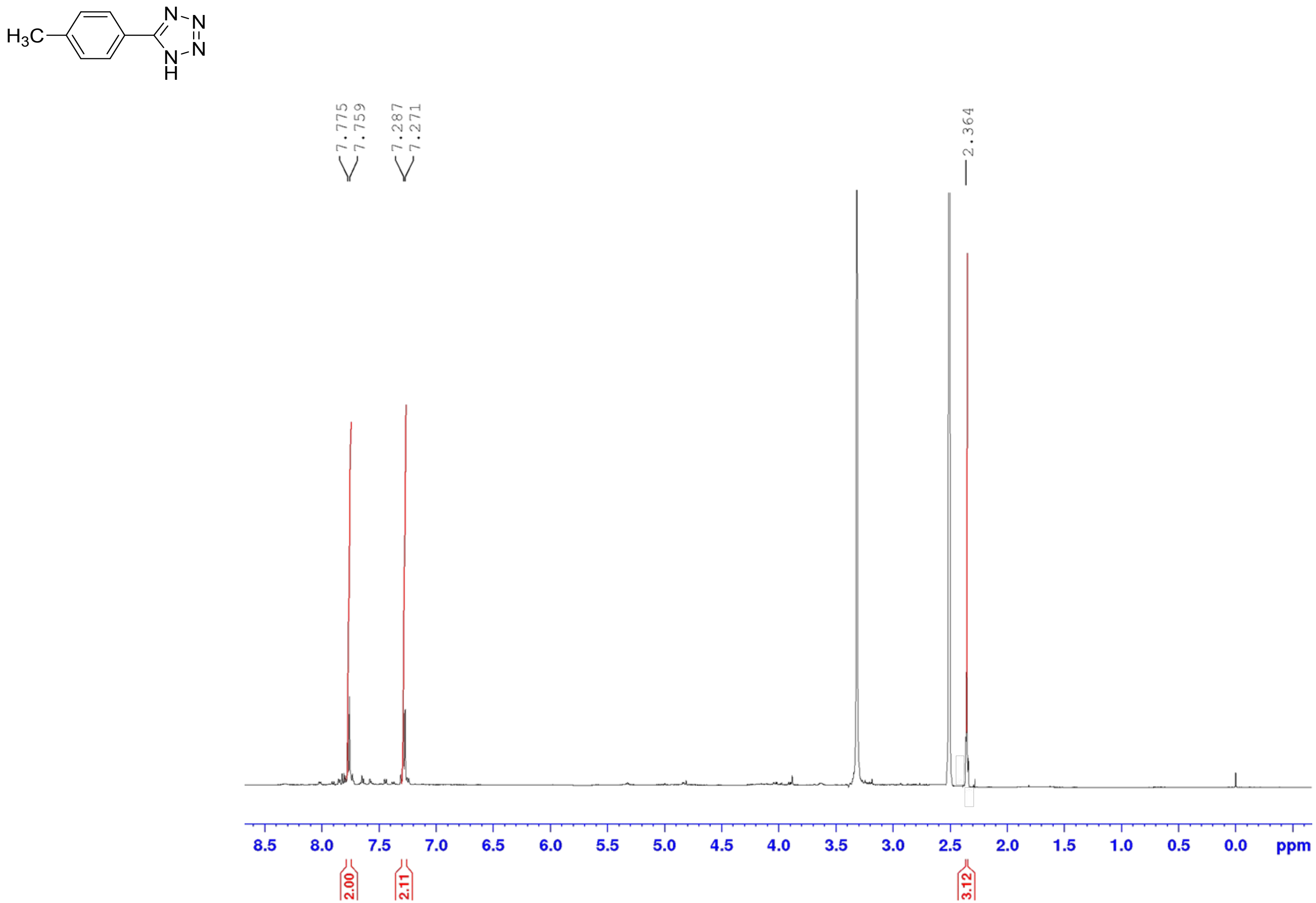

Graph S13 ^{1}H NMR spectrum of 5-(4-methylphenyl)-1*H*-tetrazole.

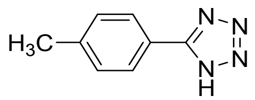

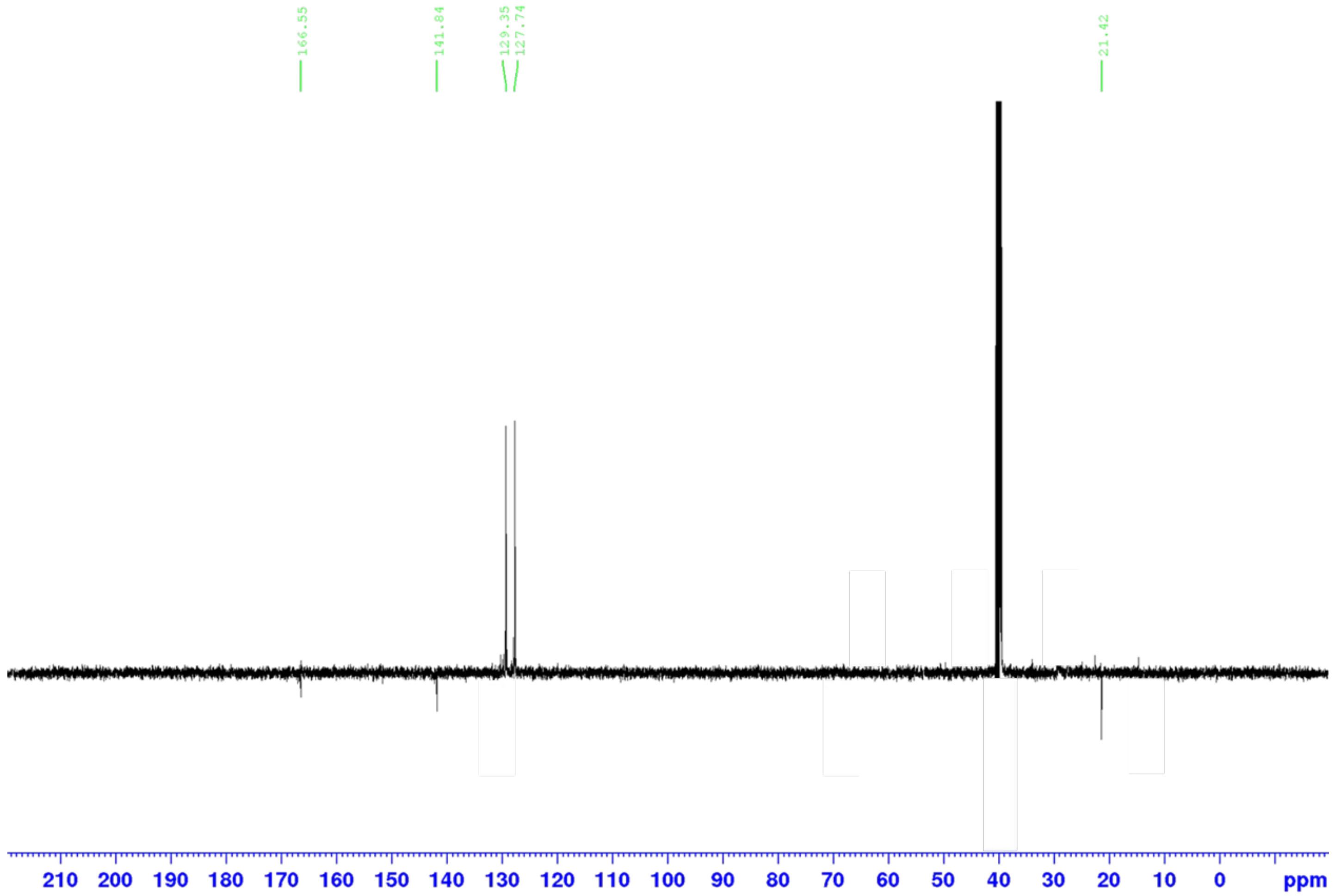

Graph S14 ^{13}C NMR spectrum of 5-(4-methylphenyl)-1*H*-tetrazole.

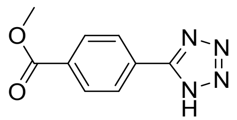

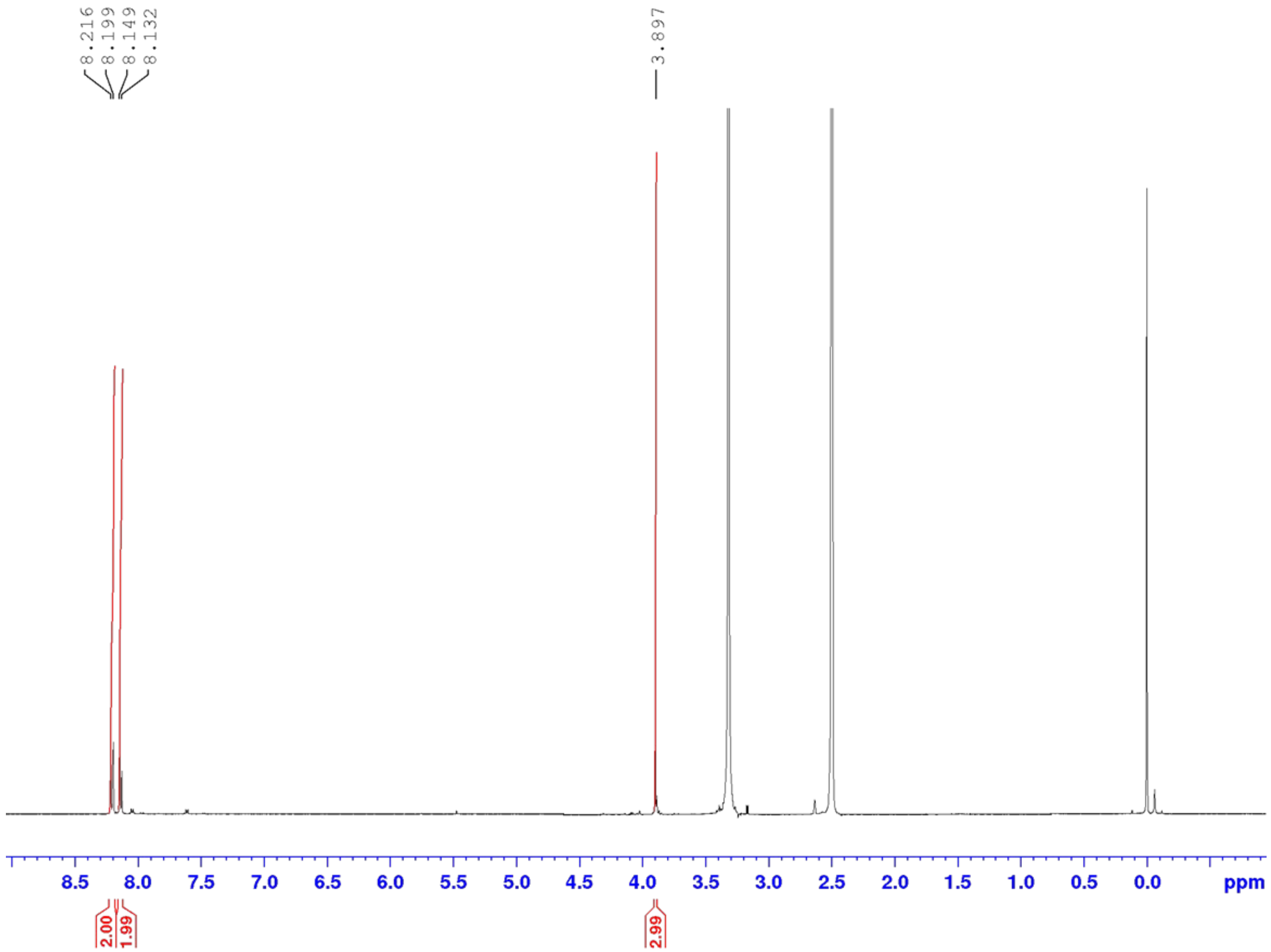

Graph S15 ^{1}H NMR spectrum of methyl 4-(1*H*-tetrazol-5-yl)benzoate.

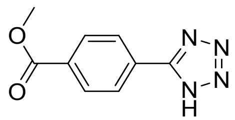

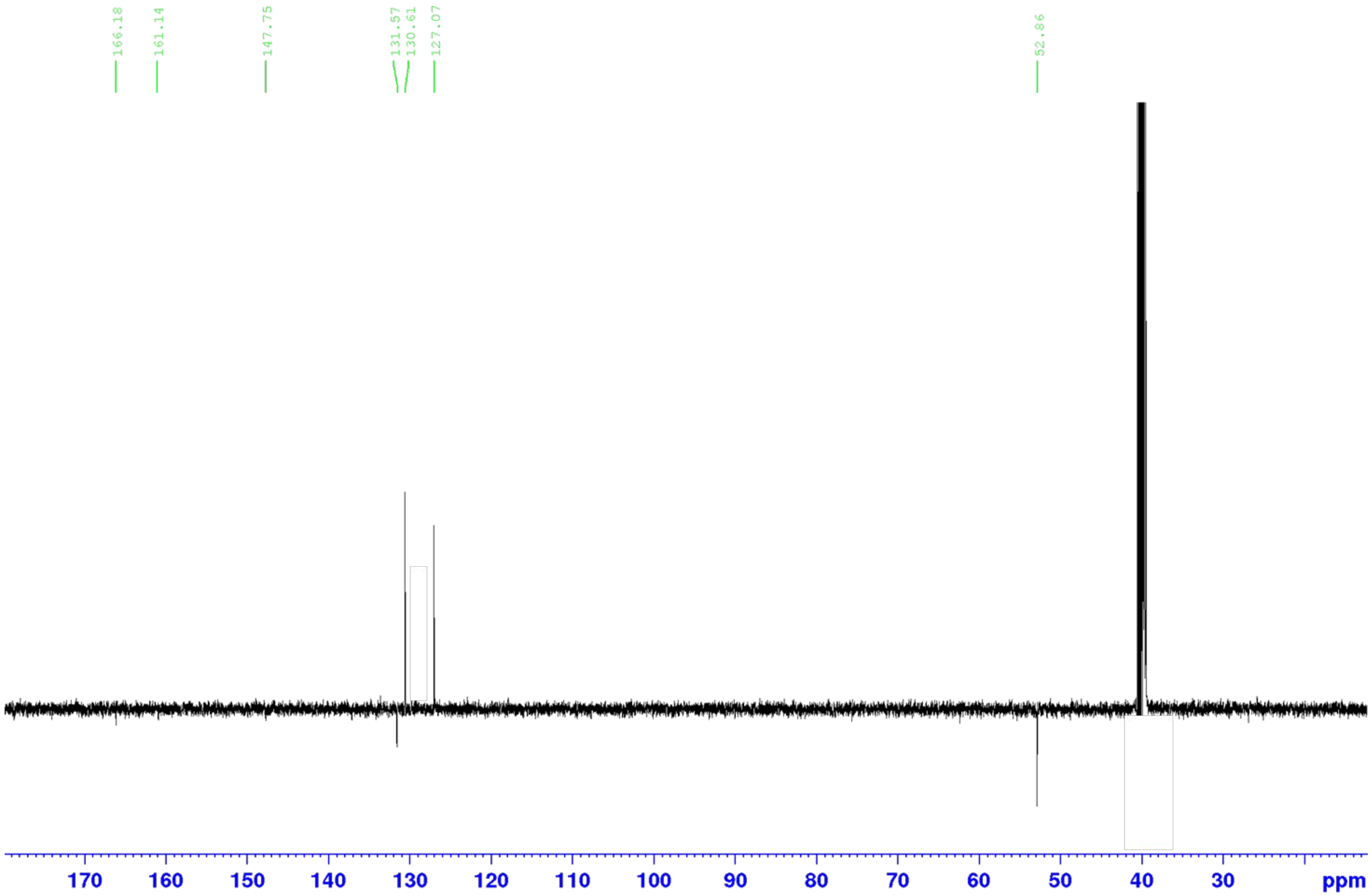

Graph S16 ^{13}C NMR spectrum of methyl 4-(1*H*-tetrazol-5-yl)benzoate.

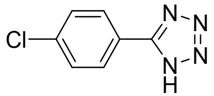

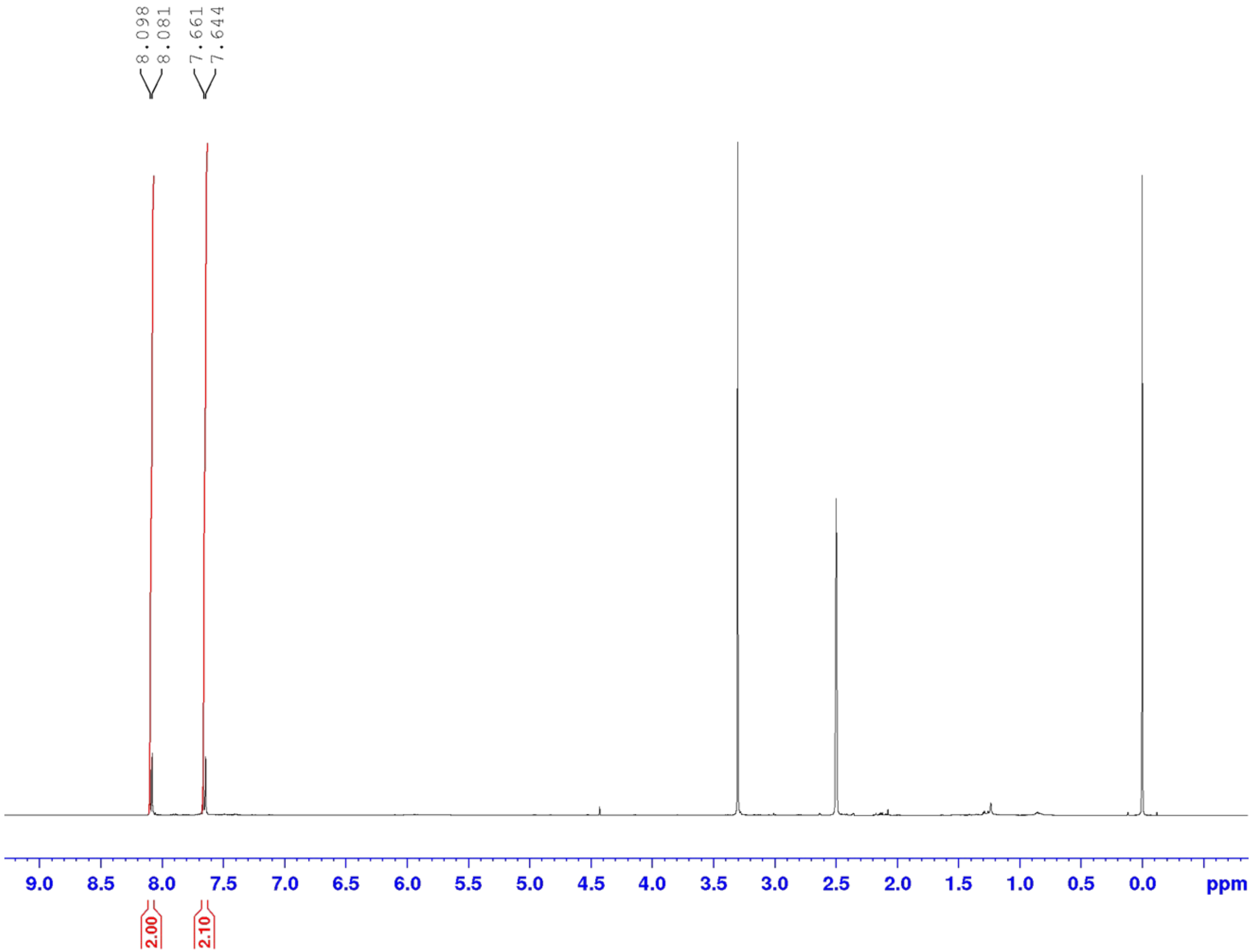

Graph S17 ^{1}H NMR spectrum of 5-(4-chlorophenyl)-1*H*-tetrazole.

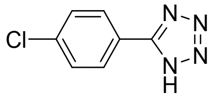

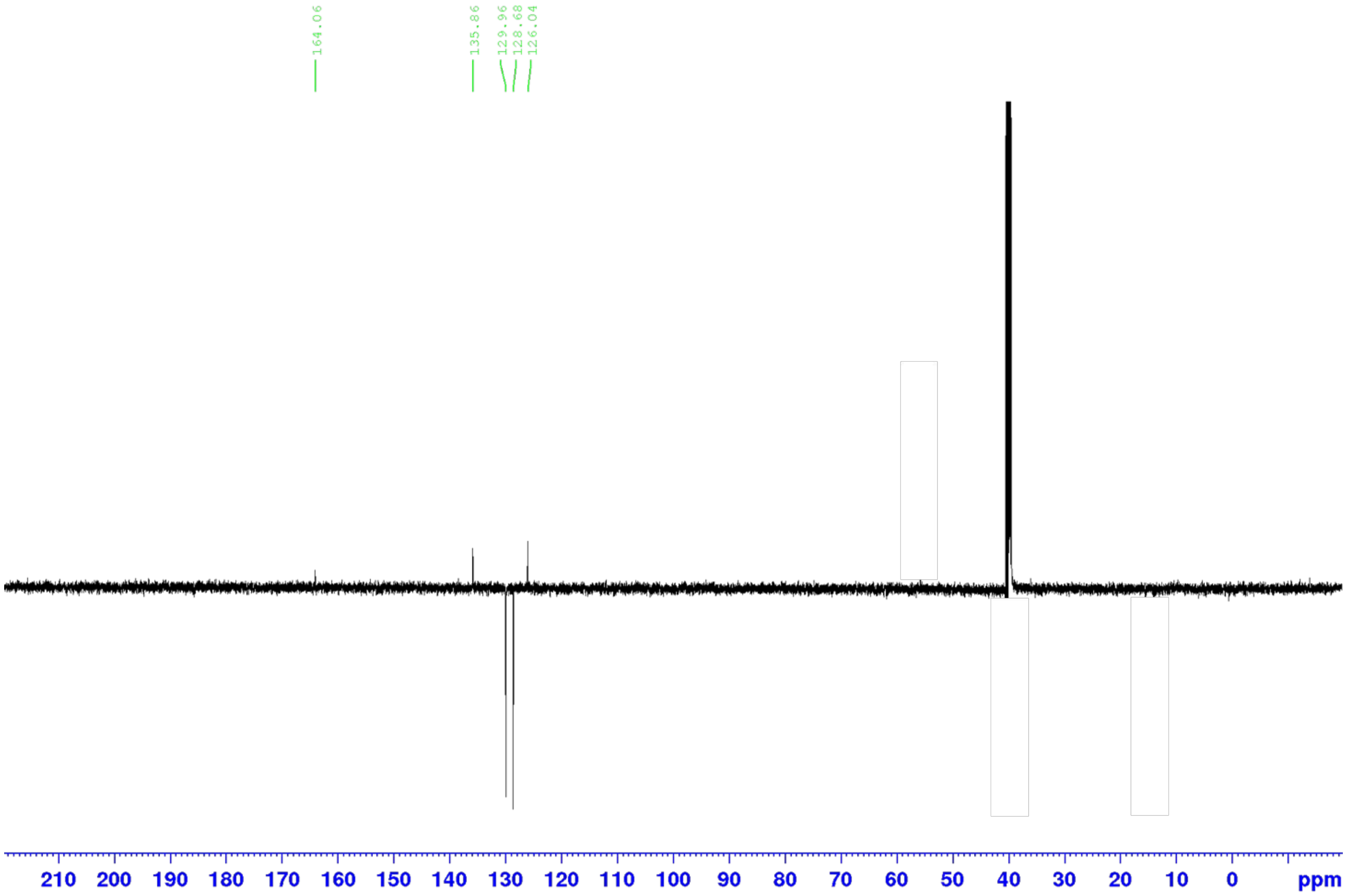

Graph S18 ^{13}C NMR spectrum of 5-(4-chlorophenyl)-1*H*-tetrazole.

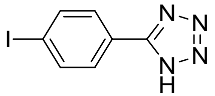

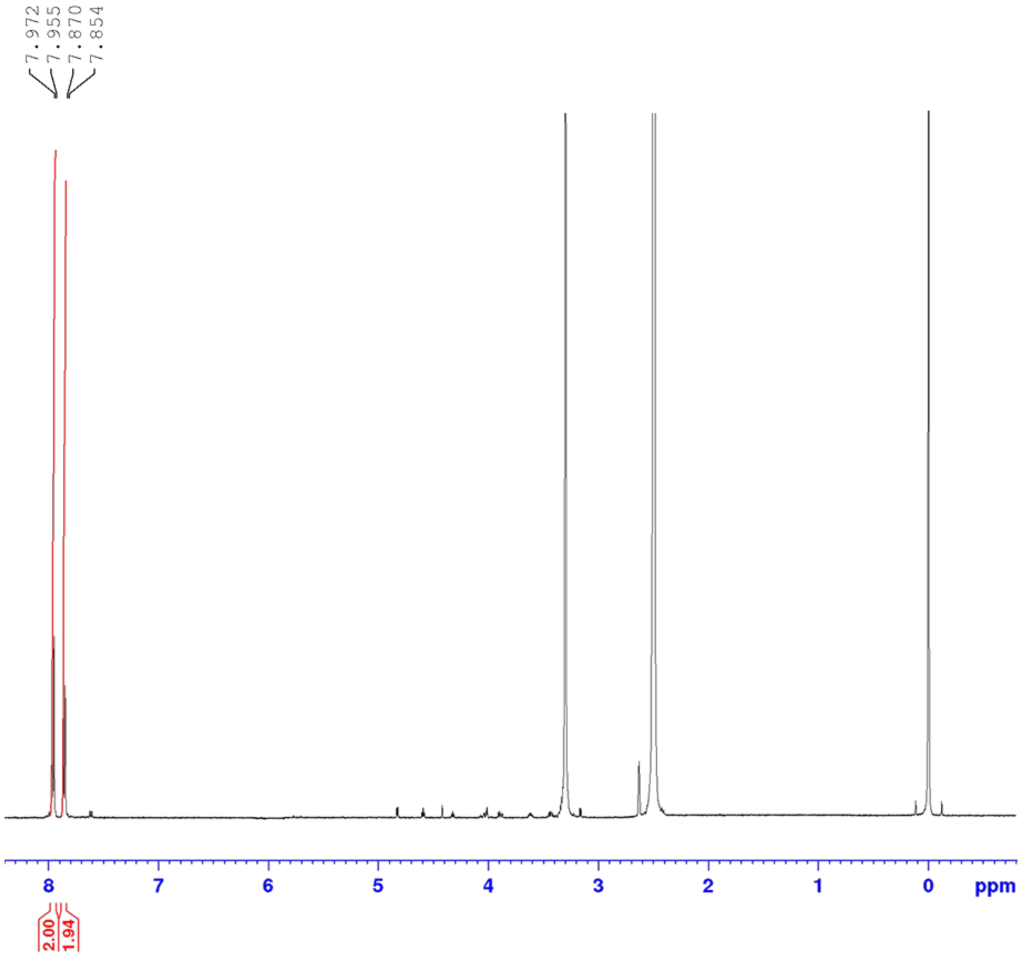

Graph S19 ^{1}H NMR spectrum of 5-(4-iodophenyl)-1*H*-tetrazole.

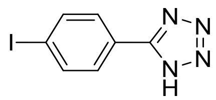

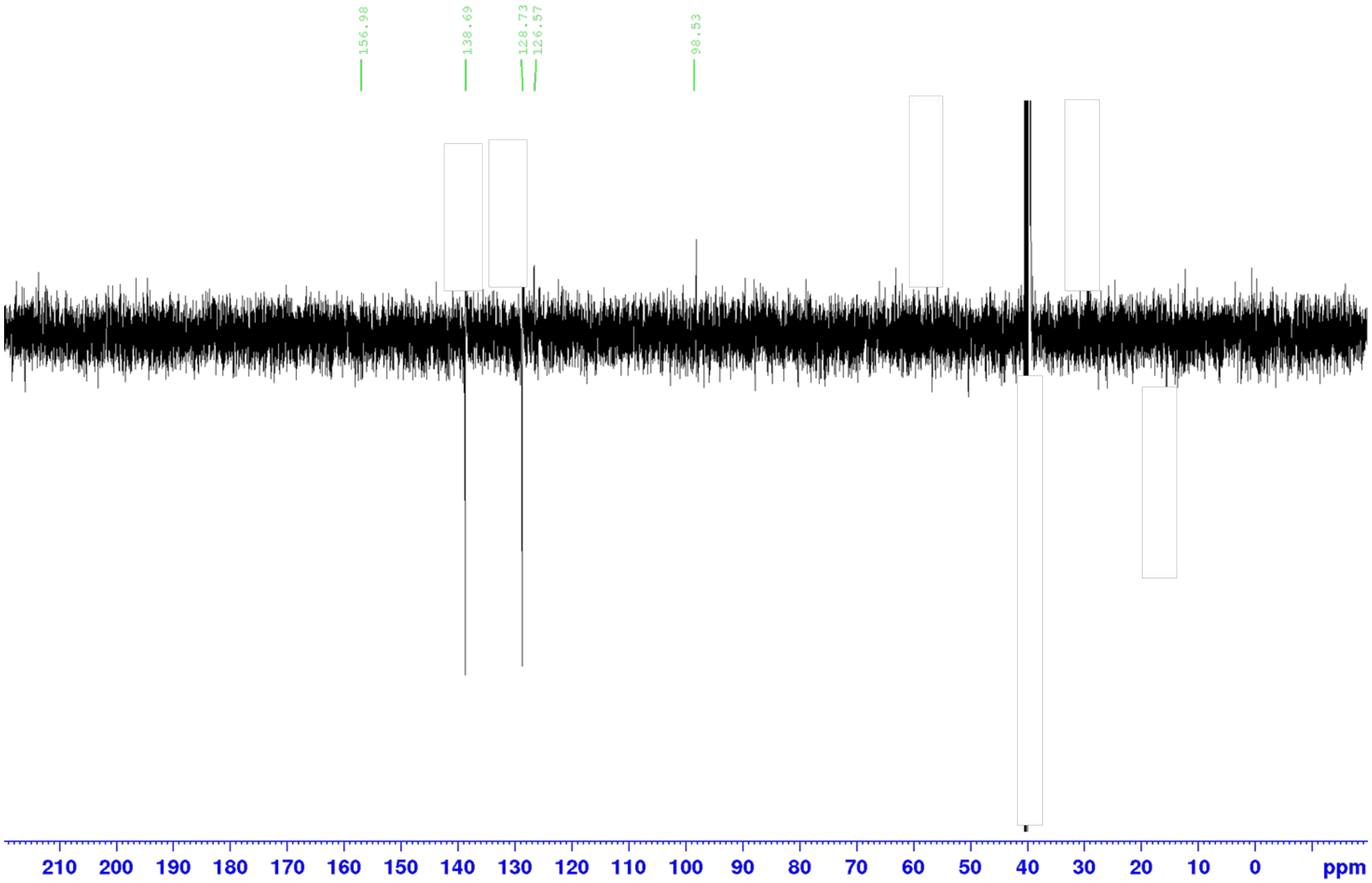

Graph S20 ^{13}C NMR spectrum of 5-(4-iodophenyl)-1*H*-tetrazole.

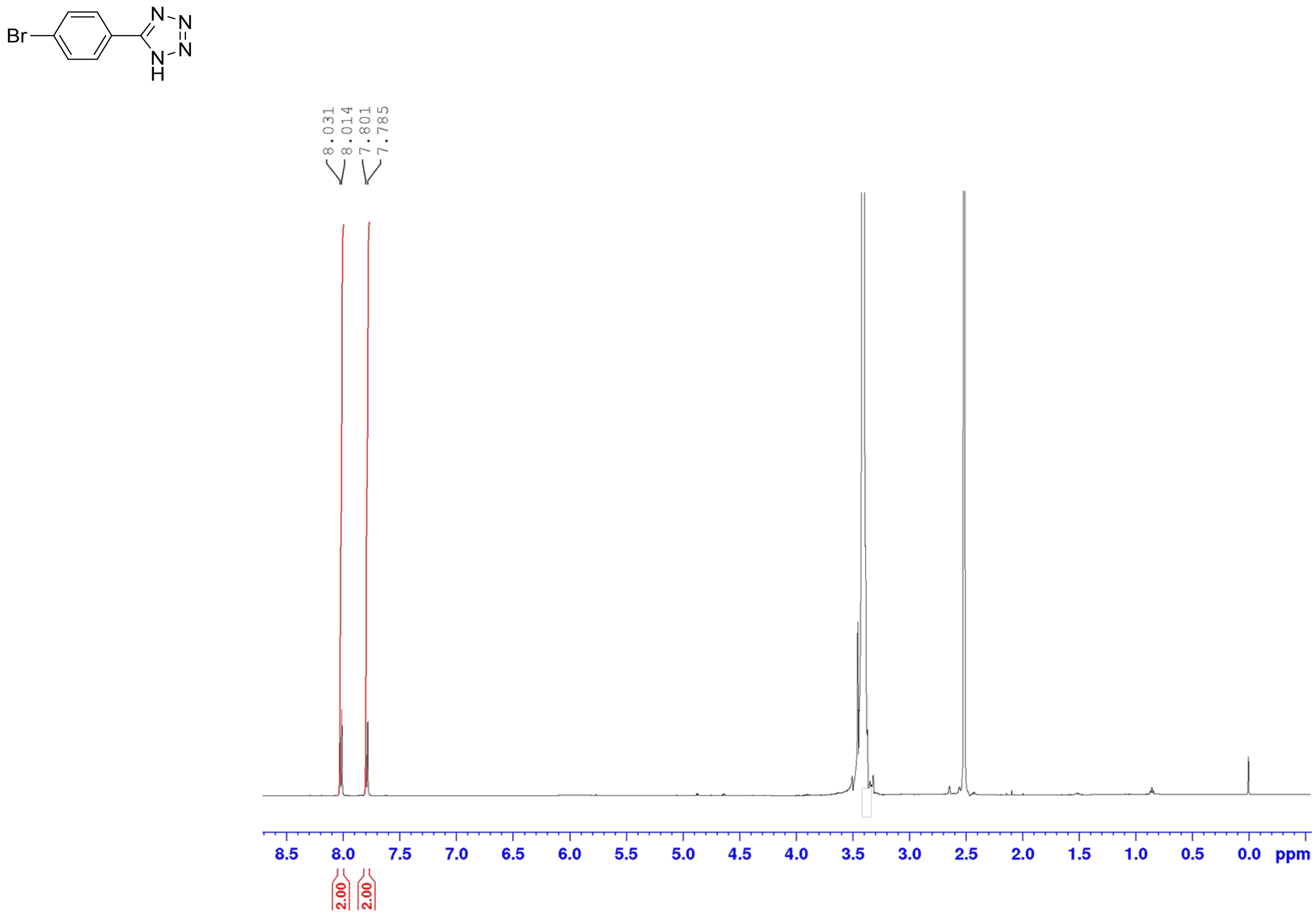

Graph S21 ^{1}H NMR spectrum of 5-(4-bromophenyl)-1*H*-tetrazole.

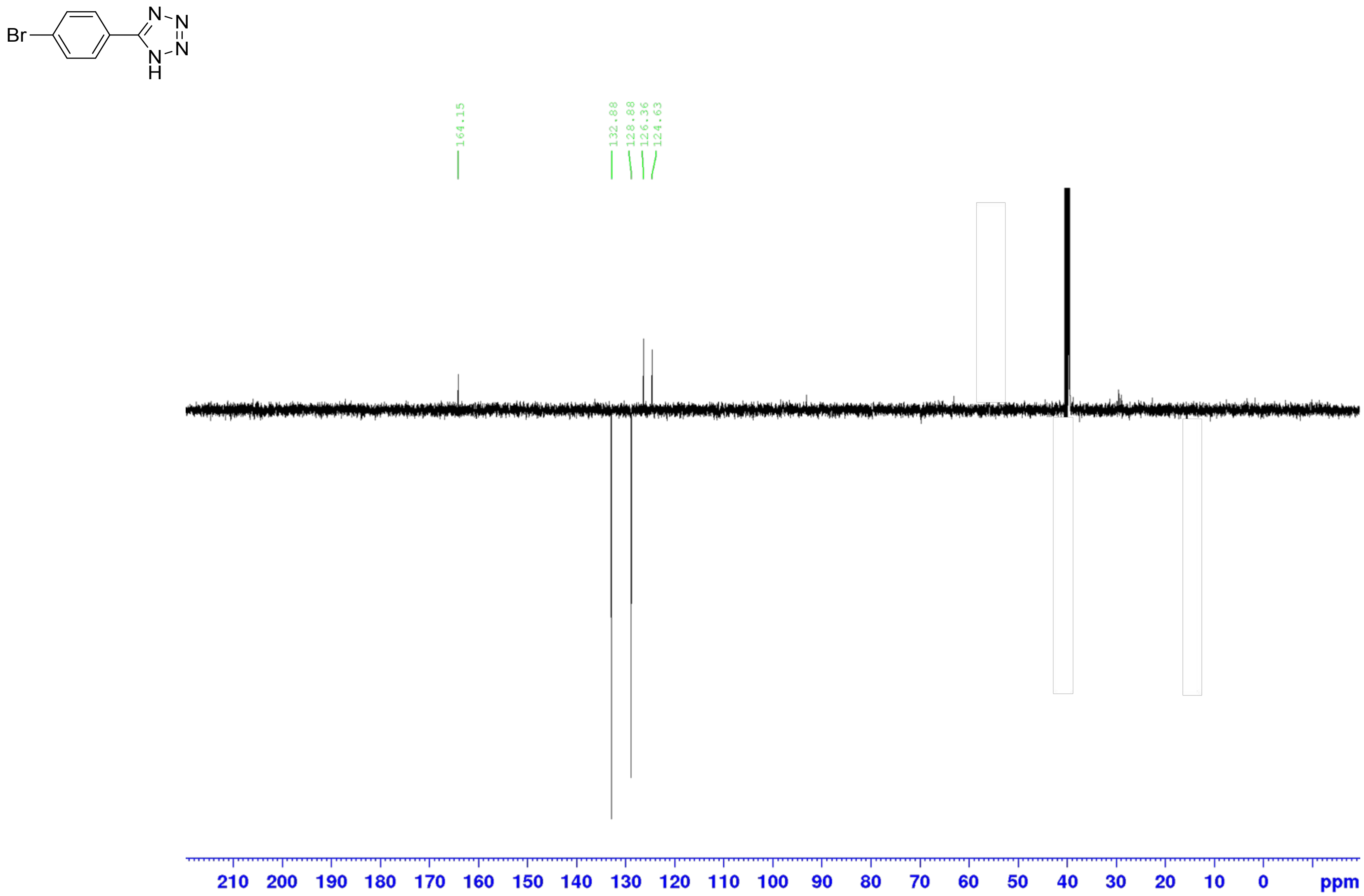

Graph S22 ^{13}C NMR spectrum of 5-(4-bromophenyl)-1*H*-tetrazole.

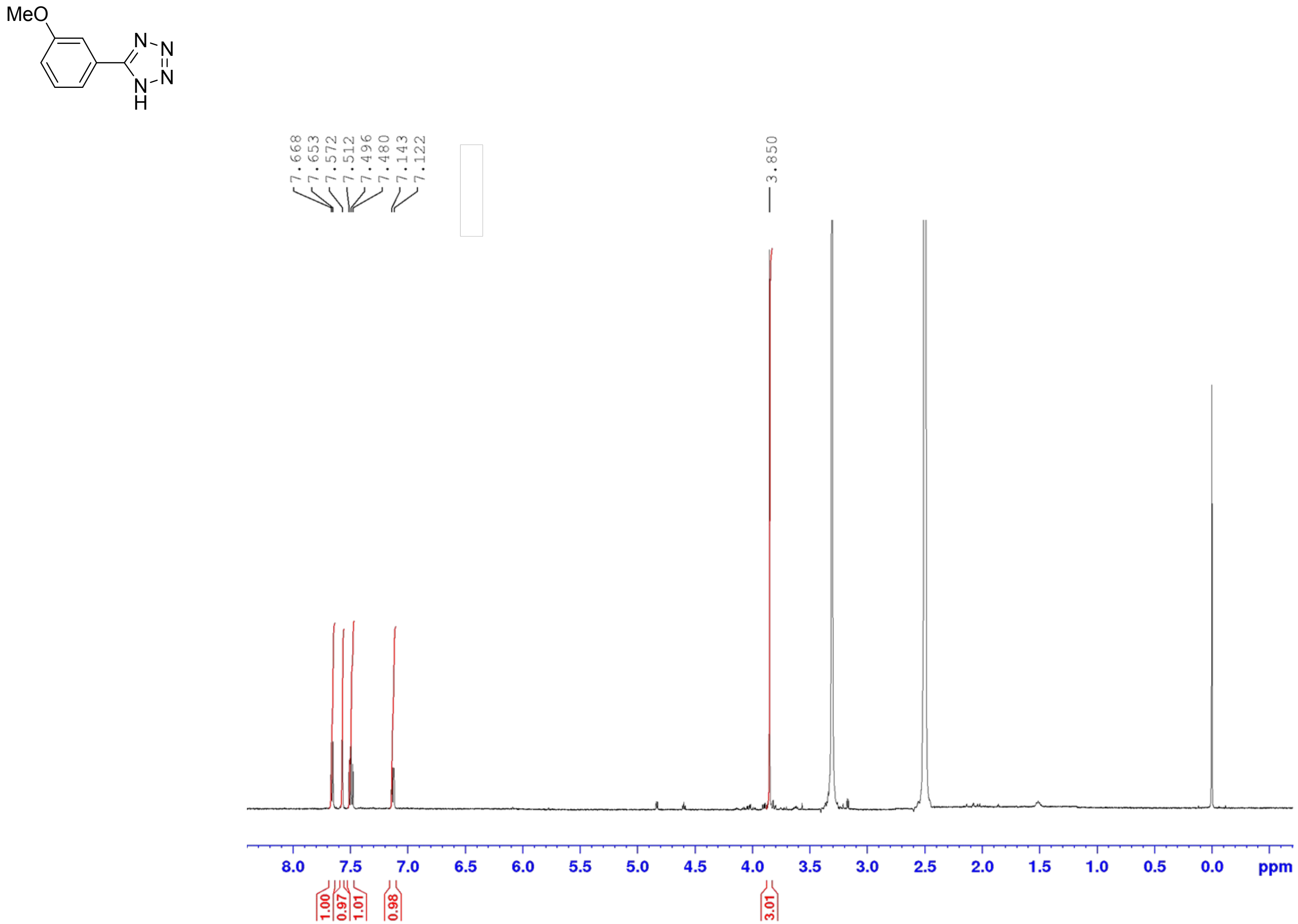

Graph S23 ^{1}H NMR spectrum of 5-(3-methoxyphenyl)-1*H*-tetrazole.

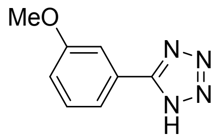

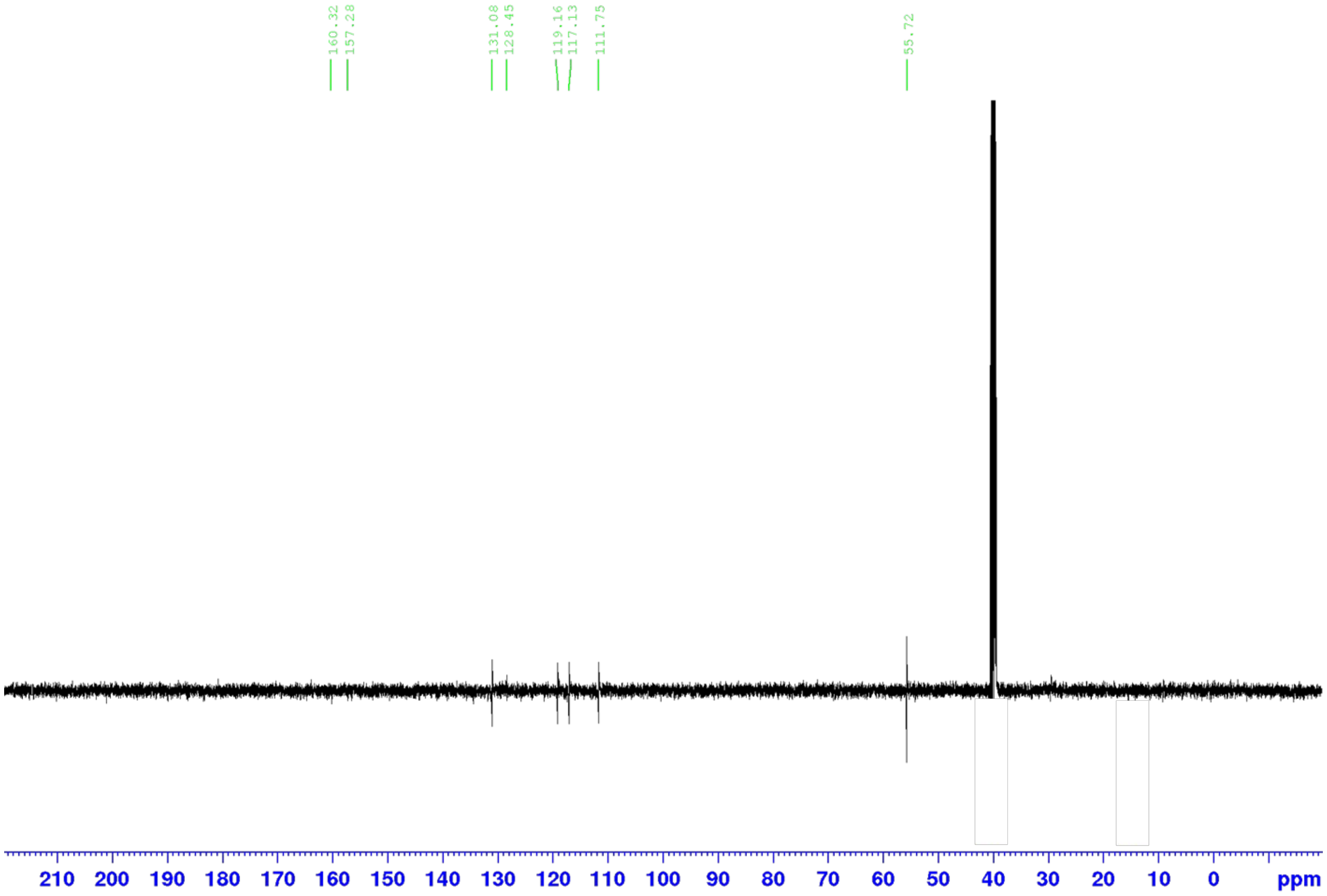

Graph S24 ^{13}C NMR spectrum of 5-(3-methoxyphenyl)-1*H*-tetrazole.

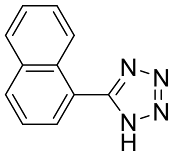

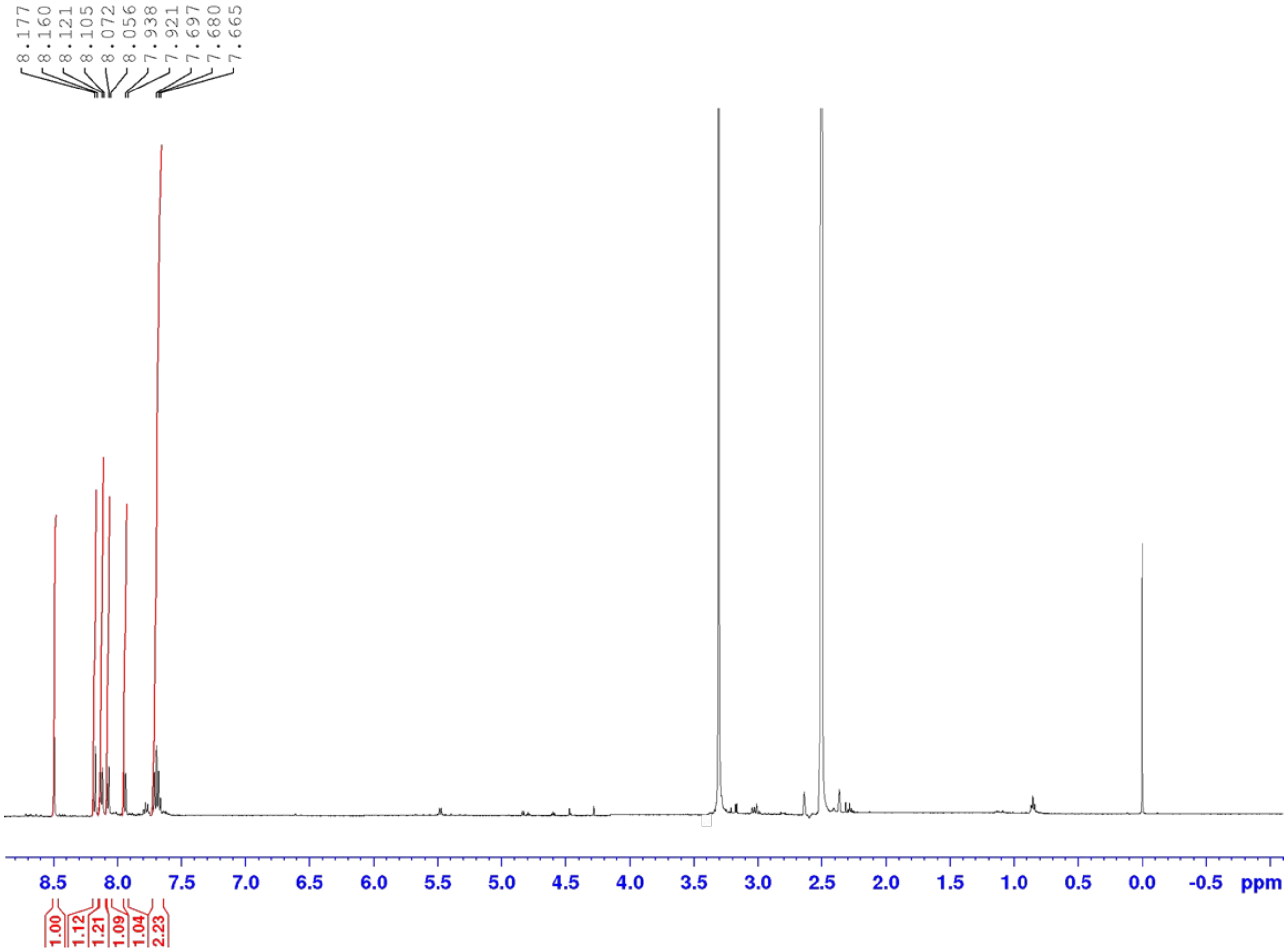

Graph S25 ^{1}H NMR spectrum of 5-(naphthalen-2-yl)-1*H*-tetrazole.

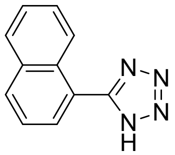

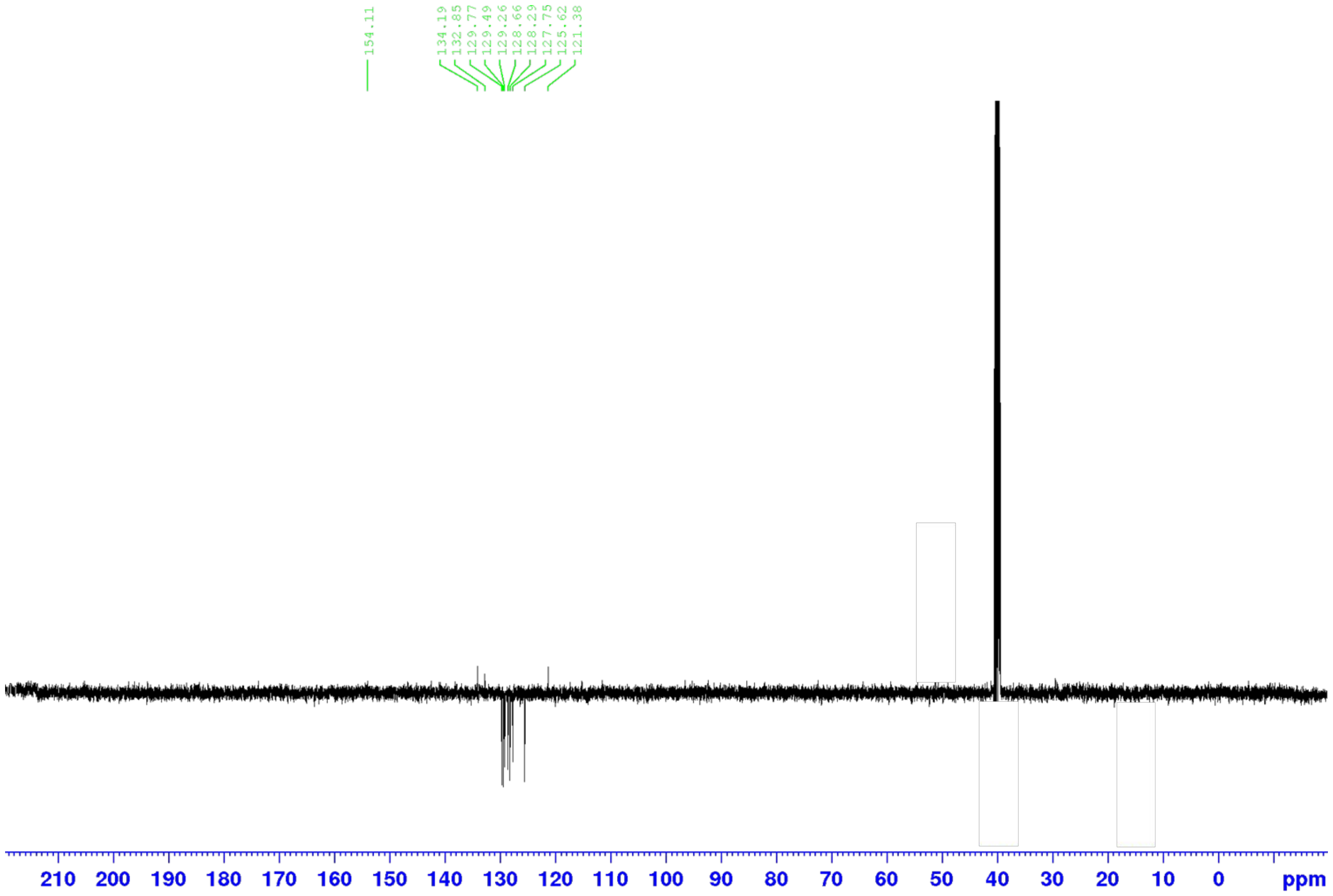

Graph S26 ^{13}C NMR spectrum of 5-(naphthalen-2-yl)-1*H*-tetrazole.

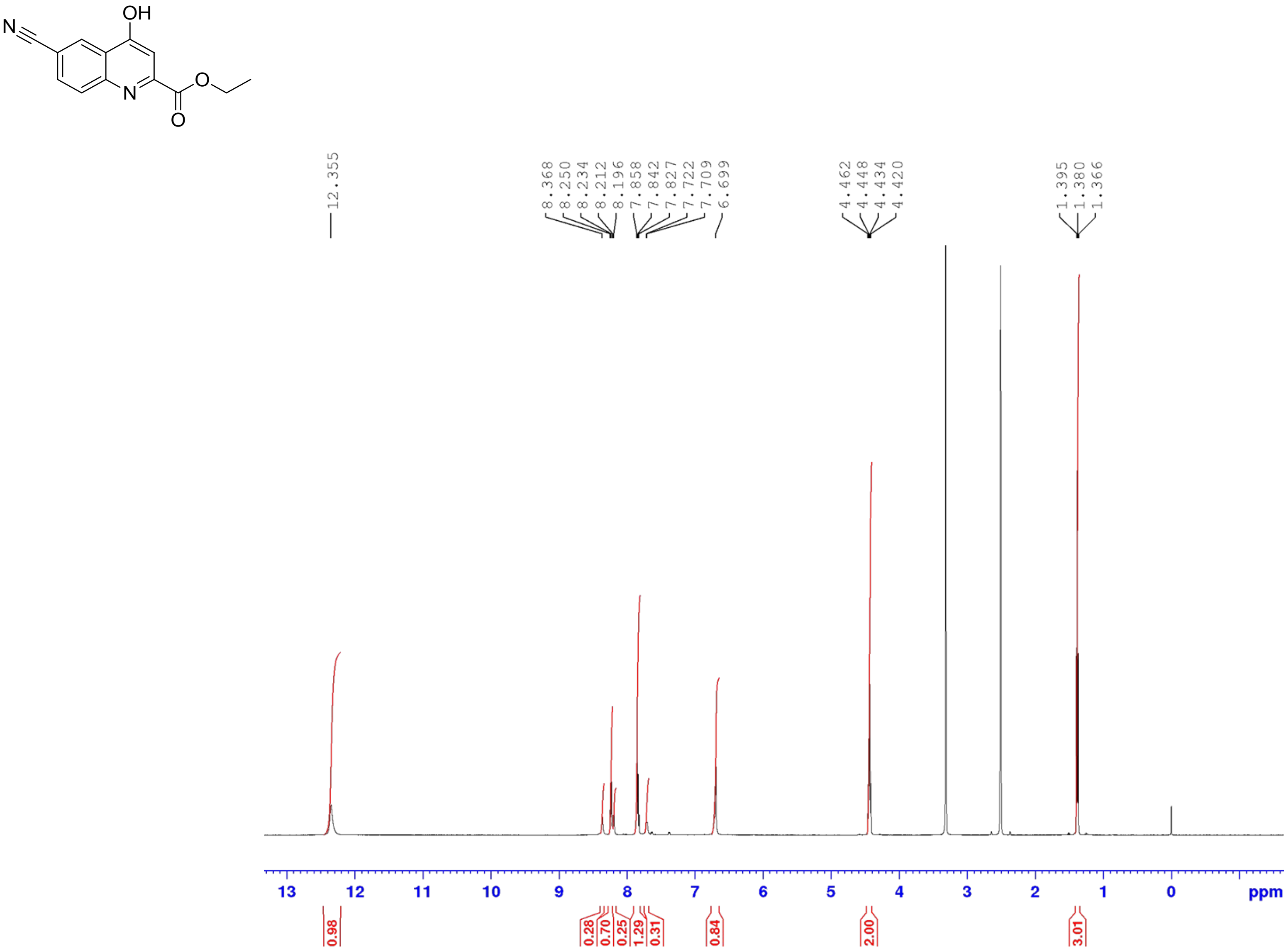

Graph S27 ^{1}H NMR spectrum of ethyl 6-cyano-4-hydroxy-2-quinolinecarboxylate.

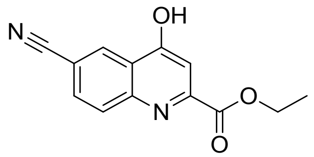

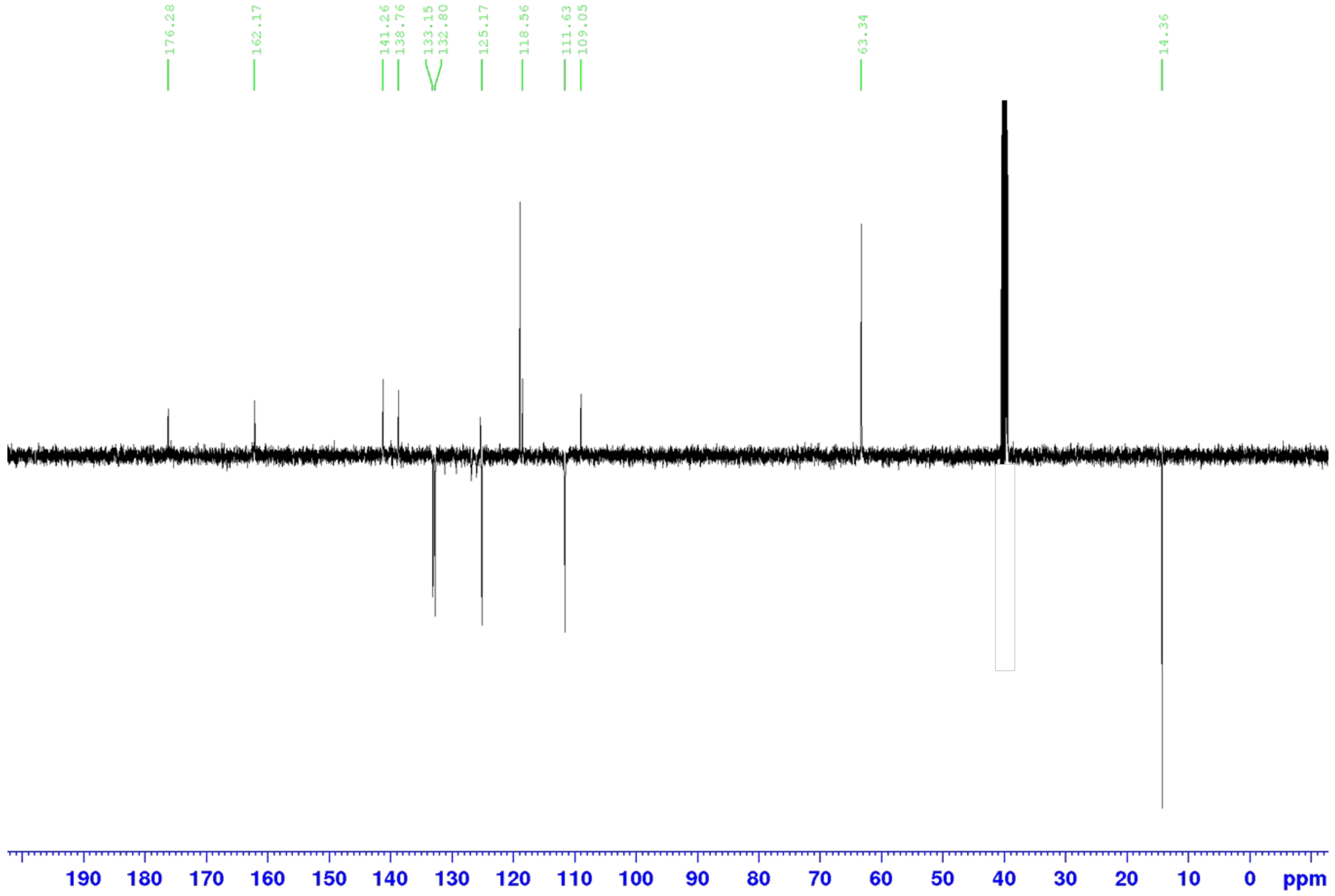

Graph S28 ^{13}C NMR spectrum of ethyl 6-cyano-4-hydroxy-2-quinolinecarboxylate.

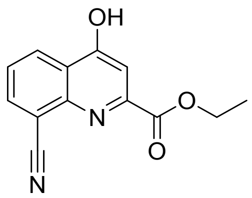

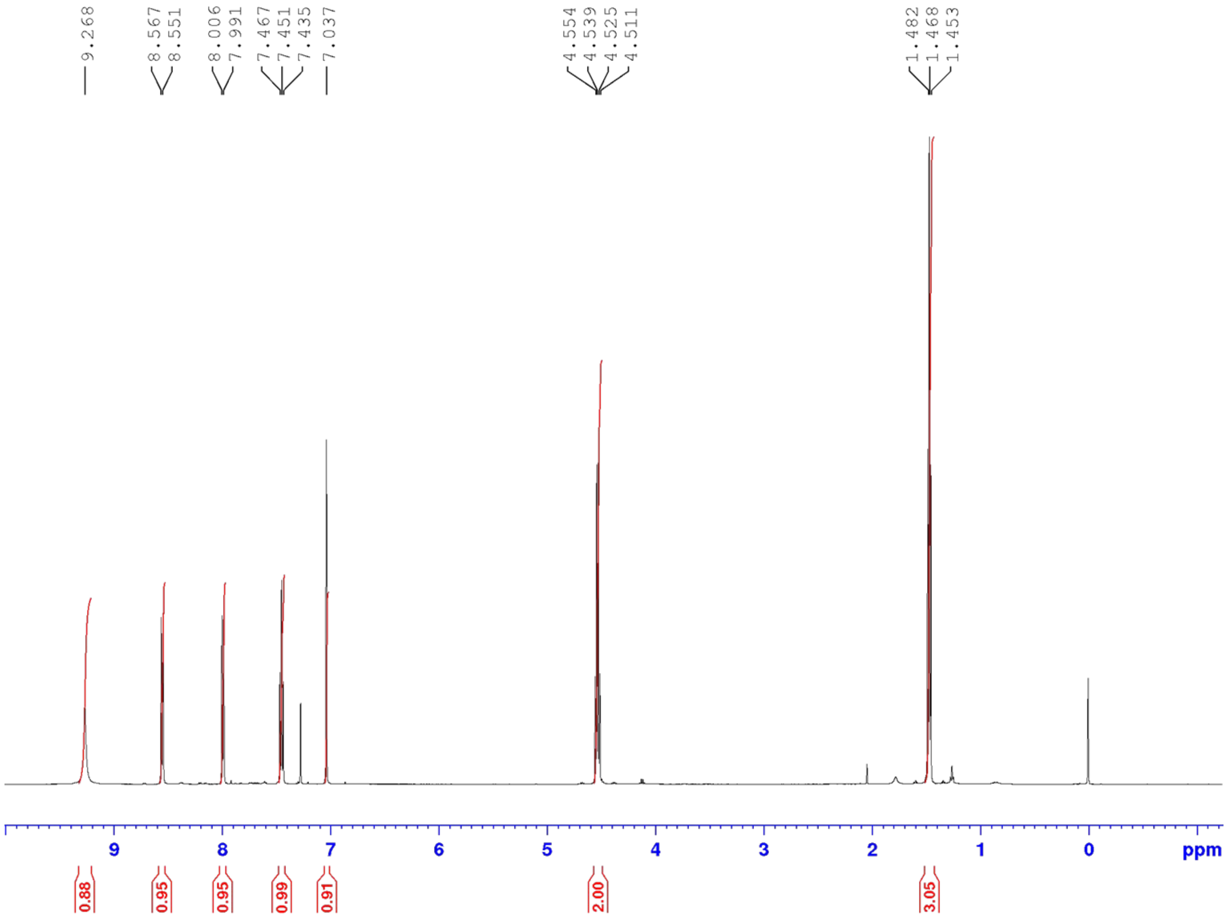

Graph S29 ^{1}H NMR spectrum of ethyl 8-cyano-4-hydroxy-2-quinolinecarboxylate.

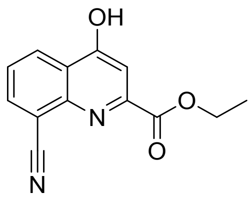

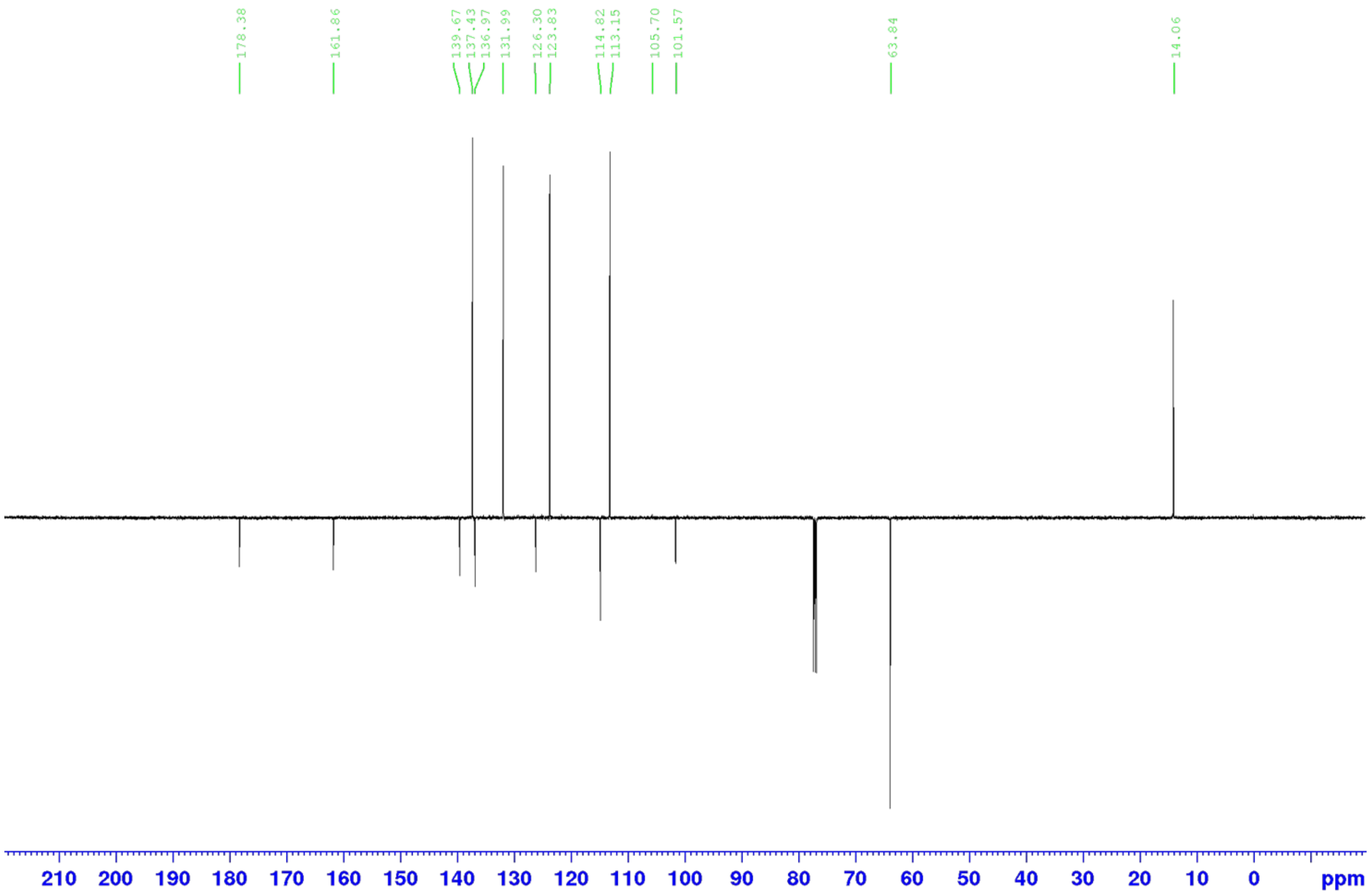

Graph S30 ^{13}C NMR spectrum of ethyl 8-cyano-4-hydroxy-2-quinolinecarboxylate.

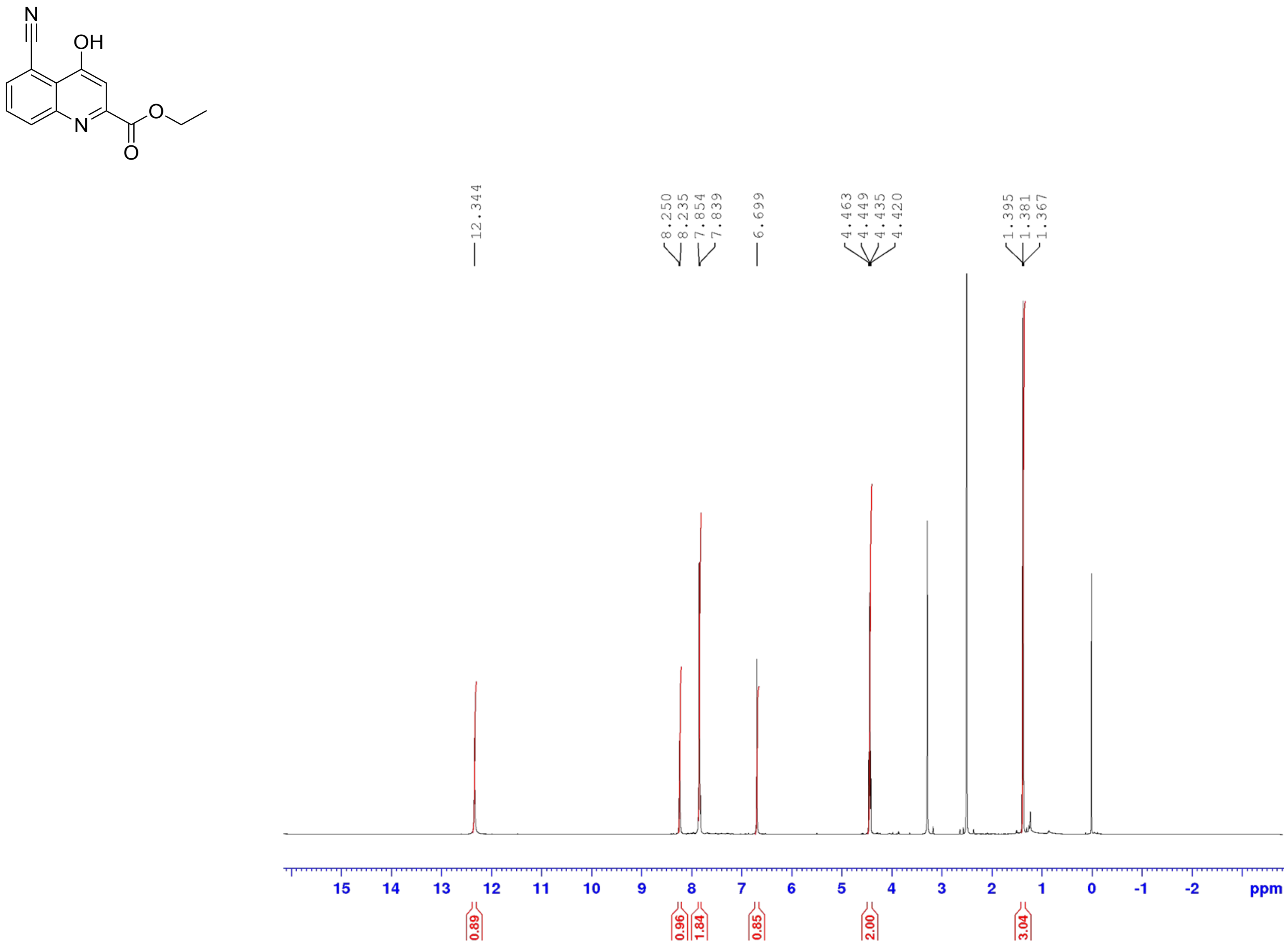

Graph S31 ^{1}H NMR spectrum of ethyl 5-cyano-4-hydroxy-2-quinolinecarboxylate.

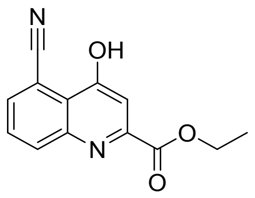

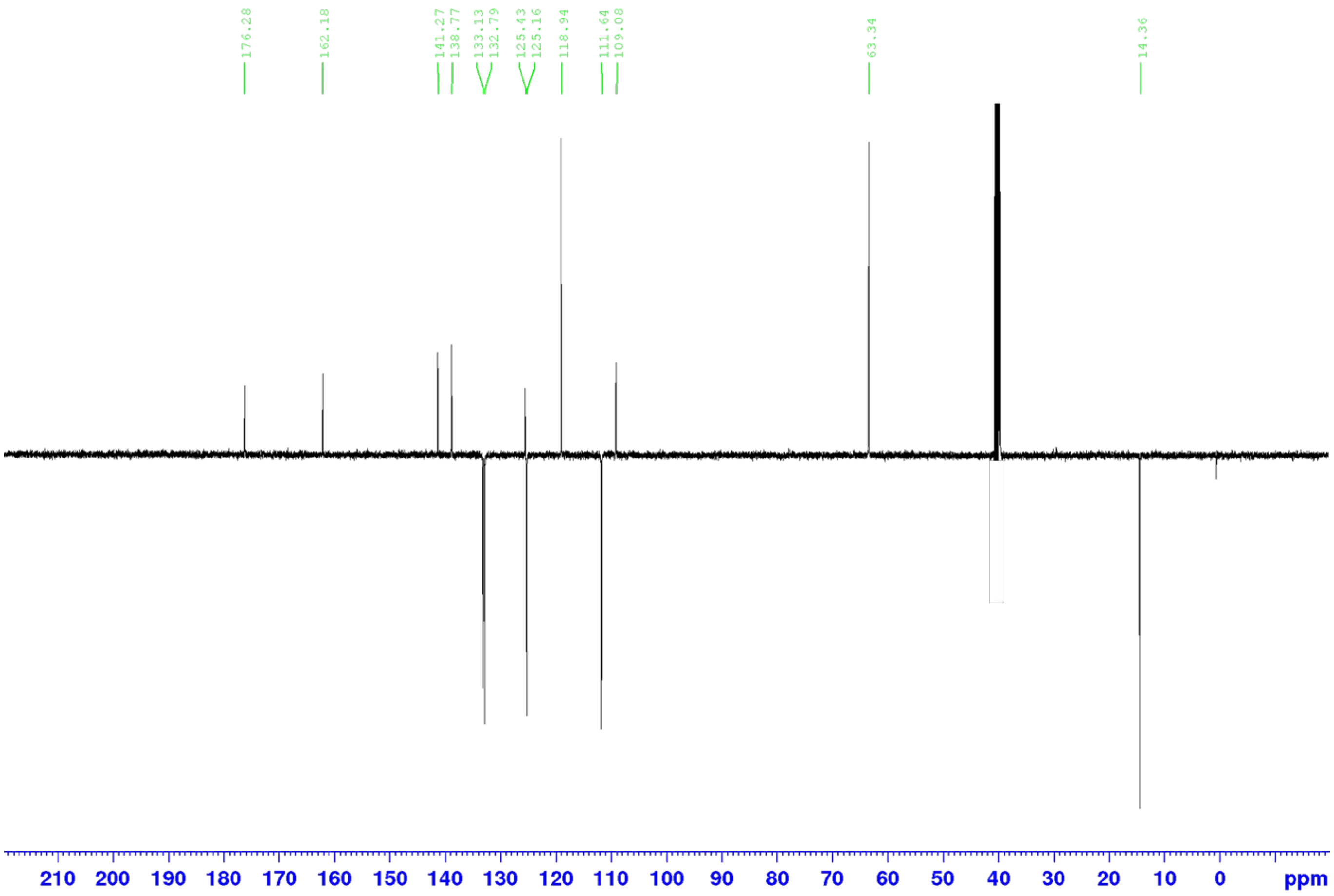

Graph S32 ^{13}C NMR spectrum of ethyl 5-cyano-4-hydroxy-2-quinolinecarboxylate.

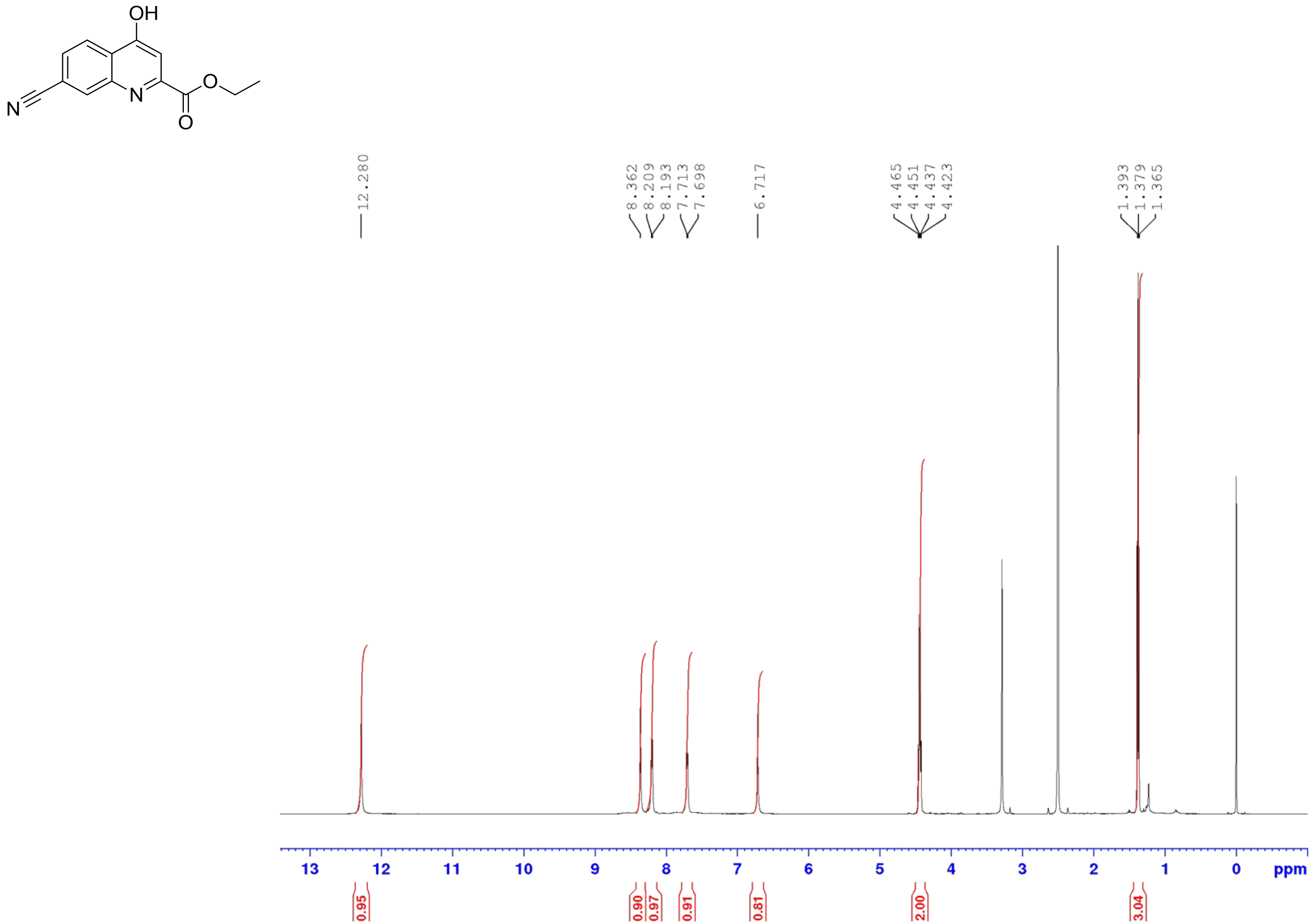

Graph S33 ^{1}H NMR spectrum of ethyl 7-cyano-4-hydroxy-2-quinolinecarboxylate.

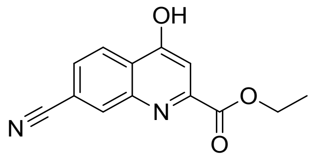

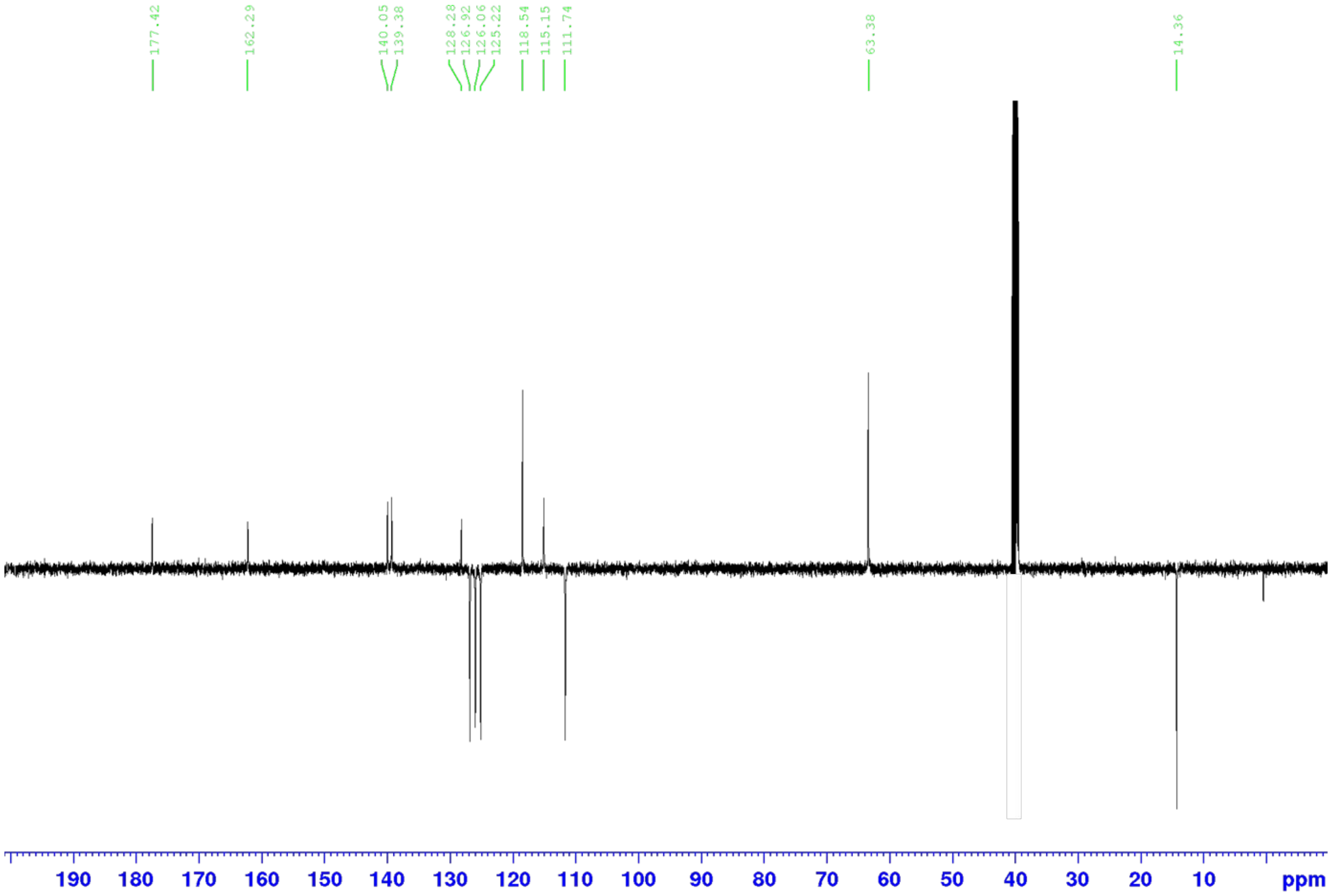

Graph S34 ^{13}C NMR spectrum of ethyl 7-cyano-4-hydroxy-2-quinolinecarboxylate.

3. References

(1) Palde, P. B.; Jamison, T. F. Safe and Efficient Tetrazole Synthesis in a Continuous-Flow Microreactor. *Angew. Chem.* **2011**, *123* (15), 3587-3590.

(2) Erken, E.; Esirden, İ.; Kaya, M.; Sen, F. Retracted Article: A rapid and novel method for the synthesis of 5-substituted 1 H-tetrazole catalyzed by exceptional reusable monodisperse Pt NPs@ AC under the microwave irradiation. *RSC Adv.* **2015**, *5* (84), 68558-68564.

(3) Xie, A.; Cao, M.; Liu, Y.; Feng, L.; Hu, X.; Dong, W. The Synthesis of Tetrazoles in Nanometer Aqueous Micelles at Room Temperature. *Eur. J. Org. Chem.* **2014**, *2014* (2), 436-441.

(4) Das, B.; Reddy, C. R.; Kumar, D. N.; Krishnaiah, M.; Narender, R. A Simple, Advantageous Synthesis of 5-Substituted 1H-Tetrazoles. *Synlett* **2010**, *3*, 391-394.

(5) Livingstone, K.; Bertrand, S.; Jamieson, C. One-Pot Suzuki-Hydrogenolysis Protocol for the Modular Synthesis of 2,5-Diaryltetrazoles. *J. Org. Chem.* **2020**, *85* (11), 7413-7423.

(6) Jahanshahi, R.; Akhlaghinia, B. Expanded perlite: an inexpensive natural efficient heterogeneous catalyst for the green and highly accelerated solvent-free synthesis of 5-substituted-1 H-tetrazoles using [bmim] N 3 and nitriles. *RSC Adv.* **2015**, *5* (126), 104087-104094.

(7) Glang, S.; Rieth, T.; Borchmann, D.; Fortunati, I.; Signorini, R.; Detert, H. Arylethynyl-Substituted Tristriazolotriazines: Synthesis, Optical Properties, and Thermotropic Behavior. *Eur. J. Org. Chem.* **2014**, *2014* (15), 3116-3126.

(8) Cantillo, D.; Gutmann, B.; Kappe, C. O. An Experimental and Computational Assessment of Acid-Catalyzed Azide-Nitrile Cycloadditions. *J. Org. Chem.* **2012**, *77* (23), 10882-10890.

ZnxCrCl_LDHs.zip
